# Discovery of coumaric acid derivatives hinted by coastal marine source to seek for uric acid lowering agents

**DOI:** 10.1080/14756366.2022.2163241

**Published:** 2023-01-11

**Authors:** Yu-Shun Yang, Bin Wang, Junzhong Liu, Qin Li, Qin-Cai Jiao, Pei Qin

**Affiliations:** a State Key Laboratory of Pharmaceutical Biotechnology, School of Life Sciences, Nanjing University, Nanjing, China; b Research and Development Center, Nanjing Shibeitai Biotechnology Co., Ltd., Nanjing, China

**Keywords:** Coastal marine sources, *Spartina alterniflora*, xanthine oxidase inhibition, lowering uric acid

## Abstract

In this work, a series of novel compounds **Spartinin C1-C24** were screened, synthesised and evaluated for inhibiting xanthine oxidase thus lowering serum uric acid level. The backbones were derived from the components of coastal marine source *Spartina alterniflora* and marketed drugs. The top hits **Spartinin C10** & **C22** suggested high inhibition percentages (78.54 and 93.74) at 10 μM dosage, which were higher than the positive control Allopurinol. They were low cytotoxic onto human normal hepatocyte cells. Treatment with **Spartinin C10** could lower the serum uric acid level to 440.0 μM in the hyperuricemic model mice (723.0 μM), comparable with Allopurinol (325.8 μM). **Spartinin C10** was more appreciated than Allopurinol on other serum indexes. The preliminary pharmacokinetics evaluation indicated that the rapid absorption, metabolism and elimination of **Spartinin C10** should be further improved. The discovery of pharmaceutical molecules from coastal marine source here might inspire the inter-disciplinary investigations on public health.

## Introduction

Natural products have been widely investigated to seek for functional molecules in the past century, and the famous examples included artemisinin and paclitaxel^1–3^. Simple extraction seemed not efficient and economic, which might also cause the species being endangered^4^. One potential solution was agricultural planting or acting as invasive species controlling^5^^,^^6^, while another further approach was conducting the modification onto the natural sourced compounds^7^^,^^8^. Moreover, the biosynthesis with microbes or processes with gene design have also become feasible methods^9^. The seeking procedure extended from inland to maritime space, thus to the coastal marine sources. During the controlling the over-growth of *Spartina alterniflora* Loisel, the extract of this coastal marine species was proved to show the effect of lowering uric acid and alleviating gout^10^. Further separation of the components indicated that coumaric acid was the main ingredient for the above activity via the inhibition of xanthine oxidase (XO)^11–13^. Although coumaric acid was found in many species, especially in some traditional Chinese medicines, the main merits for choosing *Spartina alterniflora* to produce coumaric acid included relatively high content, less interfering compounds, and most importantly, controlling the invasive species. Generally, it seemed a potential route to develop potent inhibitors of XO via the modification of natural backbone to improve the potency and safety^14^^,^^15^.

XO was responsible for catalysing the conversion of hypoxanthine to xanthine and then to uric acid, which was essential in the purine metabolism^16^^,^^17^. Inhibiting XO can lead to lower uric acid production, therefore XO has been regarded as the main target for treating gout and hyperuricemia^18^. XO inhibitors as marketed drugs for lowering uric acid included Allopurinol, Febuxostat and Topiroxostat^19–21^. Allopurinol was most widely used with the structural similarity to purine, thus it could cause various undesired adverse effects^22^. Free from the purine moiety, Febuxostat and Topiroxostat could bind into the active site of XO, which inspired the rational strategies such as fragment-based drug design (FBDD)^23^^,^^24^. Besides, during the discovery of potential XO inhibitors, both the potency and the safety should be seriously concerned.

**Figure 1.**
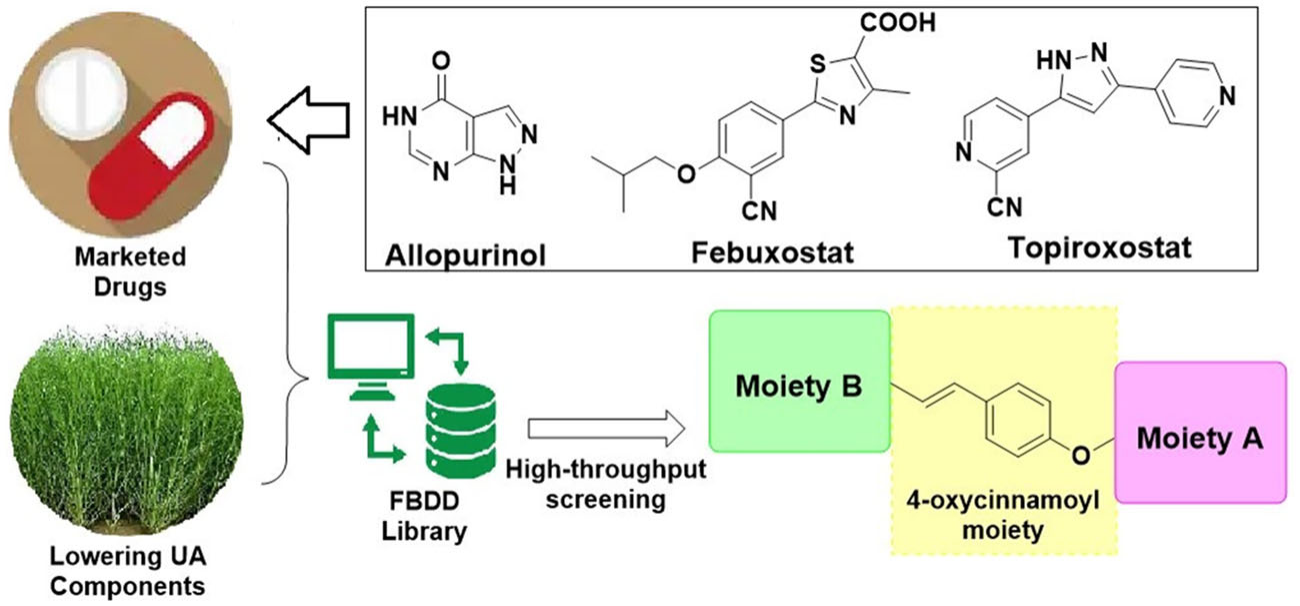
The sources and general patterns of the potential compounds **Spartinin C1–C24**.

Based on the *de novo* design concept, the structures of the novel compounds were composed from the selected fragments. The *Spartina alterniflora*-sourced natural components hinted the fragments such as *p*-coumaric acid, ferulic acid, chlorogenic acid, flavones, and glucoside; while the marketed XO inhibitors indicated the fragments including purine analogues, benzonitrile, thiazolecarboxylic acid, triazole, and cyanopyridine. In consideration of the similar antioxidant capability, some other high frequently reported fragments from antioxidant agents were also involved. With the decomposed fragments, the FBDD process was available to generate the library of compounds accordingly. Among the generated molecules, several modified coumaric acid derivatives obtained high ranks thus we conducted their synthesis, biological evaluation and preliminary druggability study in this work (Figure 1). The obtained series were named as **Spartinin C1–C24** (from “***Spartin****a alterniflora*” and “**c**oumaric acid”). Sharing the feature of 4-oxycinnamoyl moiety and sulphonate, functional groups such as fatty ester, aromatic amide and oxime were also included in the investigated series. Notably, sulphonate was chosen from the antioxidant agents on the basis of previous reports^25–27^. After the virtual screening, the discovery of uric acid lowering agent was conducted following the order of evaluating the XO inhibitory activity, cytotoxicity, mice serum indexes, and preliminary pharmacokinetic property in rats.

## Experimental section

### Materials and methods

All commercially available chemicals were directly used with no further purification after being purchased from Sigma Aldrich. The micro melting point apparatus (SGW X-4B, Shanghai, China) was used to test all the melting points. The DRX-600 spectrometer (Bruker, Rhenistetten-Forchheim, Germany) was used to record the ^1^H NMR and ^13^C NMR spectra. The AB SCIEX Triple-TOF 4600 (USA) was used to conduct the High-Resolution Mass Spectrometry (HRMS) analysis.

### Virtual screening and analysis

The fragments for generating the library were selected from the *Spartina alterniflora*-sourced natural components and the marketed XO inhibitors as well as high frequently reported ones from antioxidant agents. Then the library was built with the help of “Small molecules” module in Discovery Studio 3.5 software (BIOVIA, France) under the consideration of both FBDD and *de novo* concept. The virtual screening relied on the comparison of the binding patterns and interaction energy parameter from the molecular docking simulation between the generated ligands and the crystal structure of XO (PDB code: 3NVY) from the RCSB Protein Data Bank^28^. The docking simulation was conducted with “CDOCKER” module in Discovery Studio 3.5 software (BIOVIA, France) as referenced^29^. For each backbone, the rank depended on the rank of the fifth top hit and the manual verification of the binding pattern. In this work, three high-rank backbones were chosen for further screening of substitutes, and the top 24 hits were retained for synthesis and biological evaluation. The visual presentation of the binding patterns was conducted on Discovery Studio Visualiser 2021 software (BIOVIA, France).

### Chemistry

#### General synthesis of Spartinin C1–C24

**Figure 2.**
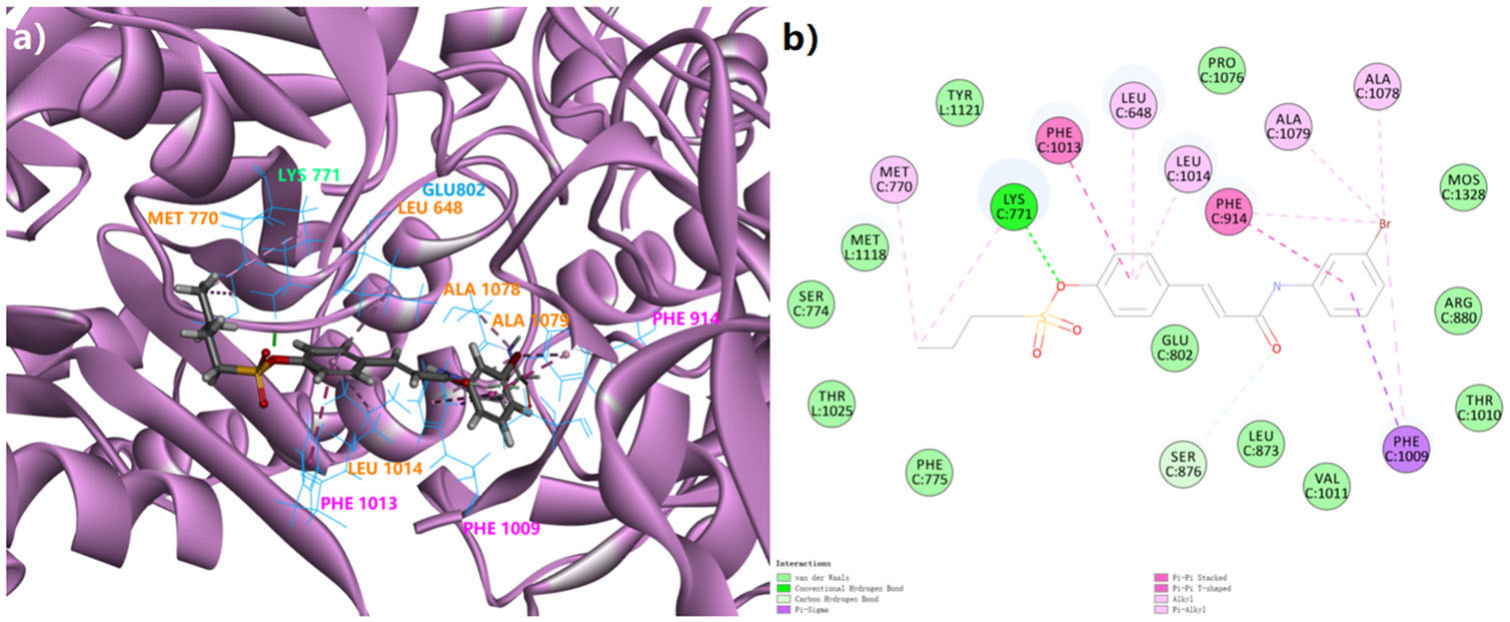
The 3D docking model (a) and the 2D binding pattern (b) of **Spartinin C10** into xanthine oxidase (PDB code: 3NVY). The H-bond was displayed as the dotted line between CYS 771 and the sulphonate. The π-related interactions were shown as other dotted lines.

The general synthetic route of **Spartinin C1–C24** was depicted in Figure 2 with the information of substituent. For **Spartinin C1–C17**, the reagent *p*-coumaric acid (10 mmol) and 1-butanesulfonyl chloride (10 mmol) was added into 15 ml dichloromethane (DCM) under stirring. Subsequently, 1 ml triethylamine (Et_3_N) was added dropwise. The mixture was kept being stirred at 0 °C for 5 h, and thin layer chromatography (TLC) was used to check the completion of the reaction. With the eluent of ethyl acetate (EA) and petroleum ether (PE) at a volume ratio of 1:10, the unpurified sulphonate intermediate **A1** was acquired from a column chromatography. Afterwards, **A1** (1 mmol) was added into the mixed solution with 10 ml DCM and 1 ml *N*,*N*-dimethylformamide (DMF). According to the target compounds, the corresponding fatty alcohols or aromatic amines (1 mmol) were added into the mixture, respectively. Then 1.5 mmol dicyclohexyl carbodiimide (DCC) and 0.5 mmol 4-dimethylaminopyridine (DMAP) were also added. The reaction continued for 4 h at room temperature, and the completion was checked by TLC. After the re-crystallization in a mixed solution (DMF: EtOH = 1:9, *v/v*), **Spartinin C1–C17** were obtained. On the other hand, for **Spartinin C18–C24**, *p*-coumaric acid or ferulic acid (10 mmol) and the corresponding sulphonyl chloride (10 mmol) was added into 15 ml DCM. After the addition of 1 ml Et_3_N, the mixture was kept being stirred at 0 °C for 5 h until the completion checked by TLC. The unpurified sulphonate intermediate **A2** was obtained from a column chromatography (EA:PE = 1:10, *v/v*). Afterwards, **A2** (1 mmol) and NH_2_OH·HCl (1 mmol) were added into 10 ml isopropanol (*i*-PrOH) under stirring, followed by the addition of 1 ml piperidine dropwise. The reaction continued for 8 h at 0 °C, and the completion was checked by TLC. The target compounds **Spartinin C18–C24** were obtained after the re-crystallization in a mixed solution (DMF: EtOH = 1:9, *v/v*).

Detailed physico-chemical properties of the synthesised compounds **Spartinin C1–C24**:

#### Methyl (E)-3–(4-((butylsulfonyl)oxy)phenyl)acrylate (Spartinin C1)

White solid, yielding 62.5%, melt point 38–39 °C. ^1^H NMR (600 MHz, DMSO-d_6_) *δ* 7.85 (d, *J* = 8.7 Hz, 2H, ArH), 7.69 (d, *J* = 16.1 Hz, 1H, =CH–), 7.37 (d, *J* = 8.7 Hz, 2H, ArH), 6.68 (d, *J* = 16.1 Hz, 1H, –CO–CH=), 3.74 (s, 3H, –OCH_3_), 3.57–3.52 (m, 2H, –CH_2_–), 1.85–1.76 (m, 2H, –CH_2_–), 1.48–1.42 (m, 2H, –CH_2_–), 0.91 (t, *J* = 7.4 Hz, 3H, -CH_3_).^13^C NMR (151 MHz, DMSO-d_6_) *δ* 166.97, 150.59, 143.53, 133.54, 130.63, 123.10, 119.26, 52.02, 50.15, 25.57, 21.06, 13.82. HRMS (ESI-TOF) *m/z*: [M + H]^+^ Calcd. for C_14_H_19_O_5_S 299.0953, Found 299.0953.

#### Ethyl (E)-3–(4-((butylsulfonyl)oxy)phenyl)acrylate (Spartinin C2)

Colourless liquid, yielding 58.7%. ^1^H NMR (600 MHz, DMSO-d_6_) *δ* 7.85 (d, *J* = 8.7 Hz, 2H, ArH), 7.68 (d, *J* = 16.1 Hz, 1H, =CH–), 7.37 (d, *J* = 8.7 Hz, 2H, ArH), 6.67 (d, *J* = 16.1 Hz, 1H, –CO–CH=), 4.20 (q, *J* = 7.1 Hz, 2H, –CH_2_–), 3.63–3.50 (m, 2H, –CH_2_–), 1.83–1.78 (m, 2H, -CH_2_-), 1.45 (dt, *J* = 14.8, 7.4 Hz, 2H, -CH_2_-), 1.27 (t, *J* = 7.1 Hz, 3H, –CH_3_), 1.00–0.89 (m, 3H, –CH_3_). ^13^C NMR (151 MHz, DMSO-d_6_) *δ* 166.50, 150.56, 143.35, 133.57, 130.60, 123.08, 119.60, 60.60, 50.16, 25.57, 21.07, 14.64, 13.81. HRMS (ESI-TOF) m/z: [M + H]^+^ Calcd. for C_15_H_21_O_5_S 313.1110, Found 313.1106.

#### Isobutyl (E)-3–(4-((butylsulfonyl)oxy)phenyl)acrylate (Spartinin C3)

White solid, yielding 64.3%, melt point 44–45 °C. ^1^H NMR (600 MHz, DMSO-d_6_) *δ* 7.86 (d, *J* = 8.7 Hz, 2H, ArH), 7.69 (d, *J* = 16.0 Hz, 1H, =CH–), 7.37 (d, *J* = 8.7 Hz, 2H, ArH), 6.69 (d, *J* = 16.0 Hz, 1H, –CO–CH=), 3.95 (d, *J* = 6.6 Hz, 2H, –CH_2_–), 3.68–3.49 (m, 2H, –CH_2_–), 1.98–1.93 (m, 1H,–CHR_2_), 1.83–1.78 (m, 2H, –CH_2_–), 1.48–1.42 (m, 2H, –CH_2_–), 0.96–0.89 (m, 9H, –(CH_3_)_3_). ^13^C NMR (151 MHz, DMSO-d_6_) *δ* 166.56, 150.56, 143.43, 133.56, 130.64, 123.07, 119.53, 70.42, 50.16, 27.82, 25.57, 21.07, 19.38, 13.82. HRMS (ESI-TOF) *m/z*: [M + H]^+^ Calcd. for C_17_H_25_O_5_S 341.1422, Found 341.1425.

#### (E)-4–(3-oxo-3-(phenylamino)prop-1-en-1-yl)phenylbutane-1-sulphonate (Spartinin C4)

Yellow power, yielding 38.4%, melt point 113–114 °C. ^1^H NMR (600 MHz, DMSO-d_6_) *δ* 10.25 (s, 1H, –NH–), 7.74 (d, *J* = 8.7 Hz, 2H, –ArH), 7.71 (d, *J* = 7.7 Hz, 2H, –ArH), 7.62 (d, *J* = 15.7 Hz, 1H, =CH–), 7.41 (d, *J* = 8.6 Hz, 2H, –ArH), 7.34 (t, *J* = 7.9 Hz, 2H, –ArH), 7.08 (t, *J* = 7.4 Hz, 1H, –ArH), 6.85 (d, *J* = 15.7 Hz, 1H, –CO–CH=), 3.61 − 3.51 (m, 2H, –CH_2_–), 1.84–1.79 (m, 2H, –CH_2_–), 1.49–1.43 (m, 2H, –CH_2_–), 0.92 (t, *J* = 7.4 Hz, 3H, –CH_3_). ^13^C NMR (151 MHz, DMSO-d_6_) *δ* 163.67, 150.05, 139.18, 139.18, 134.26, 129.89, 129.29, 123.90, 123.62, 123.24, 119.61, 50.12, 25.60, 21.07, 13.84. HRMS (ESI-TOF) *m/z*: [M + H]^+^ Calcd. for C_19_H_22_NO_4_S 360.1270, Found 360.1266.

#### (E)-4–(3-((2-fluorophenyl)amino)-3-oxoprop-1-en-1-yl)phenylbutane-1-sulphonate (Spartinin C5)

White power, yielding 38.5%, melt point 118–119 °C. ^1^H NMR (600 MHz, DMSO-d_6_) *δ* 9.99 (s, 1H, –NH–), 8.12 (q, *J* = 8.2 Hz, 1H, –ArH), 7.74 (d, *J* = 8.7 Hz, 2H, –ArH), 7.63 (d, *J* = 15.7 Hz, 1H, =CH–), 7.42 (d, *J* = 8.6 Hz, 2H, –ArH), 7.32–7.26 (m, 1H, –ArH), 7.23–7.14 (m, 2H, –ArH), 7.10 (dd, *J* = 15.8, 5.1 Hz, 1H, –CO–CH=), 3.65–3.51 (m, 2H, –CH_2_–), 1.84–1.79 (m, 2H, –CH_2_–), 1.49–1.43 (m, 2H, –CH_2_–), 0.92 (t, *J* = 7.4 Hz, 3H, –CH_3_). ^13^C NMR (151 MHz, DMSO-d_6_) *δ* 164.17, 154.51, 152.88, 150.12, 139.70, 134.25, 129.94, 126.71, 125.64, 124.89, 124.13, 123.96, 123.26, 115.98, 115.86, 50.13, 25.60, 21.07, 13.84. HRMS (ESI-TOF) *m/z*: [M + H]^+^ Calcd. for C_19_H_21_FNO_4_S 378.1175, Found 378.1173.

#### (E)-4–(3-((2-bromophenyl)amino)-3-oxoprop-1-en-1-yl)phenylbutane-1-sulphonate (Spartinin C6)

Yellow power, yielding 41.7%, melt point 117–118 °C. ^1^H NMR (600 MHz, DMSO-d_6_) *δ* 10.43 (s, 1H, –NH–), 8.09 (s, 1H, –ArH), 7.75 (d, *J* = 8.7 Hz, 2H, –ArH), 7.64 (d, *J* = 15.7 Hz, 1H, –ArH), 7.59 (d, *J* = 8.4 Hz, 1H, –ArH), 7.41 (d, *J* = 8.6 Hz, 2H, –ArH), 7.31 (t, *J* = 8.0 Hz, 1H, =CH–), 7.27 (d, *J* = 8.1 Hz, 1H, –ArH), 6.81 (d, *J* = 15.7 Hz, 1H, –CO–CH=), 3.61–3.53 (m, 2H, –CH_2_–), 1.84–1.79 (m, 2H, –CH_2_–), 1.49–1.43 (m, 2H, –CH_2_–), 0.92 (t, *J* = 7.4 Hz, 3H, –CH_3_). ^13^C NMR (151 MHz, DMSO–*d*_6_) *δ* 164.04, 150.17, 141.24, 139.80, 134.07, 131.29, 130.00, 126.49, 123.26, 123.17, 122.10, 122.01, 118.48, 50.14, 25.60, 21.08, 13.84. HRMS (ESI-TOF) *m/z*: [M + H]^+^ Calcd. for C_19_H_21_BrNO_4_S 438.0374, Found 438.0363.

#### (E)-4–(3-((2-iodophenyl)amino)-3-oxoprop-1-en-1-yl)phenylbutane-1-sulphonate (Spartinin C7)

Yellow power, yielding 36.3%, melt point 74–75 °C. ^1^H NMR (600 MHz, DMSO-d_6_) *δ* 9.63 (s, 1H, –NH–), 7.91 (dd, *J* = 7.9, 1.3 Hz, 1H, –ArH), 7.76 (d, *J* = 8.6 Hz, 2H, –ArH), 7.63 (d, *J* = 15.8 Hz, 1H, =CH–), 7.58 (d, *J* = 7.5 Hz, 1H, –ArH), 7.42 (t, *J* = 7.8 Hz, 3H, –ArH), 7.05–6.97 (m, 2H, –CO–CH=, –ArH), 3.59–3.54 (m, 2H, –CH_2_–), 1.82 (q, *J* = 7.5 Hz, 2H, –CH_2_–), 1.45 (dt, *J* = 14.8, 7.4 Hz, 2H, –CH_2_–), 0.92 (t, *J* = 7.4 Hz, 3H, –CH_3_). ^13^C NMR (151 MHz, DMSO-d_6_) *δ* 164.06, 150.12, 139.91, 139.65, 139.47, 134.24, 129.98, 129.14, 128.10, 127.50, 123.24, 118.55, 114.84, 50.14, 25.61, 21.08, 13.85. HRMS (ESI-TOF) *m/z*: [M + H]^+^ Calcd. for C_19_H_21_INO_4_S 486.0236, Found 486.0220.

#### (E)-4–(3-oxo-3-(o-tolylamino)prop-1-en-1-yl)phenylbutane-1-sulphonate (Spartinin C8)

White power, yielding 41.3%, melt point 133–134 °C. ^1^H NMR (600 MHz, DMSO-d_6_) *δ* 9.50 (s, 1H, –NH–), 7.75 (d, *J* = 8.5 Hz, 2H, –ArH), 7.67–7.54 (m, 2H, =CH–, –ArH), 7.41 (d, *J* = 8.6 Hz, 2H, –ArH), 7.24 (d, *J* = 7.4 Hz, 1H, –ArH), 7.19 (t, *J* = 7.3 Hz, 1H, –ArH), 7.10 (t, *J* = 7.4 Hz, 1H, –ArH), 7.00 (d, *J* = 15.7 Hz, 1H, –CO–CH=), 3.68–3.50 (m, 2H, –CH_2_–), 2.26 (s, 3H, –CH_3_), 1.84–1.79 (m, 2H, –CH_2_–), 1.49–1.43 (m, 2H, –CH_2_–), 0.92 (t, *J* = 7.4 Hz, 3H, –CH_3_). ^13^C NMR (151 MHz, DMSO-d_6_) *δ* 163.77, 150.00, 139.02, 136.66, 134.38, 131.52, 130.82, 129.85, 126.46, 125.51, 124.75, 123.70, 123.21, 50.13, 25.60, 21.08, 18.42, 13.84. HRMS (ESI-TOF) *m/z*: [M + H]^+^ Calcd. for C_20_H_24_NO_4_S 374.1426, Found 374.1420.

#### (E)-4–(3-((3-fluorophenyl)amino)-3-oxoprop-1-en-1-yl)phenylbutane-1-sulphonate (Spartinin C9)

White power, yielding 33.6%, melt point 120–121 °C. ^1^H NMR (600 MHz, DMSO-d_6_) *δ* 10.47 (s, 1H, –NH–), 7.74 (t, *J* = 10.3 Hz, 3H, –ArH), 7.64 (d, *J* = 15.7 Hz, 1H, =CH–), 7.41 (d, *J* = 8.7 Hz, 2H, –ArH), 7.40–7.36 (m, 2H, –ArH), 6.93–6.89 (m, 1H, –ArH), 6.82 (d, *J* = 15.7 Hz, 1H, –CO–CH=), 3.75–3.47 (m, 2H, –CH_2_–), 1.84–1.79 (m, 2H, –CH_2_–), 1.49–1.43 (m, 2H, –CH_2_–), 0.92 (t, *J* = 7.4 Hz, 3H, –CH_3_). ^13^C NMR (151 MHz, DMSO-d_6_) *δ* 164.07, 163.98, 163.42, 161.82, 160.16, 150.16, 139.78, 134.09, 130.97, 130.00, 123.26, 115.49, 110.44, 106.60, 50.13, 25.60, 21.07, 13.84. HRMS (ESI-TOF) *m/z*: [M + H]^+^ Calcd. for C_19_H_21_FNO_4_S 378.1175, Found 378.1176.

#### (E)-4–(3-((3-bromophenyl)amino)-3-oxoprop-1-en-1-yl)phenylbutane-1-sulphonate (Spartinin C10)

White power, yielding 35.9%, melt point 118–120 °C. ^1^H NMR (600 MHz, DMSO-d_6_) *δ* 10.43 (s, 1H, –NH–), 8.08 (s, 1H), 7.75 (d, *J* = 8.7 Hz, 2H), 7.67–7.56 (m, 2H, –ArH), 7.41 (d, *J* = 8.6 Hz, 2H, –ArH), 7.34–7.25 (m, 2H, =CH–, –ArH), 6.80 (d, *J* = 15.7 Hz, 1H, –CO–CH=), 3.63–3.51 (m, 2H, –CH_2_–), 1.84–1.79 (m, 2H, –CH_2_–), 1.48–1.43 (m, 2H, –CH_2_–), 0.92 (t, *J* = 7.4 Hz, 3H, –CH_3_). ^13^C NMR (151 MHz, DMSO-d_6_) *δ* 164.05, 150.17, 141.11, 139.82, 134.07, 131.31, 130.02, 126.51, 123.26, 123.16, 122.10, 122.01, 118.49, 50.14, 25.60, 21.07, 13.84. HRMS (ESI-TOF) *m/z*: [M + H]^+^ Calcd. for C_19_H_21_BrNO_4_S 438.0374, Found 438.0355.

#### (E)-4–(3-oxo-3-(m-tolylamino)prop-1-en-1-yl)phenylbutane-1-sulphonate (Spartinin C11)

Yellow power, yielding 47.5%, melt point 112–113 °C. ^1^H NMR (600 MHz, DMSO-d_6_) *δ* 10.17 (s, 1H, –NH–), 7.74 (d, *J* = 8.7 Hz, 2H, –ArH), 7.60 (d, *J* = 15.7 Hz, 1H, =CH–), 7.57–7.47 (m, 2H, –ArH), 7.41 (d, *J* = 8.6 Hz, 2H, –ArH), 7.22 (t, *J* = 7.8 Hz, 1H, –ArH), 6.94–6.87 (m, 1H, –ArH), 6.84 (d, *J* = 15.7 Hz, 1H, –CO–CH=), 3.62–3.51 (m, 2H, –CH_2_–), 2.30 (s, 3H, –CH_3_), 1.88–1.75 (m, 2H, –CH_2_–), 1.49–1.43 (m, 2H, –CH_2_–), 0.92 (t, *J* = 7.4 Hz, 3H, –CH_3_). ^13^C NMR (151 MHz, DMSO-d_6_) *δ* 163.60, 150.04, 139.06, 139.06, 138.44, 134.27, 129.87, 129.13, 124.63, 123.77, 123.24, 120.20, 116.92, 50.12, 25.60, 21.70, 21.07, 13.84. HRMS (ESI-TOF) *m/z*: [M + H]^+^ Calcd. for C_20_H_24_NO_4_S 374.1426, Found 374.1407.

#### (E)-4–(3-((4-fluorophenyl)amino)-3-oxoprop-1-en-1-yl)phenylbutane-1-sulphonate (Spartinin C12)

White power, yielding 47.3%, melt point 118–119 °C. ^1^H NMR (600 MHz, DMSO-d_6_) *δ* 10.32 (s, 1H, –NH–), 7.78–7.69 (m, 4H, –ArH), 7.62 (d, *J* = 15.7 Hz, 1H, =CH–), 7.41 (d, *J* = 8.7 Hz, 2H, –ArH), 7.19 (t, *J* = 8.9 Hz, 2H, –ArH), 6.81 (d, *J* = 15.7 Hz, 1H, –CO–CH=), 3.74–3.45 (m, 2H, –CH_2_–), 1.84–1.79 (m, 2H, –CH_2_–), 1.49–1.43 (m, 2H, –CH_2_–), 0.92 (t, *J* = 7.4 Hz, 3H, –CH_3_). ^13^C NMR (151 MHz, DMSO-d_6_) *δ* 163.57, 159.35, 157,76, 150.07, 139.26, 135.95, 134.20, 129.91, 123.24, 121.36, 115.95, 115.80, 50.12, 25.60, 21.07, 13.84. HRMS (ESI-TOF) *m/z*: [M + H]^+^ Calcd. for C_19_H_21_FNO_4_S 378.1175, Found 378.1170.

#### (E)-4–(3-((4-chlorophenyl)amino)-3-oxoprop-1-en-1-yl)phenylbutane-1-sulphonate (Spartinin C13)

Yellow power, yielding 35.2%, melt point 116–117 °C. ^1^H NMR (600 MHz, DMSO-d_6_) *δ* 10.39 (s, 1H, –NH–), 7.74 (dd, *J* = 8.6, 5.8 Hz, 4H, –ArH), 7.63 (d, *J* = 15.7 Hz, 1H, =CH–), 7.44–7.35 (m, 4H, –ArH), 6.82 (d, *J* = 15.7 Hz, 1H, –CO–CH=), 3.69–3.51 (m, 2H, –CH_2_–), 1.84–1.79 (m, 2H, –CH_2_–), 1.49–1.43 (m, 2H, –CH_2_–), 0.92 (t, *J* = 7.4 Hz, 3H, –CH_3_). ^13^C NMR (151 MHz, DMSO-d_6_) δ 163.78, 150.12, 139.55, 138.60, 138.49, 134.14, 129.96, 129.21, 127.46, 123.25, 121.15, 50.13, 25.60, 21.07, 13.84. HRMS (ESI-TOF) *m/z*: [M + H]^+^ Calcd. for C_19_H_21_ClNO_4_S 394.0880, Found 394.0877.

#### (E)-4–(3-((4-iodophenyl)amino)-3-oxoprop-1-en-1-yl)phenylbutane-1-sulphonate (Spartinin C14)

Yellow power, yielding 50.3%, melt point 119–120 °C. ^1^H NMR (600 MHz, DMSO-d_6_) *δ* 9.50 (s, 1H, –NH–), 7.59 (d, *J* = 15.8 Hz, 2H, –ArH), 7.47 (s, 1H, –ArH), 7.36 (d, *J* = 8.3 Hz, 1H, =CH–), 7.26 (dd, *J* = 22.0, 7.5 Hz, 2H, –ArH), 7.19 (t, *J* = 7.5 Hz, 1H, –ArH), 7.10 (t, *J* = 7.4 Hz, 1H, –ArH), 7.01 (d, *J* = 15.7 Hz, 1H, –CO–CH=), 3.91 (s, 3H, –OCH_3_), 3.56–3.49 (m, 2H, –CH_2_–), 2.26 (s, 3H, –CH_3_), 1.83 (p, J = 7.6 Hz, 2H, –CH_2_–), 1.47 (dt, J = 14.9, 7.4 Hz, 2H, –CH_2_–), 0.93 (t, *J* = 7.4 Hz, 3H, –CH_3_). ^13^C NMR (151 MHz, DMSO-d_6_) *δ* 163.89, 152.06, 139.48, 138.95, 136.79, 136.68, 135.44, 131.54, 130.82, 126.46, 125.53, 124.80, 123.79, 120.67, 112.93, 56.51, 51.10, 25.71, 21.15, 18.43, 13.89. HRMS (ESI-TOF) *m/z*: [M + H]^+^ Calcd. for C_21_H_26_NO_5_S 404.1532, Found 404.1515.

#### (E)-4–(3-oxo-3-(p-tolylamino)prop-1-en-1-yl)phenylbutane-1-sulphonate (Spartinin C15)

White power, yielding 39.2%, melt point 104–105 °C. ^1^H NMR (600 MHz, DMSO-d_6_) *δ* 10.16 (s, 1H, –NH–), 7.73 (d, *J* = 8.7 Hz, 2H, –ArH), 7.59 (dd, *J* = 12.0, 3.6 Hz, 3H, =CH–, –ArH), 7.40 (d, *J* = 8.6 Hz, 2H, –ArH), 7.14 (d, *J* = 8.3 Hz, 2H, –ArH), 6.83 (d, *J* = 15.7 Hz, 1H, –CO–CH=), 3.62–3.52 (m, 2H, –CH_2_–), 2.27 (s, 3H, –CH_3_), 1.84–1.78 (m, 2H, –CH_2_–), 1.48–1.43 (m, 2H, –CH_2_–), 0.92 (t, *J* = 7.4 Hz, 3H, –CH_3_). ^13^C NMR (151 MHz, DMSO-d_6_) *δ* 163.60, 150.04, 139.58, 139.06, 138.44, 134.27, 129.87, 129.13, 124.63, 123.77, 123.24, 120.20, 116.92, 50.12, 25.60, 21.70, 21.07, 13.84. HRMS (ESI-TOF) *m/z*: [M + H]^+^ Calcd. for C_20_H_24_NO_4_S.374.1426, Found 374.1419.

#### (E)-4–(3-((4-ethylphenyl)amino)-3-oxoprop-1-en-1-yl)phenylbutane-1-sulphonate (Spartinin C16)

White power, yielding 38.7%, melt point 108–109 °C. ^1^H NMR (600 MHz, DMSO-d_6_) *δ* 10.17 (s, 1H, –NH–), 7.73 (d, *J* = 8.7 Hz, 2H, –ArH), 7.68–7.51 (m, 3H, =CH–, –ArH), 7.40 (d, *J* = 8.6 Hz, 2H, –ArH), 7.17 (d, *J* = 8.4 Hz, 2H, –ArH), 6.83 (d, *J* = 15.7 Hz, 1H, –CO–CH=), 3.68–3.49 (m, 2H, –CH_2_–), 2.57 (q, *J* = 7.6 Hz, 2H, –CH_2_–), 1.84–1.79 (m, 2H, –CH_2_–), 1.49–1.43 (m, 2H, –CH_2_–), 1.17 (t, *J* = 7.6 Hz, 3H, –CH_3_), 0.92 (t, *J* = 7.4 Hz, 3H, –CH_3_). ^13^C NMR (151 MHz, DMSO-d_6_) *δ* 163.54, 150.01, 139.33, 138.92, 137.25, 134.31, 129.84, 128.48, 123.23, 119.77, 119.68, 50.11, 28.11, 25.60, 21.07, 16.17, 13.84. HRMS (ESI-TOF) *m/z*: [M + H]^+^ Calcd. for C_21_H_26_NO_4_S 388.1583, Found 388.1579.

#### (E)-4–(3-((4-methoxyphenyl)amino)-3-oxoprop-1-en-1-yl)phenylbutane-1-sulphonate (Spartinin C17)

White power, yielding 35.7%, melt point 117–118 °C. ^1^H NMR (600 MHz, DMSO-d_6_) *δ* 10.13 (s, 1H, –NH–), 7.73 (d, *J* = 8.6 Hz, 2H, –ArH), 7.63 (d, *J* = 9.0 Hz, 2H, –ArH), 7.59 (d, *J* = 15.7 Hz, 1H, =CH–), 7.40 (d, *J* = 8.6 Hz, 2H, –ArH), 6.92 (d, *J* = 9.0 Hz, 2H, –ArH), 6.81 (d, *J* = 15.7 Hz, 1H, –CO–CH=), 3.74 (s, 3H, –OCH_3_), 3.61–3.51 (m, 2H, –CH_2_–), 1.84–1.79 (m, 2H, –CH_2_–), 1.49–1.43 (m, 2H, –CH_2_–), 0.92 (t, *J* = 7.4 Hz, 3H, –CH_3_). ^13^C NMR (151 MHz, DMSO-d_6_) *δ* 163.29, 155.81, 149.97, 138.66, 134.35, 132.83, 129.80, 123.81, 123.23, 121.16, 121.07, 114.42, 55.63, 50.11, 25.60, 21.07, 13.84. HRMS (ESI-TOF) *m/z*: [M + H]^+^ Calcd. for C_20_H_24_NO_5_S 390.1375, Found 390.1362.

#### (E)-4–(3-(hydroxyamino)-3-oxoprop-1-en-1-yl)phenylbutane-1-sulphonate (Spartinin C18)

White power, yielding 30.4%, melt point 96–97 °C. ^1^H NMR (600 MHz, DMSO–d_6_) *δ* 10.81 (s, 1H, –OH), 9.09 (s, 1H, –NH–), 7.68 (d, *J* = 8.6 Hz, 2H, –ArH), 7.49 (d, *J* = 15.8 Hz, 1H, =CH–), 7.37 (d, *J* = 8.6 Hz, 2H, –ArH), 6.48 (d, *J* = 15.8 Hz, 1H, –CO–CH=), 3.56–3.52 (m, 2H, –CH_2_–), 1.83–1.78 (m, 2H, –CH_2_–), 1.48–1.42 (m, 2H, –CH_2_–), 0.92 (t, *J* = 7.4 Hz, 3H, –CH_3_). ^13^C NMR (151 MHz, DMSO-d_6_) *δ* 162.92, 149.83, 137.41, 134.37, 129.64, 123.14, 120.55, 50.06, 25.58, 21.07, 13.83. HRMS (ESI-TOF) *m/z*: [M + H] ^+^ Calcd. for C_13_H_18_NO_5_S 300.0905, Found 300.0906.

#### (E)-4–(3-(hydroxyamino)-3-oxoprop-1-en-1-yl)phenyl ethanesulfonate (spartinin C19)

White power, yielding 35.3%, melt point 105–106 °C. ^1^H NMR (600 MHz, DMSO–d_6_) *δ* 10.82 (s, 1H, –OH), 9.10 (s, 1H, –NH–), 7.68 (d, *J* = 8.6 Hz, 2H, –ArH), 7.50 (d, *J* = 15.8 Hz, 1H, =CH–), 7.37 (d, *J* = 8.7 Hz, 2H, –ArH), 6.48 (d, *J* = 15.8 Hz, 1H, –CO–CH=), 3.55 (q, *J* = 7.3 Hz, 2H, –CH_2_–), 1.38 (t, *J* = 7.3 Hz, 3H, –CH_3_). ^13^C NMR (151 MHz, DMSO-d_6_) *δ* 162.93, 149.85, 137.41, 134.38, 129.64, 123.11, 120.55, 45.16, 8.53. HRMS (ESI-TOF) *m/z*: [M + H]^+^ Calcd. for C_11_H_14_NO_5_S 272.0592, Found 272.0590.

#### (E)-4–(3-(hydroxyamino)-3-oxoprop-1-en-1-yl)phenyl 4-fluorobenzenesulfonate (Spartinin C20)

White power, yielding 32.8%, melt point 98–99 °C. ^1^H NMR (600 MHz, DMSO-d_6_) *δ* 10.82 (s, 1H, –OH), 9.11 (s, 1H, –NH–), 7.97 (dd, *J* = 8.9, 5.0 Hz, 2H, –ArH), 7.60 (d, *J* = 8.6 Hz, 2H, –ArH), 7.53 (t, *J* = 8.8 Hz, 2H, –ArH), 7.45 (d, *J* = 15.8 Hz, 1H, =CH–), 7.09 (d, *J* = 8.7 Hz, 2H, –ArH), 6.45 (d, *J* = 15.8 Hz, 1H, –CO–CH=). ^13^C NMR (151 MHz, DMSO-d_6_) *δ* 166.93, 165.24, 162.85, 149.72, 137.23, 134.70, 132.15, 132.08, 130.88, 129.59, 123.10, 120.82, 117.78, 117.62. HRMS (ESI-TOF) *m/z*: [M + H]^+^ Calcd. for C_15_H_13_FNO_5_S 338.0498, Found 338.0496.

#### (E)-4–(3-(hydroxyamino)-3-oxoprop-1-en-1-yl)-2-methoxyphenylbutane-1-sulphonate (Spartinin C21)

White power, yielding 37.1%, melt point 109–110 °C. ^1^H NMR (600 MHz, DMSO–d_6_) *δ* 10.79 (s, 1H, –OH), 9.09 (s, 1H, –NH–), 7.47 (d, *J* = 15.8 Hz, 1H, =CH–), 7.40 (s, 1H, –ArH), 7.31 (d, *J* = 8.3 Hz, 1H, –ArH), 7.20 (d, *J* = 8.2 Hz, 1H, –ArH), 6.51 (d, *J* = 15.8 Hz, 1H, –CO–CH=), 3.89 (s, 3H, –OCH_3_), 3.54–3.44 (m, 2H, –CH_2_–), 1.84–1.79 (m, 2H, –CH_2_–), 1.48–1.42 (m, 2H, –CH_2_–), 0.92 (t, *J* = 7.4 Hz, 3H, –CH_3_). ^13^C NMR (151 MHz, DMSO-d_6_) *δ* 162.98, 152.01, 138.76, 137.85, 135.45, 124.72, 120.69, 120.33, 112.88, 56.49, 51.04, 25.70, 21.14, 13.87. HRMS (ESI-TOF) *m/z*: [M + H]^+^ Calcd. for C_14_H_20_NO_6_S 330.1011, Found 330.1010.

#### (E)-4–(3-(hydroxyamino)-3-oxoprop-1-en-1-yl)-2-methoxyphenyl ethanesulfonate (Spartinin C22)

White power, yielding 32.7%, melt point 125–126 °C. ^1^H NMR (600 MHz, DMSO-d_6_) *δ* 10.79 (s, 1H, –OH), 9.09 (s, 1H, –NH–), 7.48 (d, *J* = 15.8 Hz, 1H, =CH–), 7.40 (s, 1H, –ArH), 7.31 (d, *J* = 8.3 Hz, 1H, –ArH), 7.20 (dd, *J* = 8.3, 1.7 Hz, 1H, –ArH), 6.51 (d, *J* = 15.8 Hz, 1H, –CO–CH=), 3.89 (s, 3H, –OCH_3_), 3.50 (q, *J* = 7.3 Hz, 2H, –CH_2_–), 1.39 (t, *J* = 7.3 Hz, 3H, –CH_3_). ^13^C NMR (151 MHz, DMSO-d_6_) *δ* 162.99, 152.03, 138.76, 137.85, 135.45, 124.66, 120.69, 120.32, 112.89, 56.50, 46.10, 8.55. HRMS (ESI-TOF) *m/z*: [M + H]^+^ Calcd. for C_12_H_16_NO_6_S 302.0698, Found 302.0694.

#### (E)-4–(3-(hydroxyamino)-3-oxoprop-1-en-1-yl)-2-methoxyphenyl 4-fluorobenzenesulfonate (Spartinin C23)

White power, yielding 31.1%, melt point 160–161 °C. ^1^H NMR (600 MHz, DMSO-d_6_) *δ* 10.79 (s, 1H, –OH), 9.10 (s, 1H, –NH–), 7.92 (dd, *J* = 8.9, 5.0 Hz, 2H, –ArH), 7.51 (t, *J* = 8.8 Hz, 2H, –ArH), 7.44 (d, *J* = 15.8 Hz, 1H, =CH–), 7.27 (s, 1H, –ArH), 7.18 (s, 2H, –ArH), 6.48 (d, *J* = 15.8 Hz, 1H, –CO–CH=), 3.56 (s, 3H, –OCH_3_). ^13^C NMR (151 MHz, DMSO-d_6_) *δ* 165.15, 162.92, 151.80, 138.30, 137.70, 135.87, 132.10, 131.73, 124.48, 120.95, 120.26, 117.29, 117.14, 112.78, 56.07. HRMS (ESI-TOF) *m/z*: [M + H]^+^ Calcd. for C_16_H_15_FNO_6_S 368.0604, Found 368.0602.

#### (E)-4–(3-(hydroxyamino)-3-oxoprop-1-en-1-yl)-2-methoxyphenyl 4-chlorobenzenesulfonate (Spartinin C24)

White power, yielding 35.8%, melt point 148–149 °C. ^1^H NMR (600 MHz, DMSO-d_6_) *δ* 10.80 (s, 1H, –OH), 9.10 (s, 1H, –NH–), 7.86–7.84 (m, 2H, –ArH), 7.75 (d, *J* = 8.7 Hz, 2H, –ArH), 7.44 (d, *J* = 15.8 Hz, 1H, =CH–), 7.28 (s, 1H, –ArH), 7.18 (s, 2H, –ArH), 6.49 (d, *J* = 15.8 Hz, 1H, –CO–CH=), 3.55 (s, 3H, –CH_3_). ^13^C NMR (151 MHz, DMSO-d_6_) *δ* 162.92, 151.76, 140.39, 138.26, 137.68, 135.94, 134.25, 130.62, 130.07, 124.46, 120.99, 120.29, 112.82, 56.05. HRMS (ESI-TOF) *m/z*: [M + H]^+^ Calcd. for C_16_H_15_ClNO_6_S 384.0308, Found 384.0311.

### Biological assays

#### The XO inhibitory assay

The inhibition on XO was tested with the enzyme-contained XO inhibition Kit (Yuanye Co. Ltd., Shanghai, China) which used the mechanism of measuring the absorption of the catalysis product uric acid at 293 nm on a micro-plate reader (Tecan Safire, Swiss). Allopurinol was used as the positive control, and the tested compounds were prepared as dimethyl sulfoxide (DMSO)-solution. The reacting buffer was basically Tris(hydroxymethyl)aminomethane hydrochloride (Tri-HCl). Since natural products was sometimes not obviously dose-dependent, the inhibition rate (%) at 10 µM dosage was used for evaluating the potency as referenced^30^^,^^31^. This assay was performed in triplicate.

#### The cytotoxicity assay

Since the most possible metabolism site of the investigated compounds was liver, human normal hepatocyte line L02 purchased from American Type Culture Collection (ATCC) was chosen in this assay. The cells were cultured in DMEM (Hyclone) with 10% foetal bovine serum (FBS, BI), 2 mmol/L of L-glutamine, 100 units/mL of penicillin-streptomycin (Sigma-Aldrich), 100 mg/mL streptomycin (Hyclone) at 37 °C in an atmosphere with 5% CO_2_. The quantitative test was based on a typical MTT (3–(4,5-dimethylthiazol-2-yl)-2,5-diphenyltetrazolium bromide)-based colorimetric method as referenced^32^^,^^33^. The calculation of the half cytotoxic concentration (CC_50_) values was based on the absorbance at 570 nm from a micro-plate reader (Tecan Safire, Swiss). This assay was performed in triplicate.

#### Testing serum indexes of hyperuricemic model mice

A dual-drug induction approach was developed as referenced to build the hyperuricemic mouse model to retain the serum uric acid in high level^25^^,^^34^. According to the incidence rate of hyperuricaemia, male Kunming mice (∼ 30 g) were chosen. The induction was conducted in one week. At first, hypoxanthine at the dosage of 500 mg/kg was intraperitoneally injected (i.p.) into the mice each day. At Day 7, after the injection of hypoxanthine, oteracil potassium at the dosage of 300 mg/kg was intraperitoneally injected (i.p.). The orbital blood was extracted after 90 min, and then the centrifugation at 15,000 rpm for 4 min was conducted to separate the serum. The level of UA was checked to guarantee the hyperuricemic model. Besides, compared with the model group, the blank group was injected with saline instead.

Based on the above procedure and previous reports^35^, the mice were set into five groups: (i) Blank group and (ii) Model group were prepared as above mentioned; (iii) Positive group: during the induction of model, allopurinol at the dosage of 5.0 mg/kg was intragastrically injected (i.g.) into the mice 30 min after the induction each day; iv) and v) Testing groups: the procedures were the same as that in iii) except for replacing allopurinol into **Spartinin C10** and **Spartinin C22**, respectively. The serum indexes including uric acid (UA), alanine aminotransferase (ALT), total bilirubin (TBIL), blood urea nitrogen (BUN) and creatinine (CREA) were recorded on the Beckman AU5800 automatic biochemical analyser (USA)^36^.

#### Preliminary pharmacokinetic assay

The preliminary pharmacokinetic study was conducted in male SD rats. Two groups with three mice each were set by random. In one group, **Spartinin C10** at the dosage of 3.0 mg/kg was intragastrically injected (i.g.; PO), while in the other group **Spartinin C10** at the dosage of 1.0 mg/kg was intravenously injected (i.v.; IV). The time points for collecting blood were set as 5 min, 15 min, 30 min, 1 h, 2 h, 4 h, 8 h and 24 h after the injection. The liquid chromatography tandem mass spectrometry (LC-MS/MS) method was pre-built. The system consisted of LC-20AD High Performance Liquid Chromatography (Shimazu, Japan) with Venusil C18 Plus column (2.1*50 mm, 5.0 μm, Agela, USA) and API 4000 mass spectrometry (AB, USA). The development of the quantification method followed the International Conference on Harmonisation of Technical Requirements for Registration of Pharmaceuticals for Human Use (ICH) guidelines. In the LC method, mobile phase A was distilled water and mobile phase B was acetonitrile. The rinse port wash solution was methanol. The flow rate was 0.700 ml/min and the initial back pressure was 8.9 Mpa. In the analysis, the approximate retention time of **Spartinin C10** was 1.29 min while that of the interior label (here was tolbutamide) was 1.10 min. Accordingly, the concentration of **Spartinin C10** in the serum samples was confirmed. The subsequent calculation was under a non-compartmental model statistical moment method with WinNonlin 7.0 software (USA). It was a pity that, in the PO group, the serum level of **Spartinin C10** was not detected after 15 min. Thus, the pharmacokinetics parameters in this group could not be calculated.

#### Ethical statement

Animal welfare and experimental procedures followed in accordance with the Guide for Care and Use of Laboratory Animals (National Institutes of Health, the United States) and the ethical requirements of Nanjing University. The approval (No. 202224063) was certificated by the Hubei Provincial Centre for Disease Control.

## Results and discussion

### In silico study

Before the practical experimental procedures, *in silico* study was initially performed. The generation of fragments based on the original compounds including *Spartina alterniflora*-sourced natural components (such as phenylpropanoids, medick sativa and chlorogenic acid analogues) as well as the marketed XO inhibitors (Allopurinol, Febuxostat and Topiroxostat). Some high frequently reported fragments from antioxidant agents were also involved. After the disassembling of the structures into basic units, the library of candidates was constructed by following *de novo* design and then growing scaffold. Since individual candidate indicated distinct interaction energy parameters, the rank of the fifth top hit was set as the index to evaluate the backbone to which it belonged. By manually examining the binding patterns, obvious unavailable ones were weeded out. After the high-rank backbones were obtained, the substituent was added onto them to enrich the diversity of the generated molecules, and then the molecular docking was conducted again to finally acquire the top 24 hits.

Here the *ad hoc* presentation of possible 3 D binding patterns of the top hit after biological evaluation was shown in advance. **Spartinin C10** was displayed to visualise their binding situations into xanthine oxidase Figure 2. Similar to that of the positive drugs according to the references^30^^,^^31^, **Spartinin C10** could possibly generate the π–σ or π–π interactions with PHE 1009 (π–σ, Distance 4.92 Å), PHE 914 (π–π, Distance 4.01 Å), and PHE 1013 (π–π, Distance 4.98 Å). A probable important and unique hydrogen bond could be formed between the sulphonate moiety and LYS 771 (Distance: 1.85 Å, Angle of donor-H-acceptor: 163.731°). Moreover, suitable length of the fatty chain and the introduced aryl group could lead to more interactions with the key residues such as MET 770, ALA 1078, and ALA 1079. Although the previously reported GLU 802 was not involved with strong interactions, it was still within the surrounding with *Van Der Waals* interactions. Then the other backbones (Figure S1) were also checked, but not given unnecessary details here. Therefore, the energy parameters and basic binding patterns of the generated compounds in this work were able to pass the preliminary virtual screening, and the synthesis procedure could be conducted accordingly.

### Chemistry synthesis

**Figure 3.**
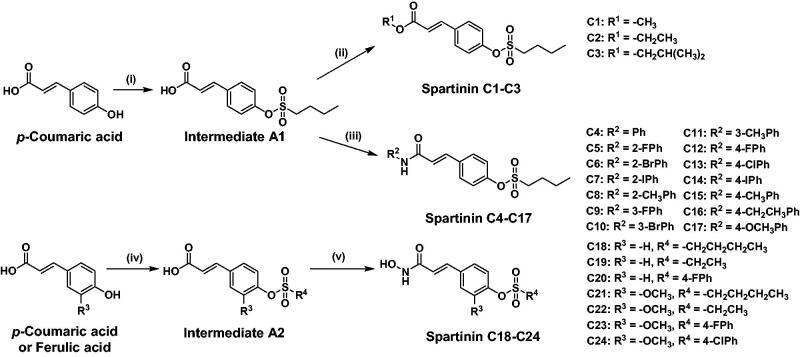
The synthetic route and the structures of **Spartinin C1–C24**. Reagents and conditions: (i) 1-butanesulfonyl chloride, Et_3_N, DCM, 0 °C, 5 h; (ii) R^1^-OH, DCC, DMAP, DCM, DMF, r.t. 4 h; (iii) R^2^-NH_2_, DCC, DMAP, DCM, DMF, r.t. 4 h; (iv) R^4^-SO_3_Cl, Et_3_N, DCM, 0 °C, 5 h; (v) NH_2_OH·HCl, Piperidine, *i*-PrOH, 0 °C, 8 h.

The general synthesis of the screened-out compounds **Spartinin C1–C24** was depicted in Figure 3. Sulphonate intermediate **A1** was prepared with *p*-coumaric acid and 1-butanesulfonyl chloride; while in the preparation of sulphonate intermediate **A2**, the reagents also included ferulic acid and more sulphonyl chloride species. Subsequently, the carboxyl group of **A1** reacted with various fatty alcohols and aromatic amines with the participation of dicyclohexyl carbodiimide (DCC) and 4-dimethylaminopyridine (DMAP) to yield the candidates **Spartinin C1–C17**. Meanwhile, intermediate **A2** was treated with hydroxylamine hydrochloride and piperidine in isopropyl alcohol to obtain the candidates **Spartinin C18–C24**. All the synthesised compounds were structurally confirmed with satisfactory data and spectra of ^1^H NMR, ^13^C NMR, and HRMS (Supplementary Materials).

### Biological evaluation

#### XO inhibitory activity

Herein, a XO inhibition Kit was used to check the XO inhibitory activity of the synthesised compounds **Spartinin C1–C24**. The mechanism of this kit was mimicking the catalysis procedure of XO on converting the substrate (here was xanthine) into uric acid. If the compound was potent, it could inhibit the catalysis procedure. According to the references, the inhibition rate (%) at 10 µM dosage was chosen as the index, and an over 40% value was set as the preliminary standard of being potential^30^^,^^31^. As shown in Table 1, two thirds of the investigated compounds indicated the inhibition percentage over 40%, which was comparable with the positive control Allopurinol. Here the preliminary structure-activity relationship (SAR) was analysed. Among the studied backbones, the oxime moiety inferred higher success rate to realise inhibitory efficiency than ester or amide moieties. For the ester group (**Spartinin C1–C3**), only **Spartinin C2** with ethyl showed potent inhibition (62.89%), whereas shorter (**C1**: 37.75%) or longer (**C3**: 37.02%) fatty chains were not beneficial. For the amide group (**Spartinin C4–C17**), compared with *ortho*- or *meta*-substituent, *para*-one was basically less potent. Among the *para*-substituted candidates, electron-donating ones with suitable size were beneficial (**C15** with methyl and **C16** with ethyl showed over 40% inhibition rate); whereas electron-withdrawing and bulky ones should be avoided (**C14** with iodol showed only 5.820% inhibition). Among the *ortho*- or *meta*-substituent, there was no obvious tendency but the candidates with bromo indicated attractive potency. Moreover, *meta*-bromo (**C10**: 78.54%) seemed more suitable than *ortho*-bromo (**C6**: 64.94%). For the oxime group (**Spartinin C18–C24**), with the same sulphonate substitute, the inhibitory activity has been improved with the addition of methoxy onto *p*-coumaric acid backbone to form ferulic acid backbone (**C21 **≥** C18; C22 **>** C19; C23 **>** C20**, “>” meant “better than”). Subsequently, it seemed that, with ferulic acid backbone, alky sulphonate was more appreciated than aryl sulphonate (**C21** & **C22**>**C23** & **C24**). Overall, **Spartinin C10** and **C22** were identified as the top hits to be further evaluated in animal models.

**Table 1.** The XO inhibitory activity and cytotoxicity of **Spartinin C1–C24**.

Code	XO/Inhibition percentage (%)	L02/CC_50_ (μM)	Code	XO/Inhibition percentage (%)	L02/CC_50_ (μM)
**C1**	37.75 ± 3.36	142.2 ± 3.07	**C13**	28.02 ± 0.508	30.41 ± 0.502
**C2**	62.89 ± 3.48	49.27 ± 0.789	**C14**	5.820 ± 0.701	36.44 ± 0.721
**C3**	37.02 ± 0.845	73.67 ± 7.36	**C15**	42.55 ± 2.03	31.62 ± 1.25
**C4**	47.52 ± 2.14	45.63 ± 1.14	**C16**	51.59 ± 0.984	54.18 ± 2.15
**C5**	38.82 ± 3.01	46.49 ± 1.46	**C17**	32.74 ± 3.78	100.2 ± 1.16
**C6**	64.94 ± 1.90	3.278 ± 0.045	**C18**	74.22 ± 6.78	9.729 ± 0.230
**C7**	41.62 ± 1.88	96.84 ± 1.34	**C19**	62.84 ± 1.77	14.12 ± 0.361
**C8**	42.39 ± 1.10	84.03 ± 1.41	**C20**	45.90 ± 1.38	1.377 ± 0.016
**C9**	15.07 ± 0.552	243.8 ± 8.75	**C21**	74.44 ± 1.33	3.882 ± 0.145
**C10**	78.54 ± 3.71	49.87 ± 4.42	**C22**	93.74 ± 1.59	25.28 ± 1.31
**C11**	47.75 ± 5.04	169.9 ± 16.3	**C23**	63.69 ± 1.42	3.411 ± 0.081
**C12**	27.15 ± 1.09	62.98 ± 0.808	**C24**	70.39 ± 3.67	79.27 ± 2.20
Allopurinol	46.56 ± 4.12	7.035 ± 0.495			

#### Cytotoxicity evaluation

In consideration of liver as the site where the metabolism of the compounds and the enzymatic process occurred, L02 human normal hepatocyte cell line was selected to check the cytotoxicity (Table 1). The cytotoxicity of the oxime derivatives was obviously higher than that of the ester or amide derivatives. In the ester and amide group, all the tested compounds (except for **C6**) showed low cytotoxicity with the CC_50_ values over 10 μM, compared with that of the control Allopurinol (6.842 μM); while in the oxime group, only **C20**, **C22**, and **C24** showed over 10 μM CC_50_ values. In combination of the inhibitory activity and cytotoxicity, **Spartinin C10** and **C22** were still potential for further investigation.

#### Changes in serum indexes of hyperuricemic model mice

As detailed in the experimental section^25^^,^^34–36^, the dual-drug (hypoxanthine and oteracil potassium) induction by intraperitoneal injection (i.p.) was conducted on male Kunming mice to build the hyperuricemic mouse model. In comparison, the blank group was intraperitoneally injected (i.p.) with saline instead. In the positive group, during the induction of hyperuricemic model, the mice were intragastrically injected (i.g.) with 5.0 mg/kg Allopurinol each day. In the testing groups, with the same dosage, the treatment was the same as that of the positive group except for replacing Allopurinol with **Spartinin C10** and **Spartinin C22**, respectively. As shown in Table 2, after the 7 day-treatment, the level of serum indexes including uric acid (UA), alanine aminotransferase (ALT), total bilirubin (TBIL), blood urea nitrogen (BUN) and creatinine (CREA) was tested. The selection of these indexes obeyed the principle of liver-relating and conciseness. Most significantly, the UA level was analysed to infer the potency. Before the induction, the UA level of the blank group stayed at 142.6 μM. In the hyperuricemic model mice, the UA level raised to 723.0 μM. After the treatment with allopurinol, **Spartinin C10** or **Spartinin C22**, the UA level decreased to 325.8 μM, 440.0 μM or 620.3 μM, respectively. Obviously, **Spartinin C10** lowered the serum UA level and the efficiency was comparable with the positive control Allopurinol. Meanwhile, since one of the common advantages of natural compounds and their derivatives was biological safety, here the changes of other indexes should not be ignored. For ALT, the model indicated a remarkable decrease from 61.48 U/L to 33.51 U/L. None of the treating agents (Allopurinol, **Spartinin C10** or **Spartinin C22**; ∼30 U/L) could bring obvious changes to the ALT level of model. For TBIL and BUN, the hyperuricemic model mice showed an up-regulation (TBIL: from 8.704 μM to 12.84 μM; BUN: from 21.55 mg/dL to 53.44 mg/dL). Allopurinol caused the decrease in these two indexes (TBIL: 10.86 μM; BUN: 45.32 mg/dL), while **Spartinin C10** (TBIL: 7.328 μM; BUN: 29.91 mg/dL) and **C22** (TBIL: 10.80 μM; BUN: 29.56 mg/dL) indicated higher efficiency in lowering these indexes to be closer to the normal level. For CREA, the model mice exhibited a higher level (60.49 μM) than the blank group (22.32 μM), while the treatment of Allopurinol caused further over-production (75.47 μM). On the contrary, **Spartinin C10** and **C22** down-regulated this index to 34.17 μM and 46.94 μM, respectively. In brief, **Spartinin C10** indicated comparable efficiency in lowering UA level with Allopurinol, and the performances on other biological indexes were much better.

**Table 2.** The serum indexes of mice with different treatments.

Group	UA (μM)	ALT (U/L)	TBIL (μM)	BUN (mg/dL)	CREA (μM)
Blank	149.2 ± 11.39	61.48 ± 10.18	8.704 ± 0.2502	21.55 ± 3.311	22.32 ± 2.112
Model	723.0 ± 181.9^a^	29.44 ± 7.454^a^	12.84 ± 4.363^a^	53.44 ± 1.644^a^	60.49 ± 6.011^a^
Allopurinol	325.8 ± 91.37^d^	33.51 ± 8.487^a^	10.86 ± 1.772^a^	45.32 ± 7.396^b^	75.47 ± 19.73^a^
**C10**	440.0 ± 142.7^c^	26.08 ± 8.731^a^	7.328 ± 1.898^a^	29.91 ± 1.722^d^	34.17 ± 4.976^c^
**C22**	620.3 ± 105.1^a^	25.65 ± 4.259^a^	10.80 ± 4.376^a^	29.56 ± 4.001^d^	46.94 ± 4.477^b^

Significance analysis compared with Model group (one-side Student’s *t* test): ^a^*p* ≥ 0.05; ^b^*p* < 0.05; ^c^*p* < 0.005; ^d^*p* < 0.001.

#### Preliminary pharmacokinetics analysis

**Figure 4.**
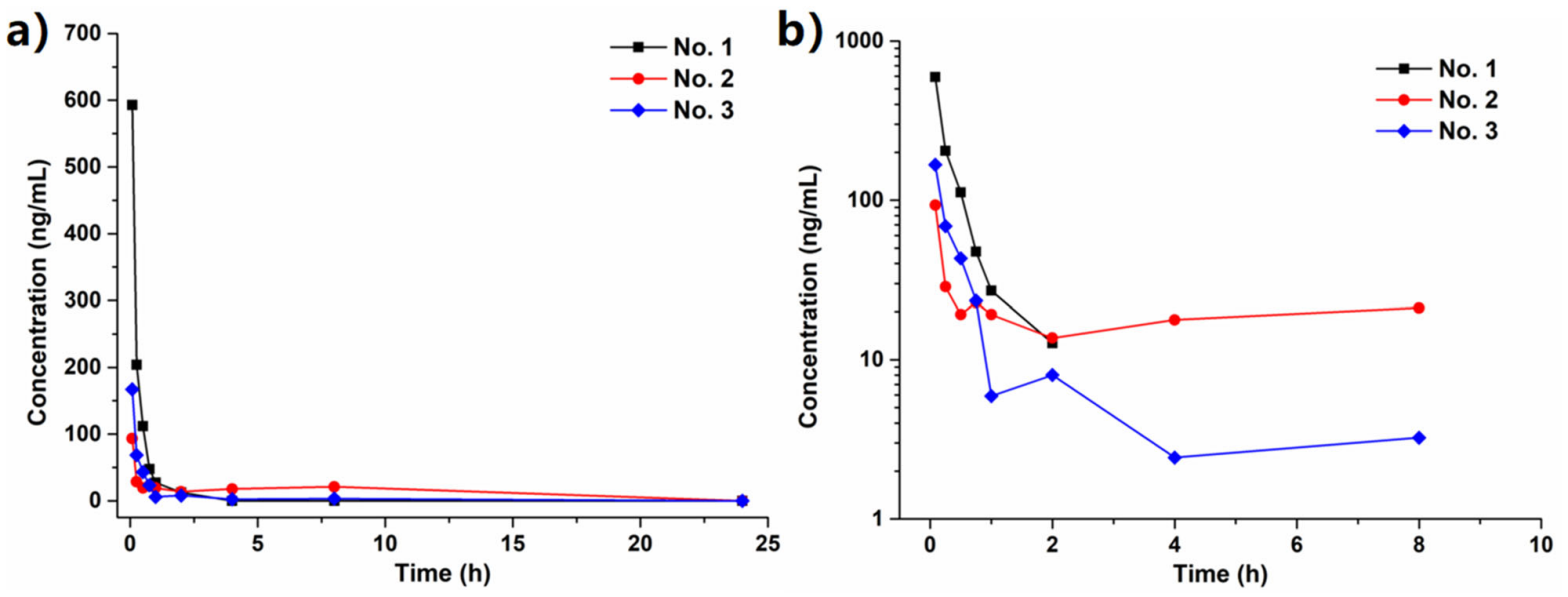
(a) The concentration of **Spartinin C10** in the rat serum samples with intravenous injection (i.v.) at 1.0 mg/kg. (b) The log scale of (a). Time points: 5 min, 15 min, 30 min, 1 h, 2 h, 4 h, 8 h and 24 h.

After convincing the potential potency in lowering the serum UA level, the preliminary pharmacokinetics evaluation was conducted. For fully indicating the *in vivo* behaviour of **Spartinin C10**, its concentration in rat serum samples was measured during 24 h. Two modes of administration were applied. One was the intragastric injection (i.g., or “PO” for “per os”) at a dosage (D) of 3.0 mg/kg, while the other was the intravenous injection (i.v., or “IV”) at a dosage of 1.0 mg/kg. In the PO group, the serum level of **Spartinin C10** could not be detected after 15 min. In the IV group, the time-concentration curve was depicted in Figure 4 and the pharmacokinetics parameters were listed in Table 3. The absorption, metabolism and elimination of **Spartinin C10** took about 8 h, which was relatively short. Moreover, from the peak time (*T*_max_) and elimination half-life (*t*_1/2_) values, the serum concentration of **Spartinin C10** decreased rapidly within the first hour. According to the peak concentration (*C*_max_) and the area under drug concentration-time curve (AUC) values, **Spartinin C10** seemed remained a low but workable concentration. Thus, the potency could be further improved by optimising the pharmacokinetic properties.

**Table 3.** The pharmacokinetics indexes of **Spartinin C10** in rats through intravenous injection.

Group	*t*_1/2_ (h)	*T*_max_ (h)	*C*_max_ (ng/mL)	*AUC*_0–t_ (h*ng/mL)	*AUC*_0–∞_ (h*ng/mL)
IV	0.5380 ± 0.228	0.0833 ± 0.000	284 ± 270	159.0 ± 65.0	184.0 ± 74.4

## 4. Conclusions

In summary, in this work, a series of novel compounds **Spartinin C1–C24** were synthesised and evaluated for inhibiting xanthine oxidase thus lowering serum uric acid level. A majority of the investigated compounds indicated the inhibition percentage over 40%, which was comparable with the positive control Allopurinol. At a common 10 μM dosage, the top hits **Spartinin C10** and **Spartinin C22** suggested high inhibition percentages as 78.54 and 93.74, respectively. They also exhibited low cytotoxicity onto human normal hepatocyte cells. By treating the hyperuricemic model mice with **Spartinin C10**, the serum UA level could be lowered to 440.0 μM from 723.0 μM, which was comparable with Allopurinol (325.8 μM). For the other serum indexes, **Spartinin C10** was more beneficial than Allopurinol on TBIL, BUN and CREA. The preliminary pharmacokinetics evaluation indicated that the absorption, metabolism and elimination of **Spartinin C10** was rapid, and the serum concentration was low but workable. This information might lead to further improvement on pharmacokinetics properties. Therefore, discovery of pharmaceutical molecules from coastal marine source with rational procedures might inspire the inter-disciplinary investigations on public health.

## Supplementary Material

Supplemental Material
