# Supplementary material for: Discovery of coumaric acid derivatives hinted by coastal marine source to seek for uric acid lowering agents
Source: J Enzyme Inhib Med Chem. 2023 Jan 11;38(1):2163241. doi: 10.1080/14756366.2022.2163241 (PMC9848256; doi:10.1080/14756366.2022.2163241)
Supplement: Supplemental Material [file IENZ_A_2163241_SM5160.pdf]

Discovery of coumaric acid derivatives hinted by coastal marine source to seek for uric acid lowering agents

Yu-Shun Yang[*,a,b], Bin Wang[a], Junzhong Liu[a], Qin Li[a], Qin-Cai Jiao[*,a] and Pei Qin[*,a,b]

[a] State Key Laboratory of Pharmaceutical Biotechnology, School of Life Sciences, Nanjing University, Nanjing, 210023, China;

[b] Research and development center, Nanjing Shibeitai Biotechnology Co., Ltd., Nanjing 210003, China.

[*]Corresponding authors. Tel. & Fax: +86-25-83685469; E-mail: jiaoqc@nju.edu.cn; qinpei@nju.edu.cn; ys_yang@nju.edu.cn.

Supplementary Figure

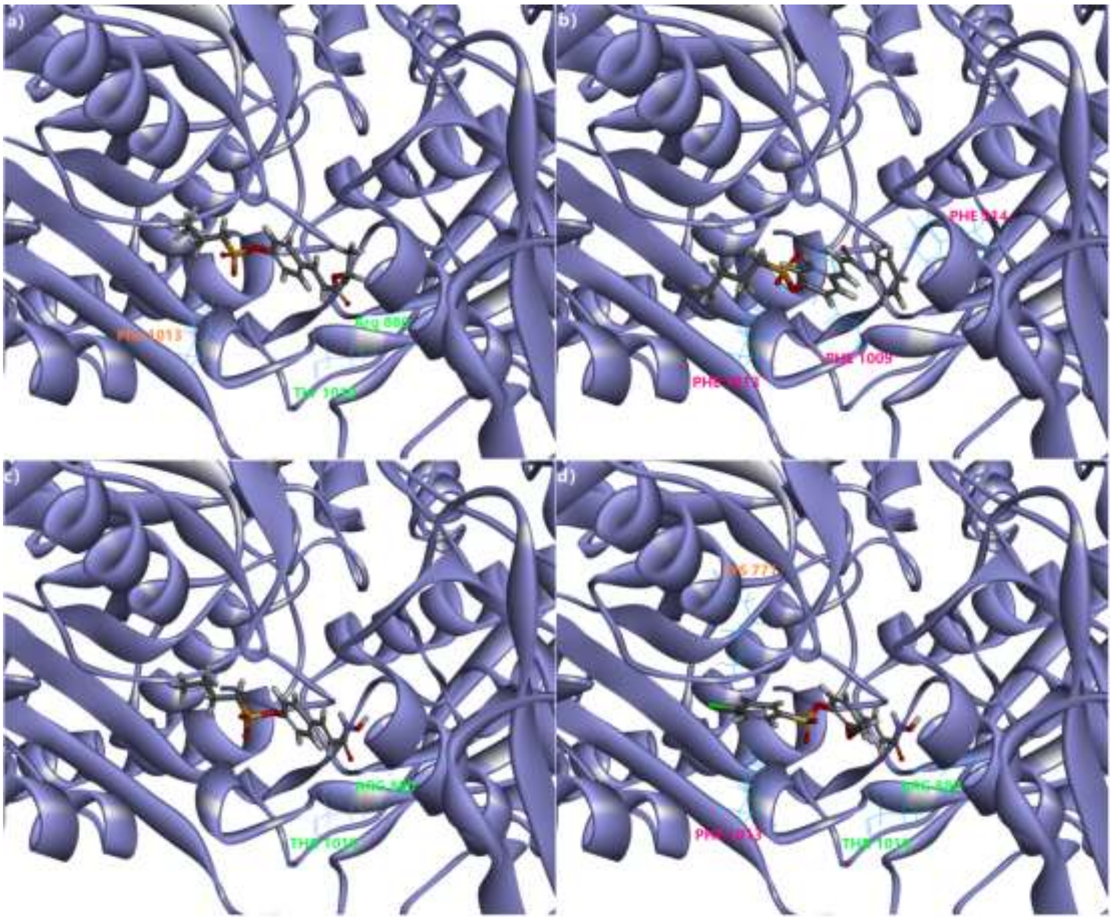

Figure S1. The 3D docking model of the various backbones represented by (a) **Spartinin C2**, (b) **Spartinin C4**, (c) **Spartinin C18**, and (d) **Spartinin C24**, respectively. The hydrogen bond-related residues were labeled in green, the π-cation and π-sulfur interaction-related residues were labeled in orange, while the π-π stacked interaction-related residues were labeled in dark pink.

NMR & HRMS spectra

Spartinin C1

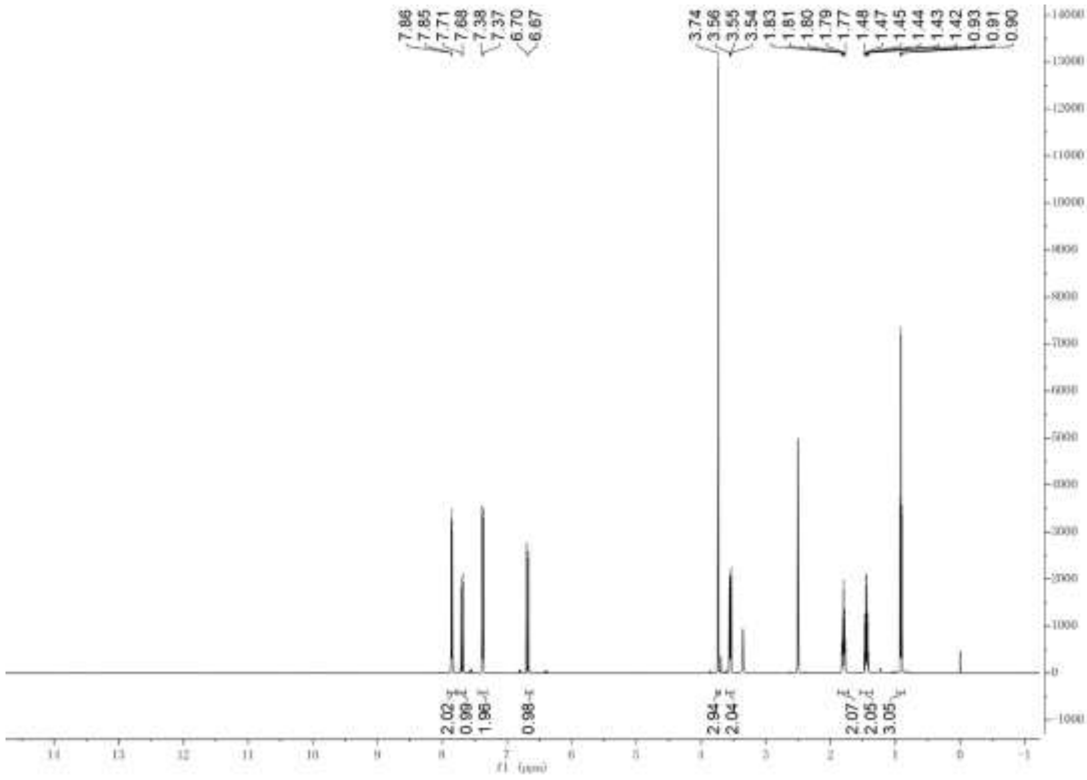

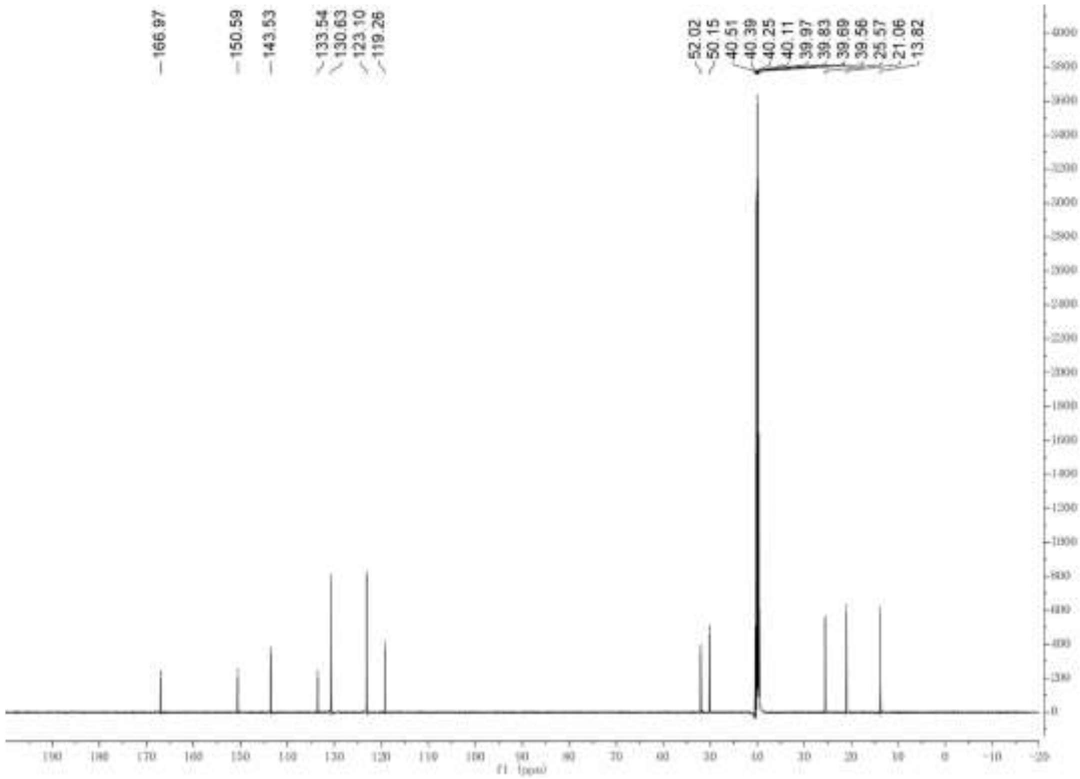

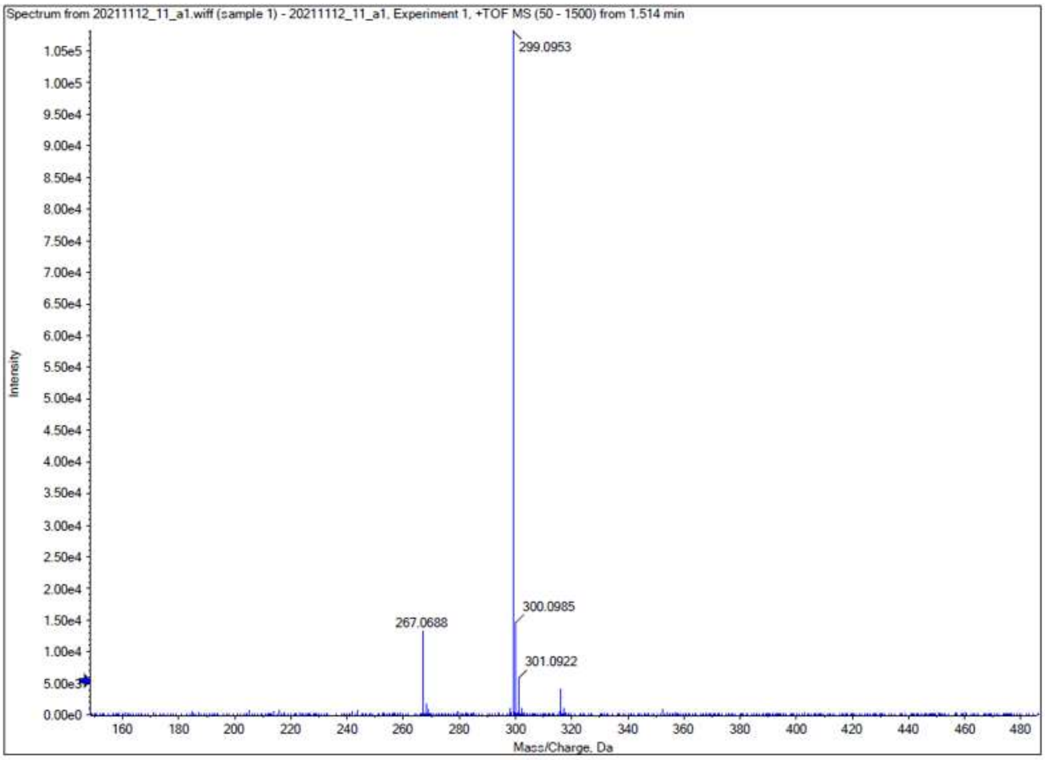

Spartinin C2

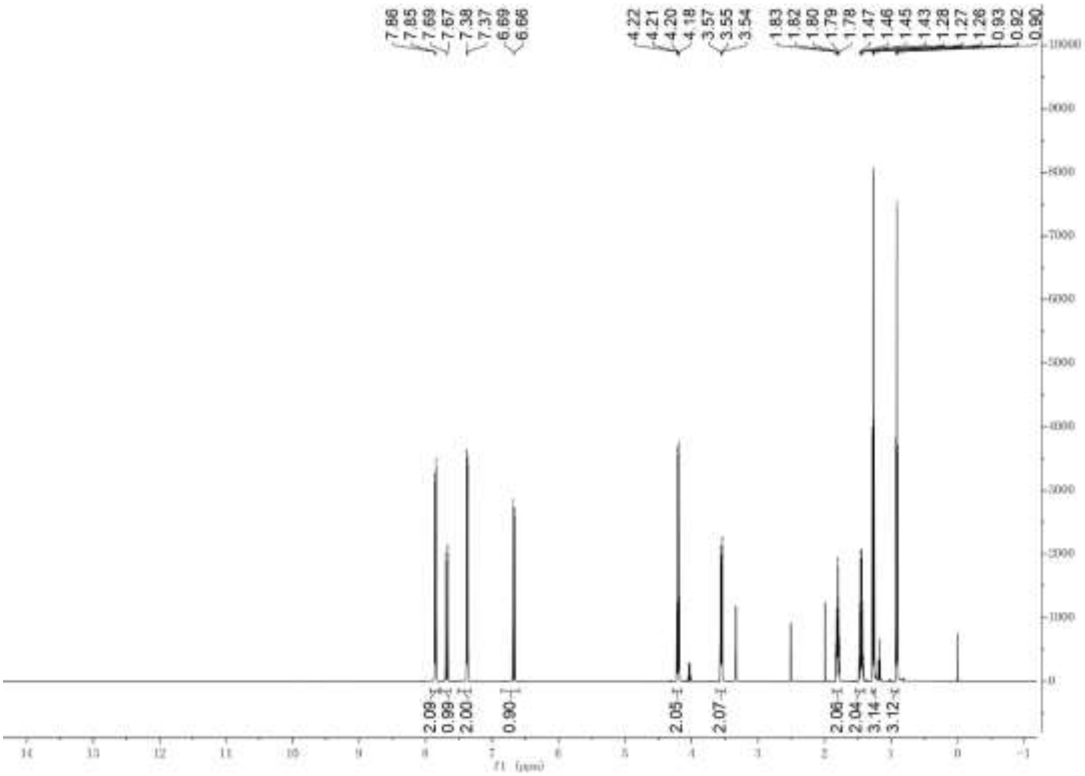

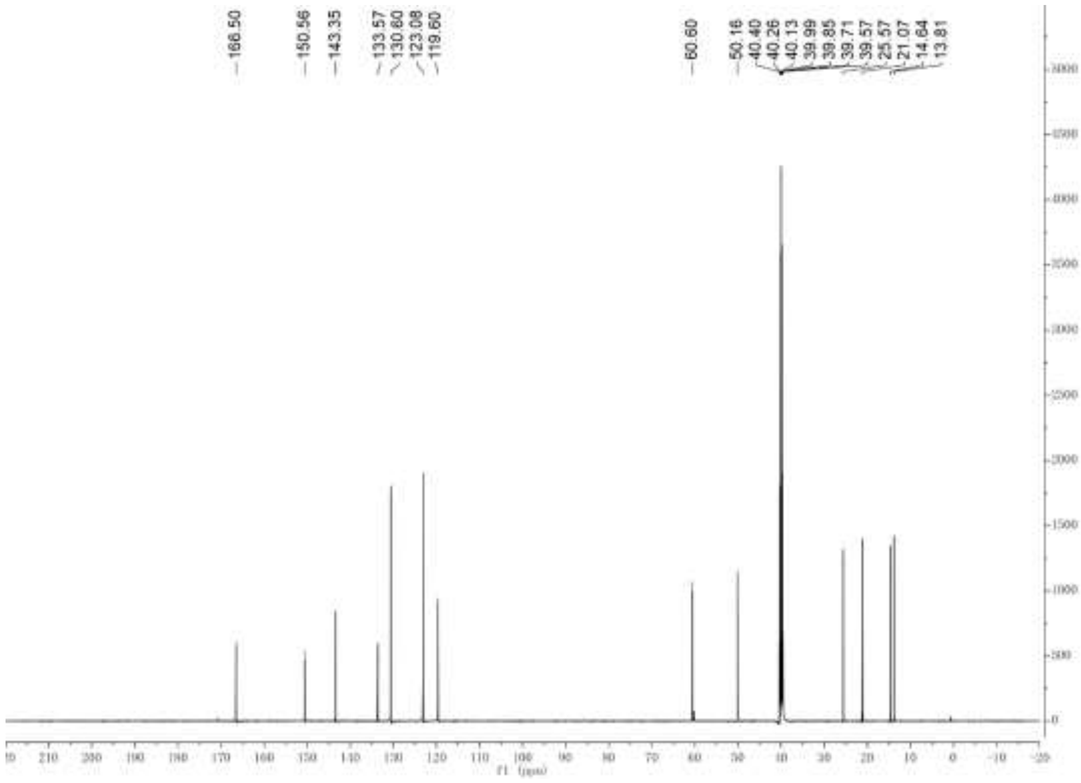

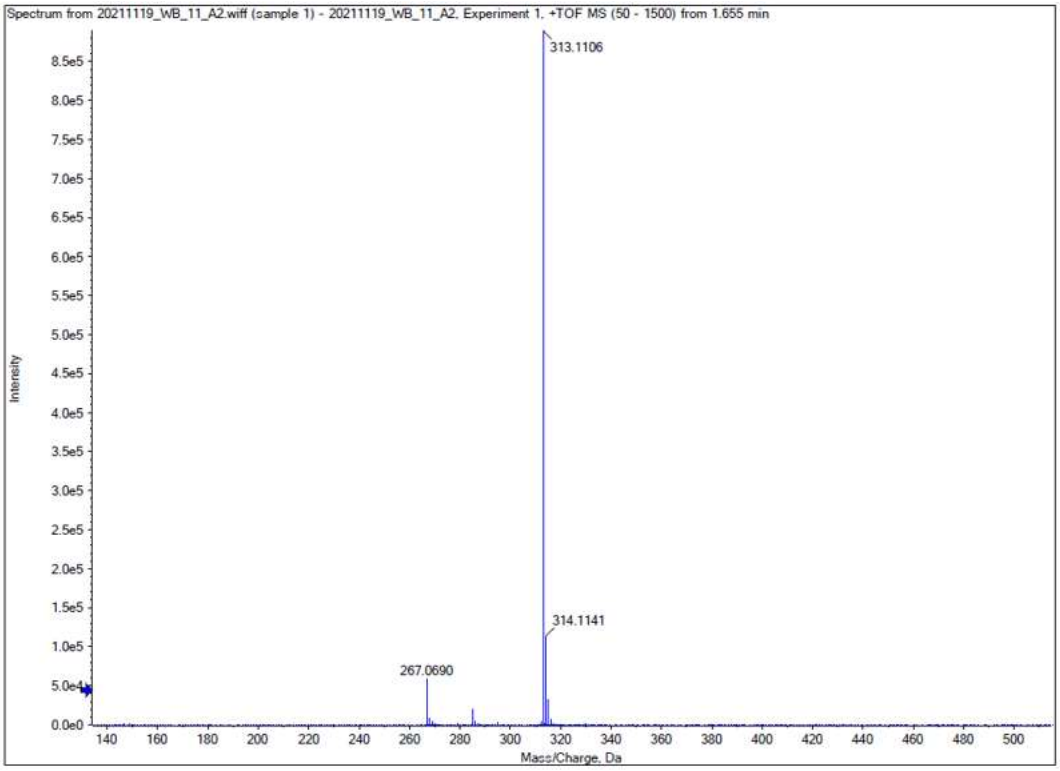

Spartinin C3

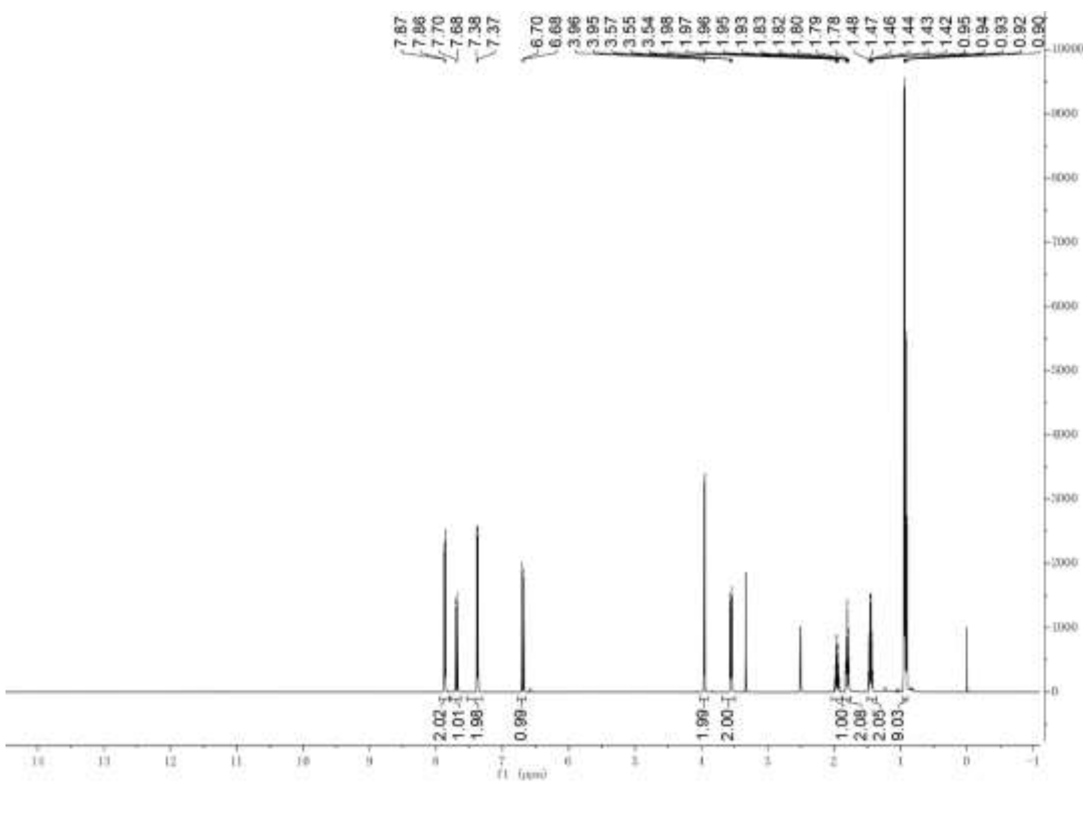
7.87
7.86
7.70
7.68
7.38
7.37
6.70
6.68
3.96
3.95
3.57
3.55
3.54
1.98
1.97
1.96
1.95
1.93
1.83
1.82
1.80
1.79
1.78
1.48
1.47
1.46
1.44
1.43
1.42
0.95
0.94
0.93
0.92
0.90
2.02
1.01
1.98
0.99
1.99
2.00
1.00
2.08
2.05
9.03
f1 (ppm)

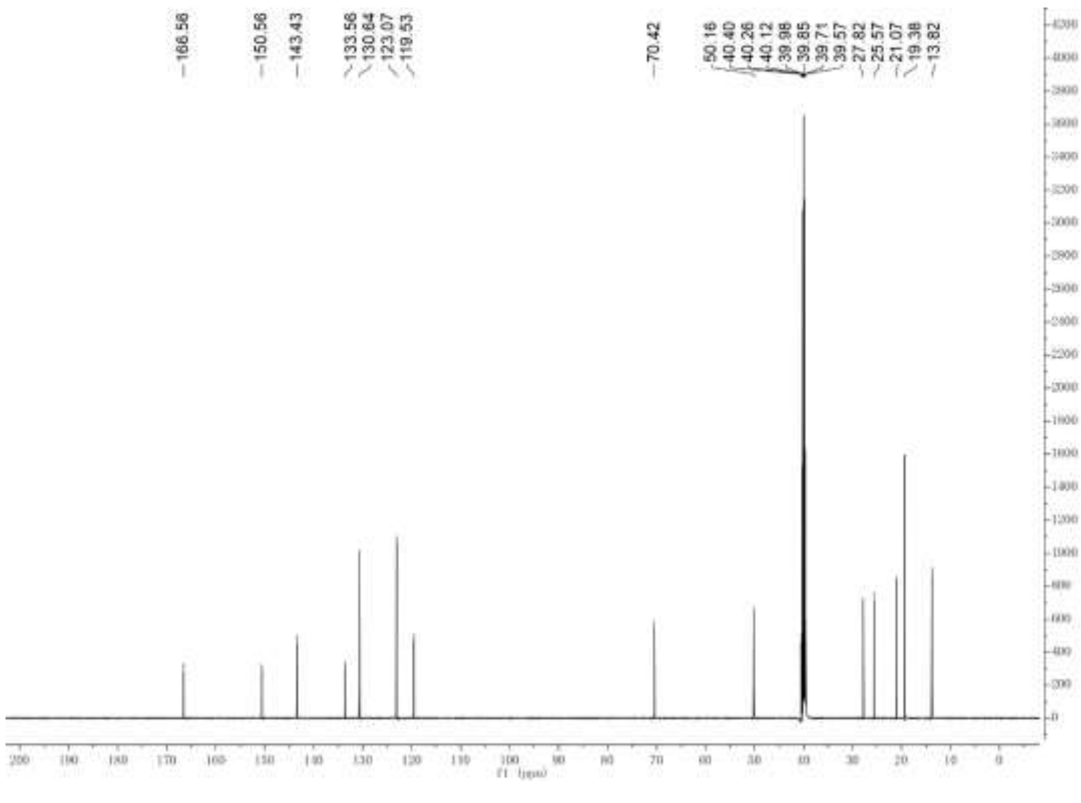
166.56
150.56
143.43
133.56
130.64
123.07
119.53
70.42
50.16
40.40
40.26
40.12
39.98
39.85
39.71
39.57
27.82
25.57
21.07
19.38
13.82
f1 (ppm)

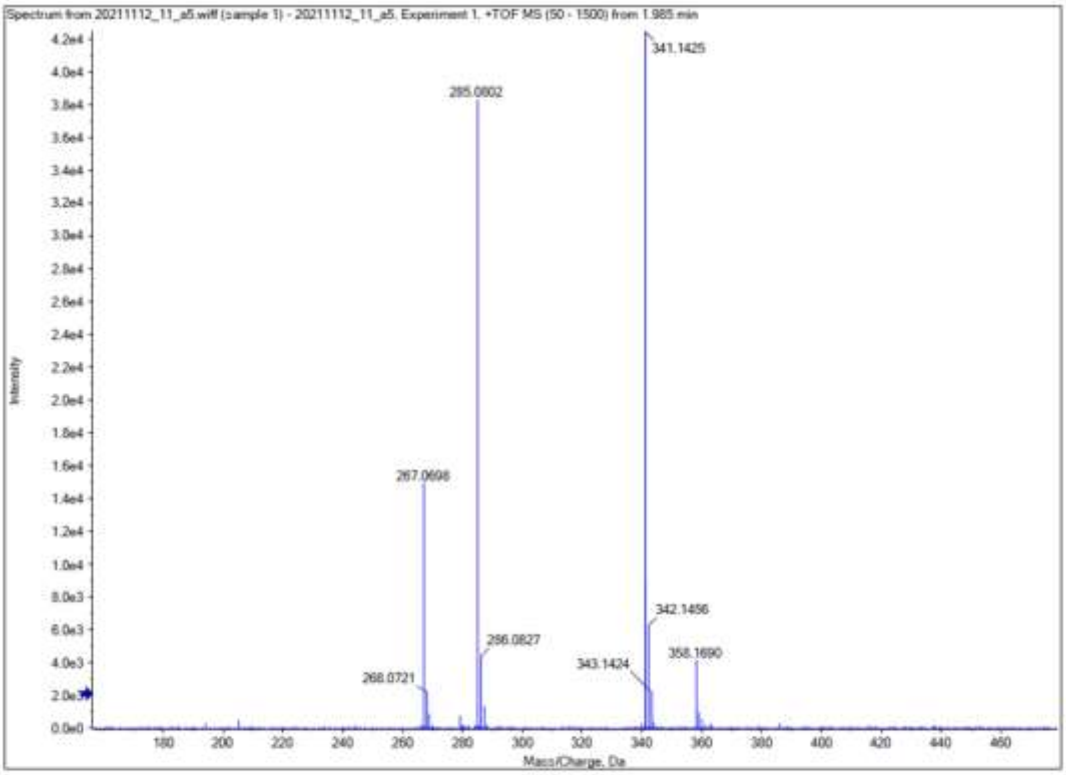

Spartinin C4

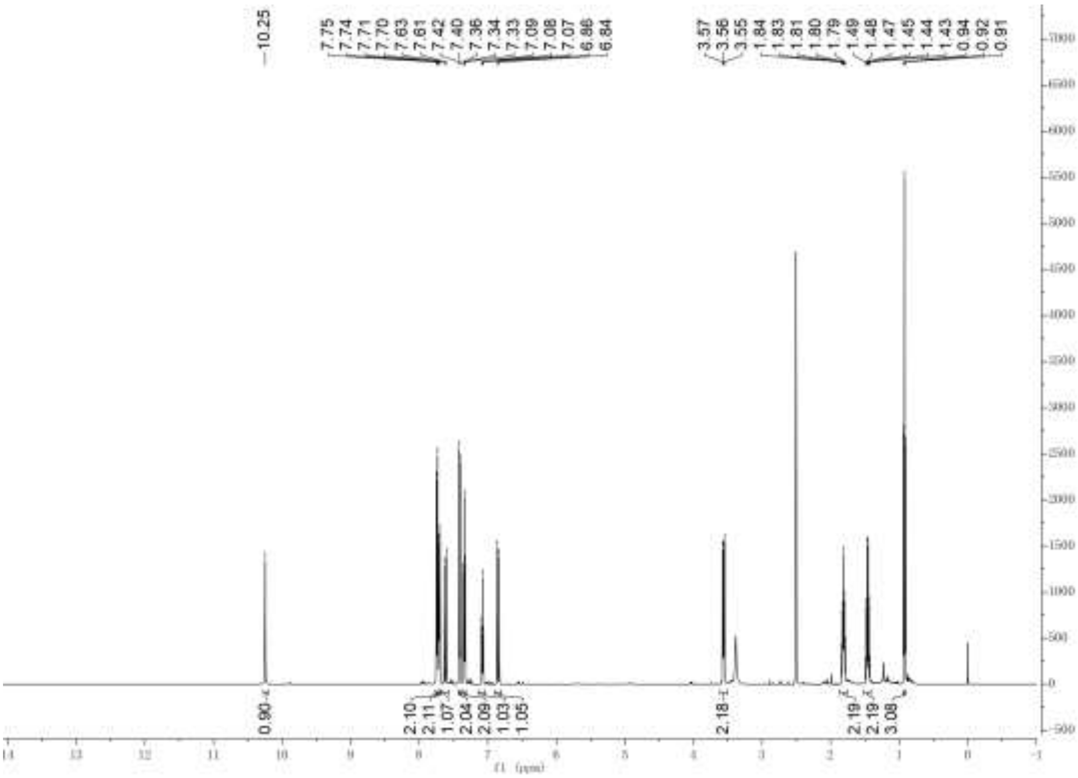

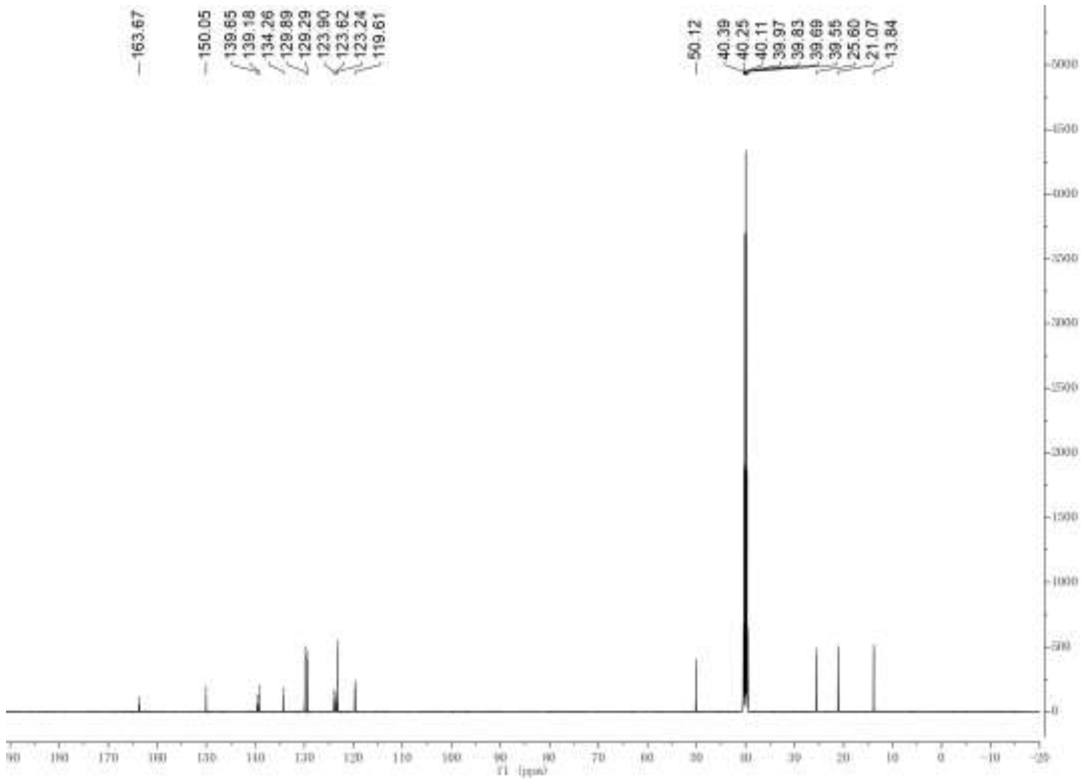

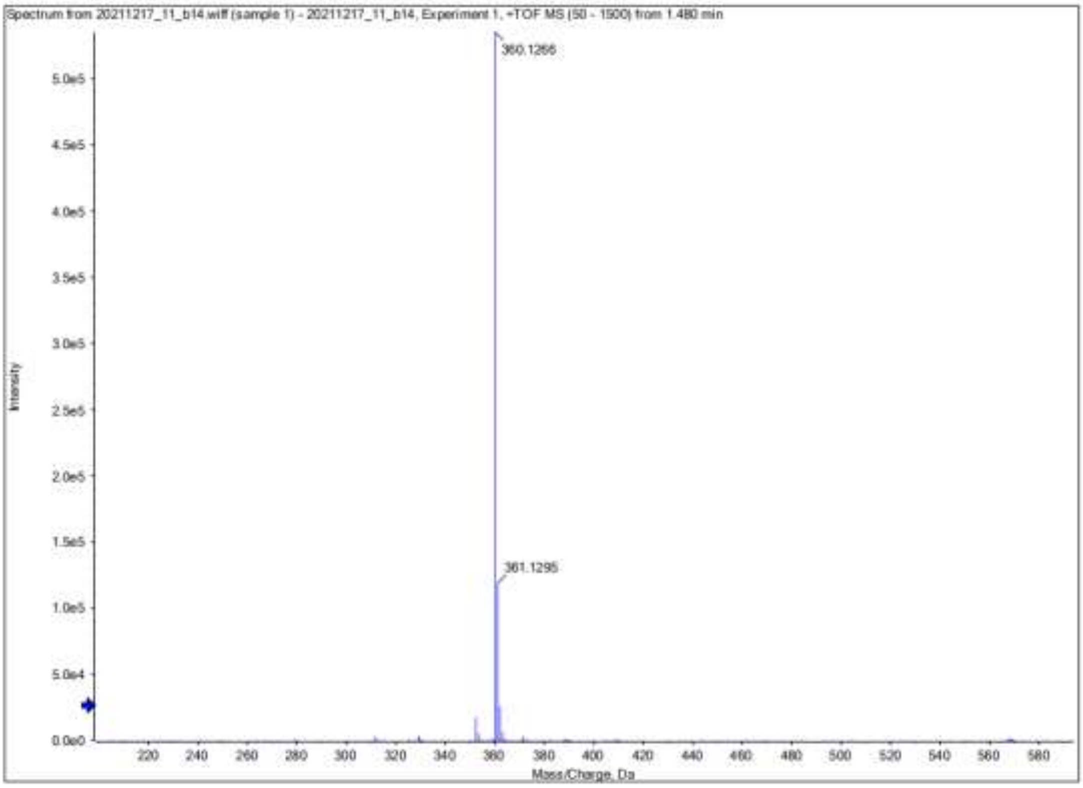

Spartinin C5

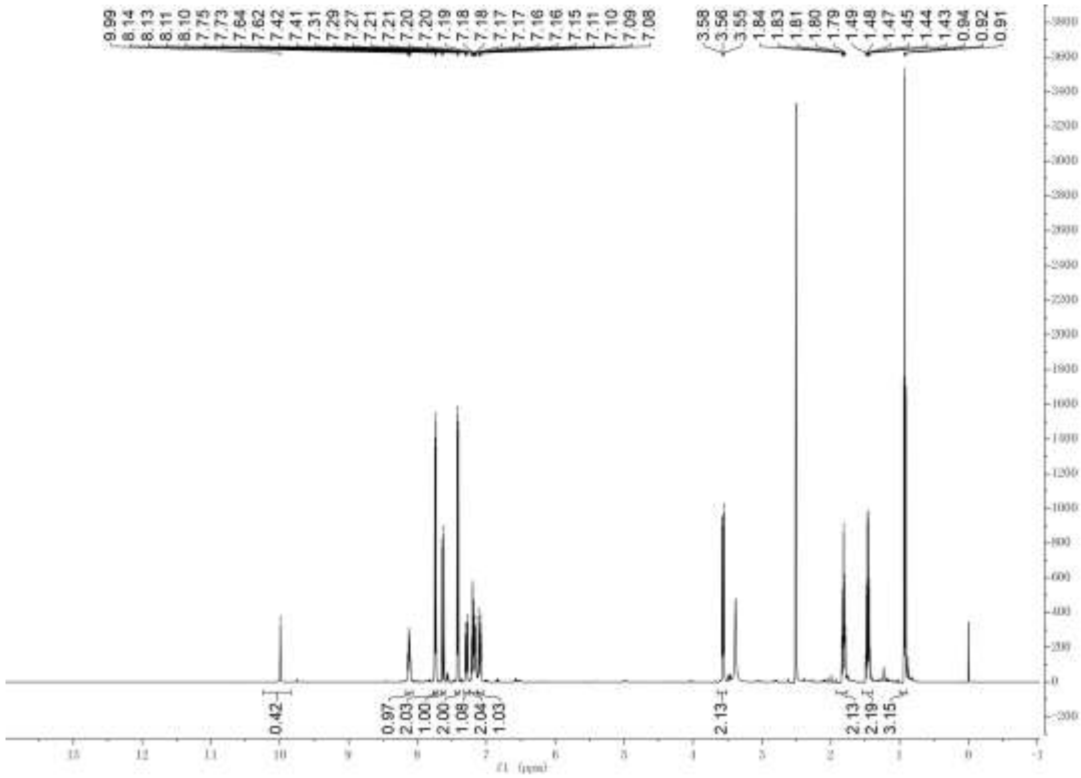
9.99 8.14 8.13 8.11 8.10 7.75 7.73 7.64 7.62 7.42 7.41 7.31 7.29 7.27 7.21 7.20 7.20 7.19 7.18 7.18 7.17 7.17 7.16 7.16 7.15 7.11 7.10 7.09 7.08
3.58 3.56 3.55 1.84 1.83 1.81 1.80 1.79 1.49 1.48 1.47 1.45 1.44 1.43 0.94 0.92 0.91
0.42
0.97 2.03 1.00 2.00 1.08 2.04 1.03
2.13
2.13 2.19 3.15
f1 (ppm)

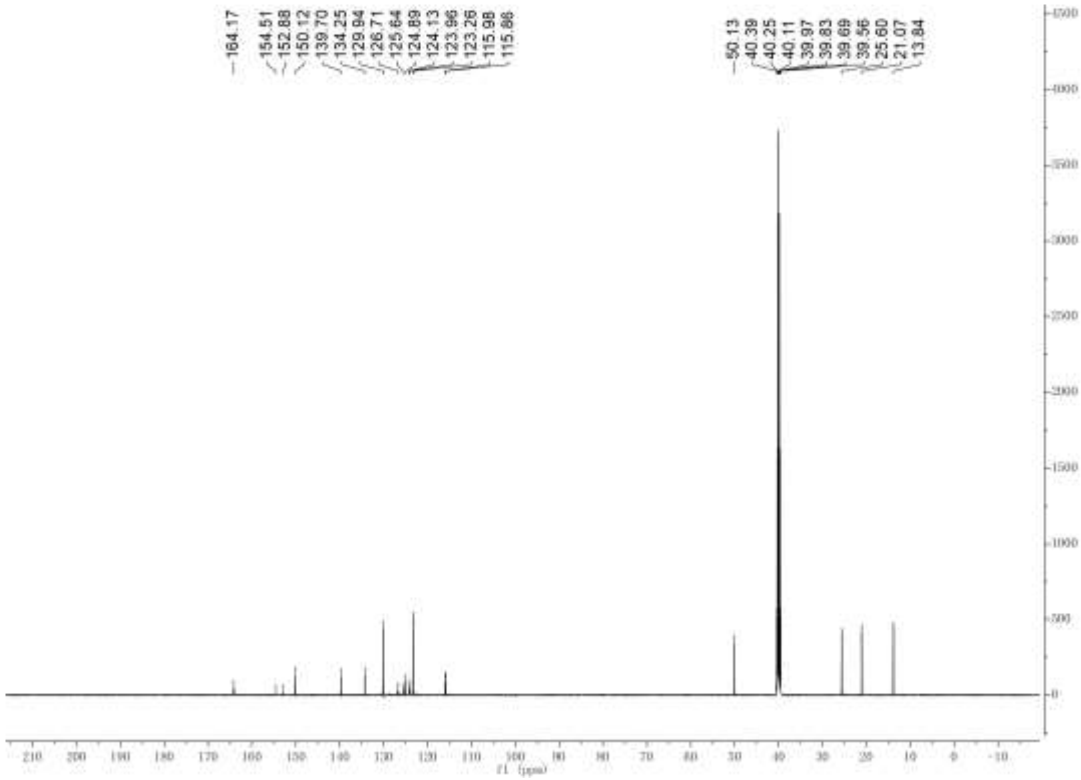
164.17
154.51 152.88 150.12 139.70 134.25 129.94 126.71 125.64 124.89 124.13 123.96 123.26 115.98 115.88
50.13 40.39 40.25 40.11 39.97 39.83 39.69 39.56 25.60 21.07 13.84
f1 (ppm)

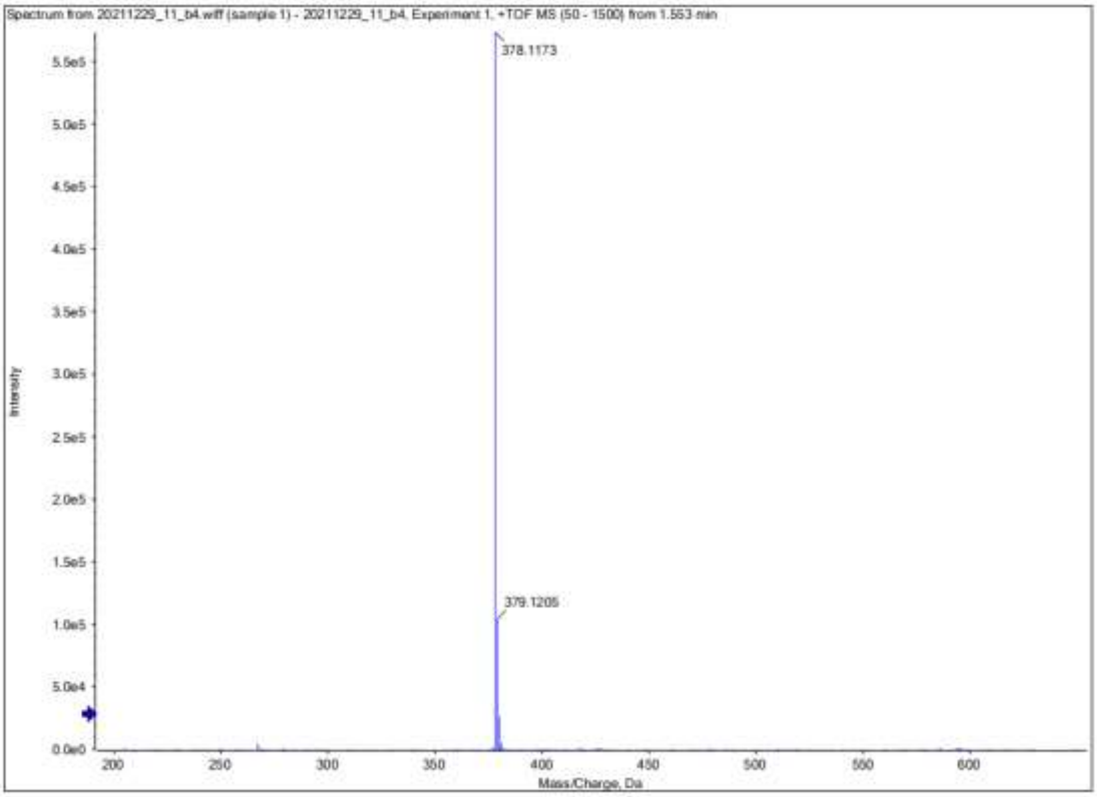

Spartinin C6

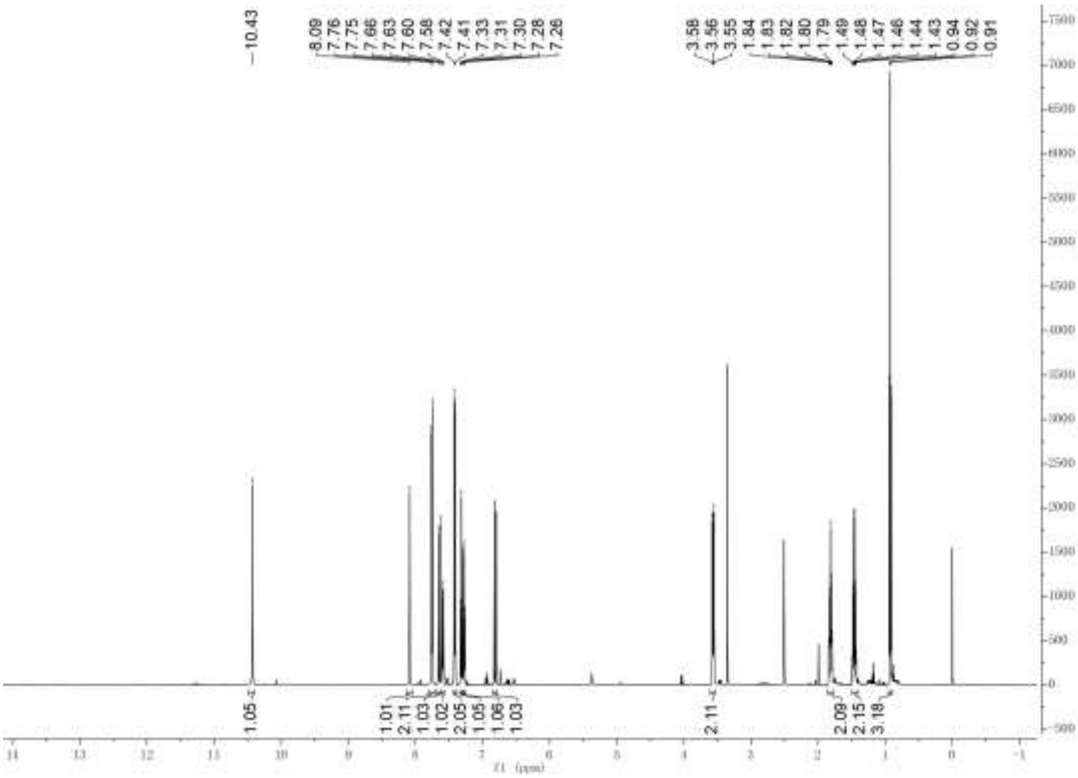

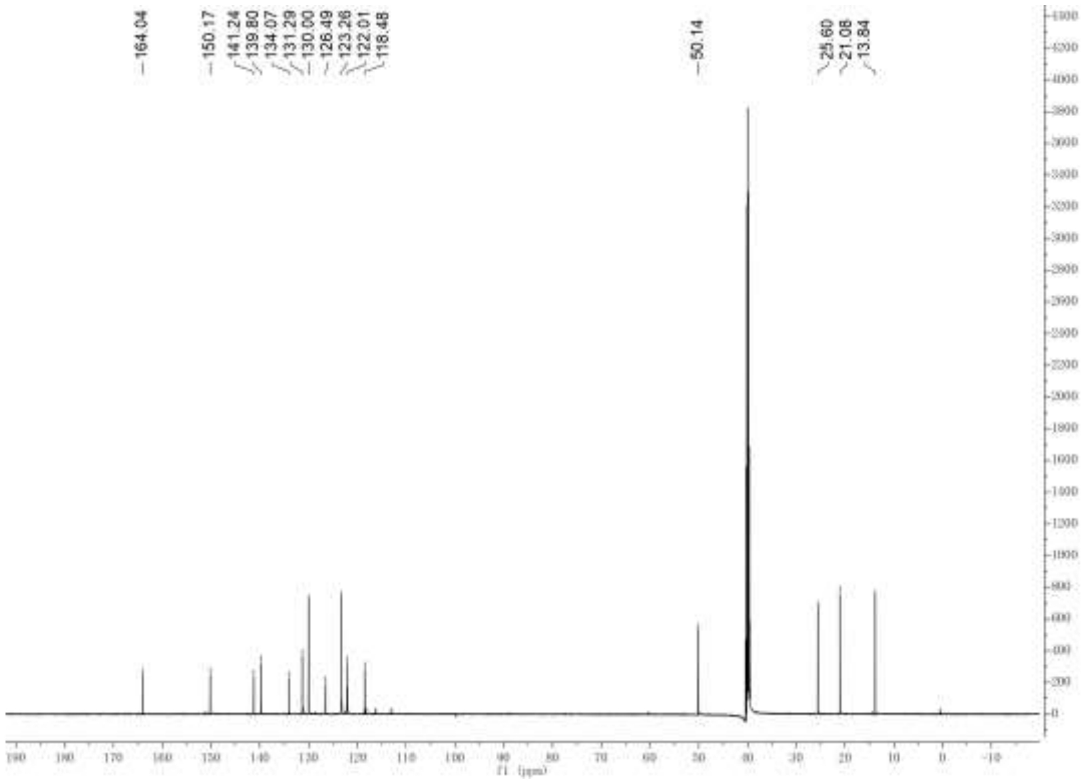

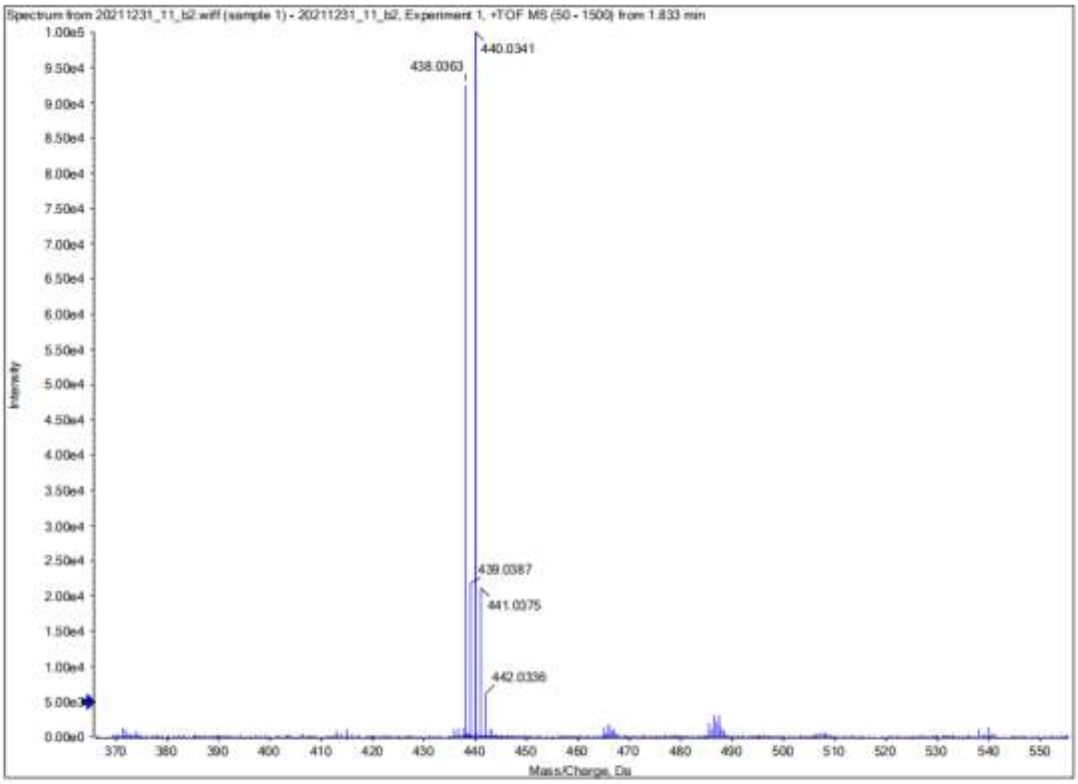

Spartinin C7

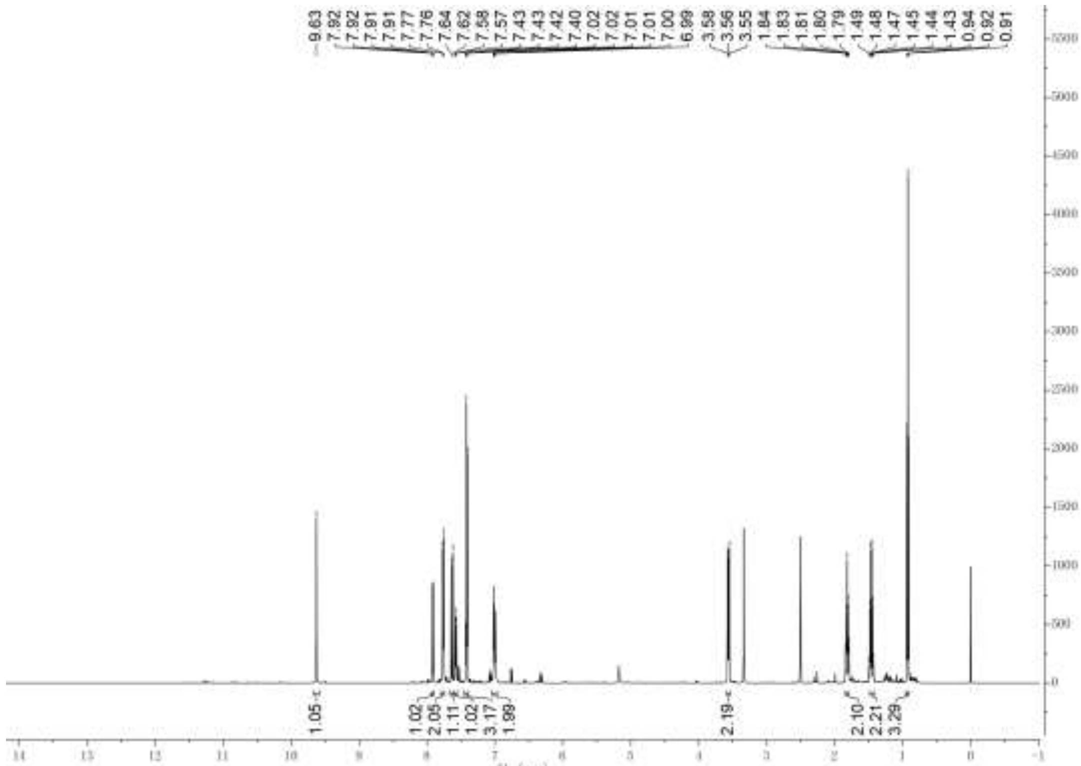
9.63
7.92
7.92
7.91
7.91
7.77
7.76
7.64
7.62
7.58
7.57
7.43
7.43
7.42
7.40
7.02
7.02
7.01
7.01
7.00
6.99
3.58
3.56
3.55
1.84
1.83
1.81
1.80
1.79
1.49
1.48
1.47
1.45
1.44
1.43
0.94
0.92
0.91
1.05
1.02
2.05
1.11
1.02
3.17
1.99
2.19
2.10
2.21
3.29
f1 (ppm)

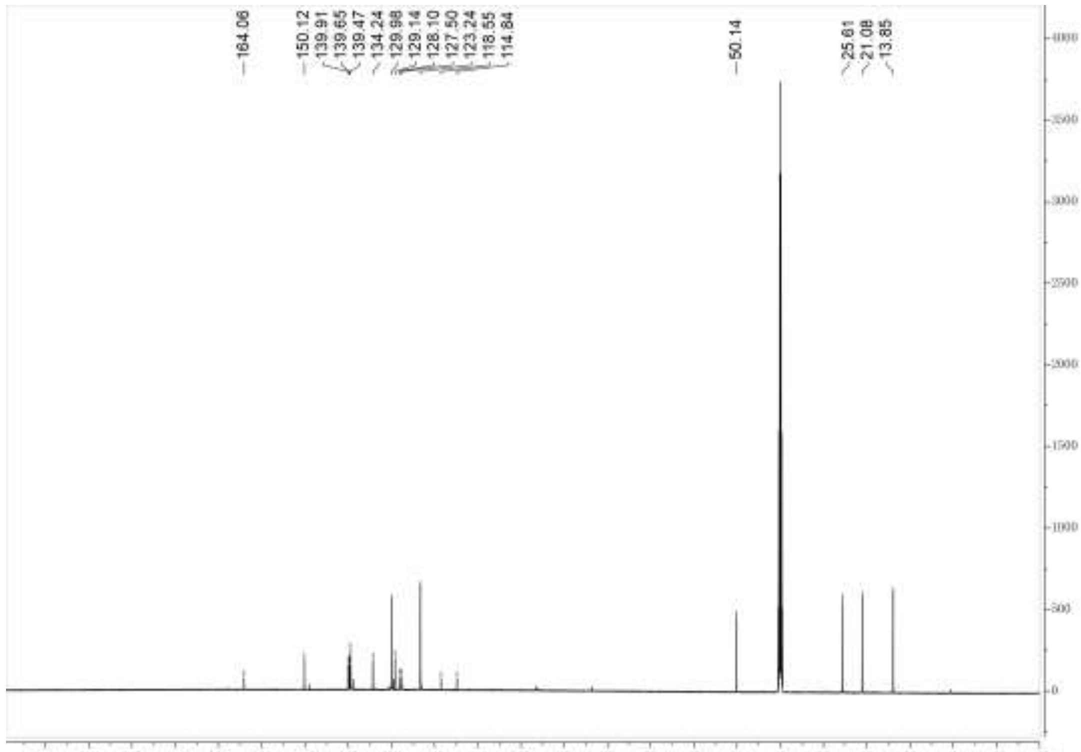
164.06
150.12
139.91
139.65
139.47
134.24
129.98
129.14
128.10
127.50
123.24
118.55
114.84
50.14
25.61
21.08
13.85
f1 (ppm)

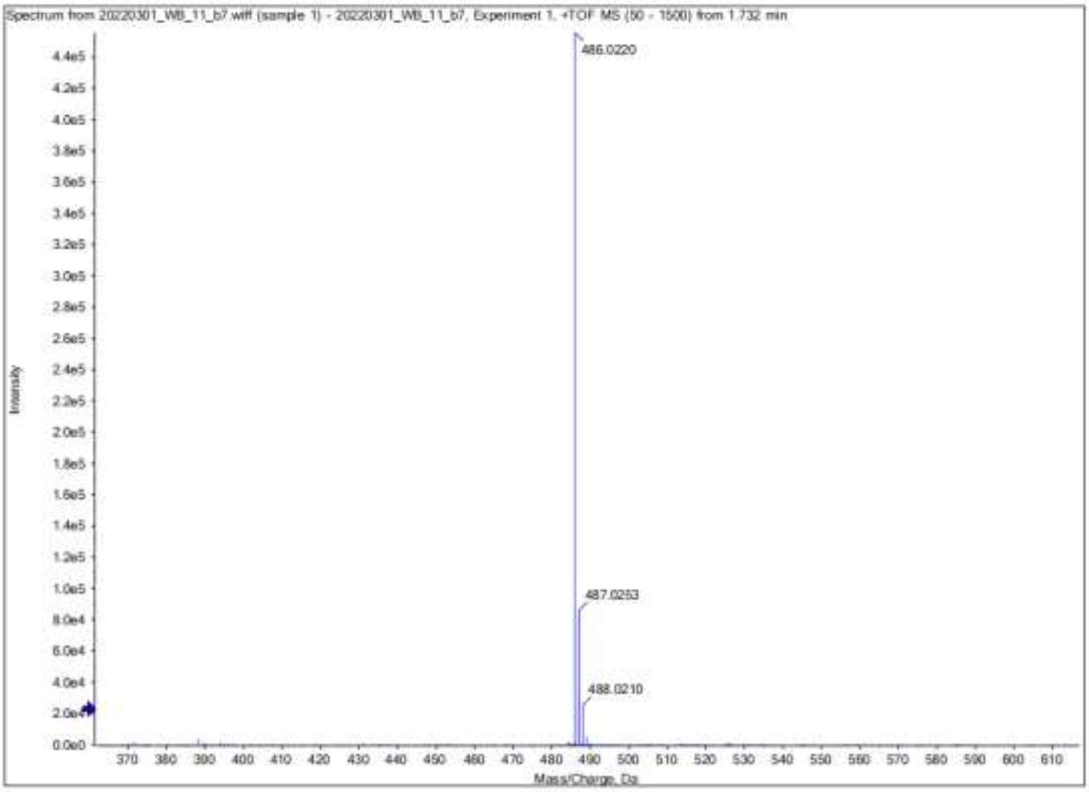

Spartinin C8

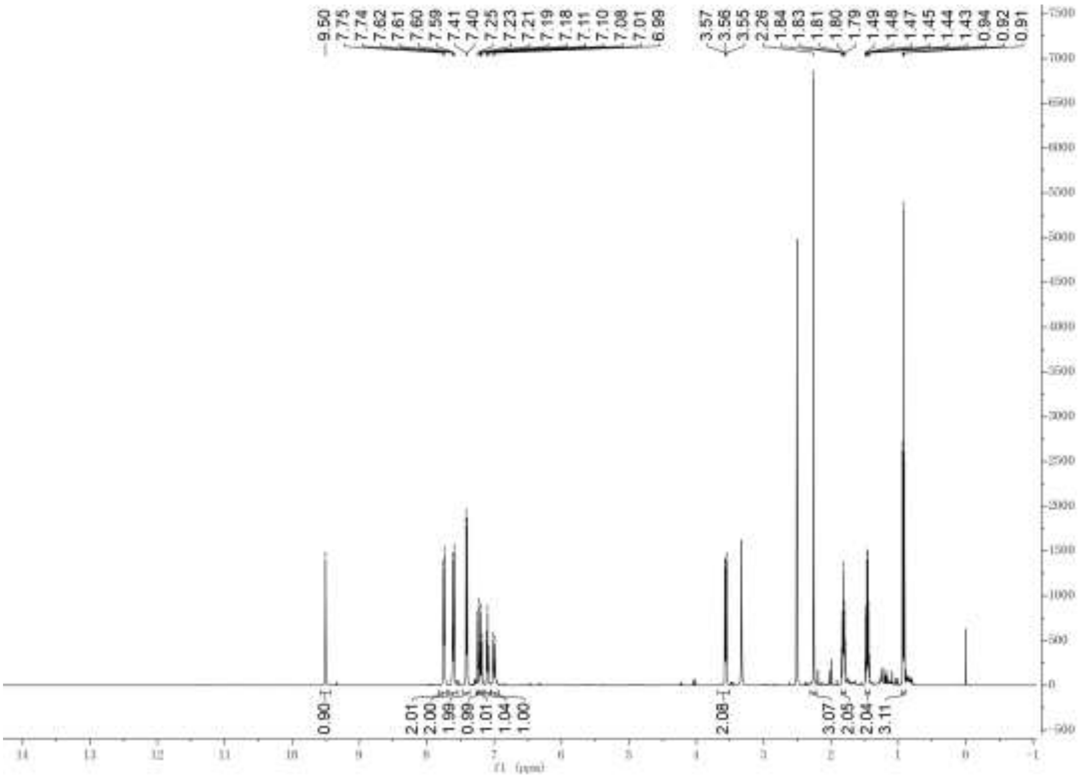

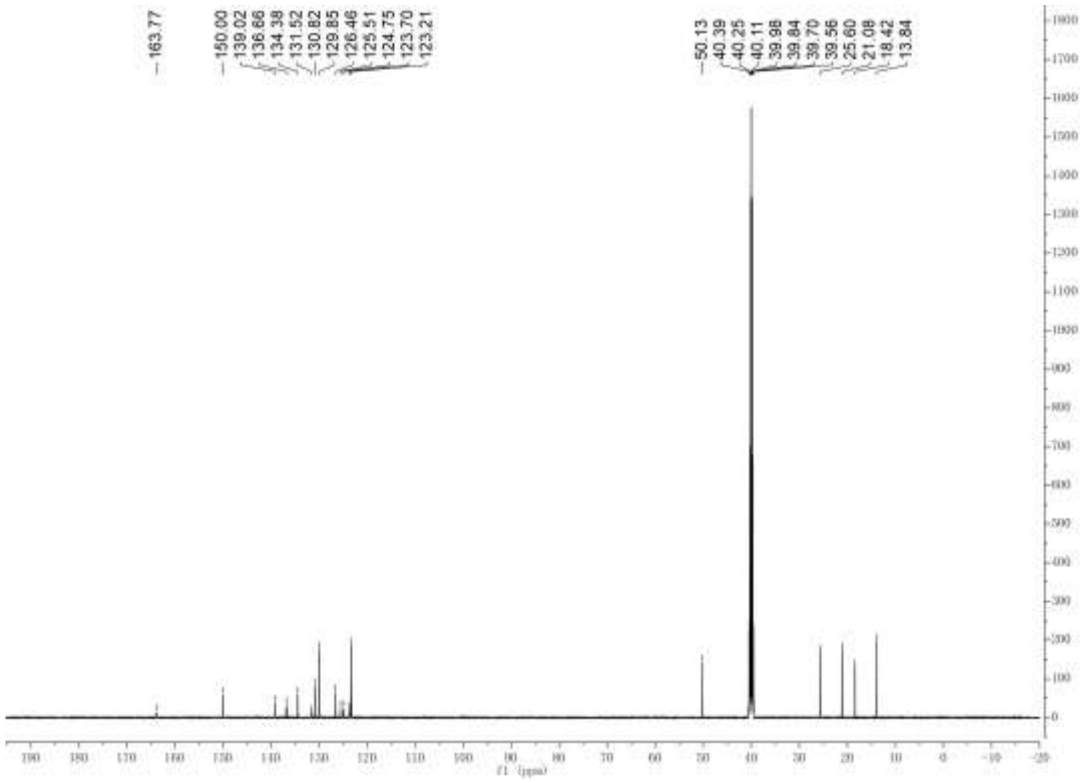

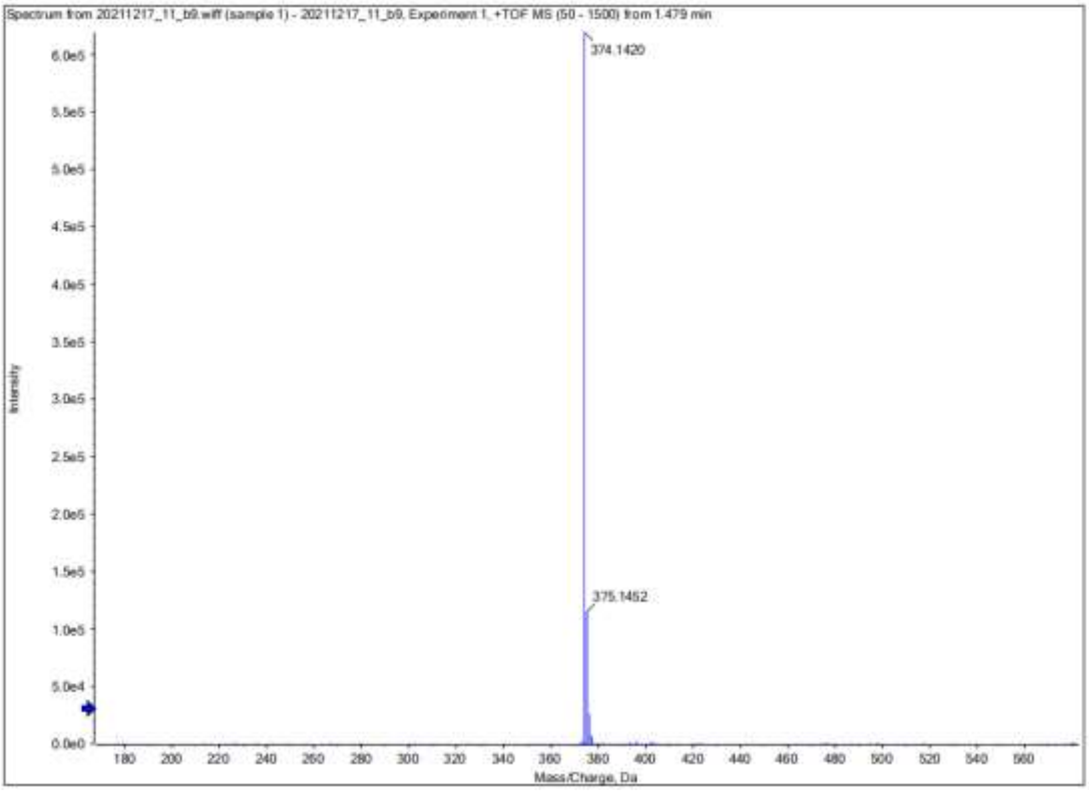

Spartinin C9

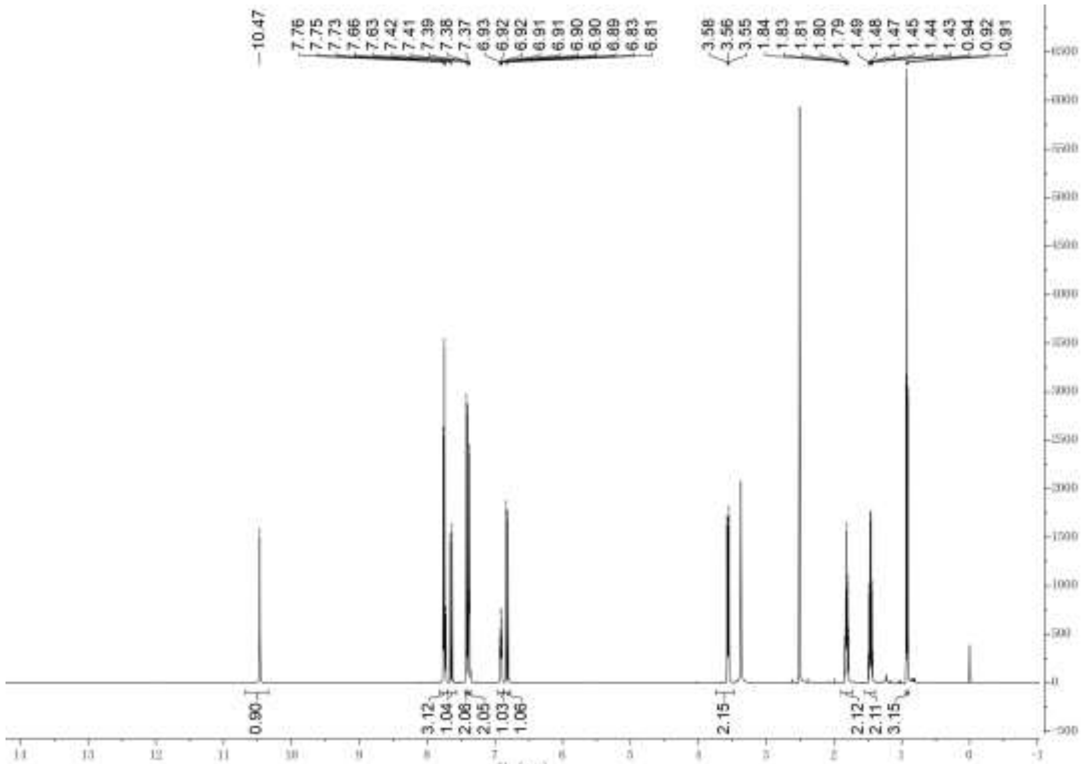
10.47
7.76 7.75 7.73 7.66 7.63 7.42 7.41 7.39 7.38 7.37 6.93 6.92 6.92 6.91 6.91 6.90 6.90 6.89 6.83 6.81
3.58 3.56 3.55 1.84 1.83 1.81 1.80 1.79 1.49 1.48 1.47 1.45 1.44 1.43 0.94 0.92 0.91
0.90 3.12 1.04 2.08 2.05 1.03 1.08 2.15 2.12 2.11 3.15
f1 (ppm)

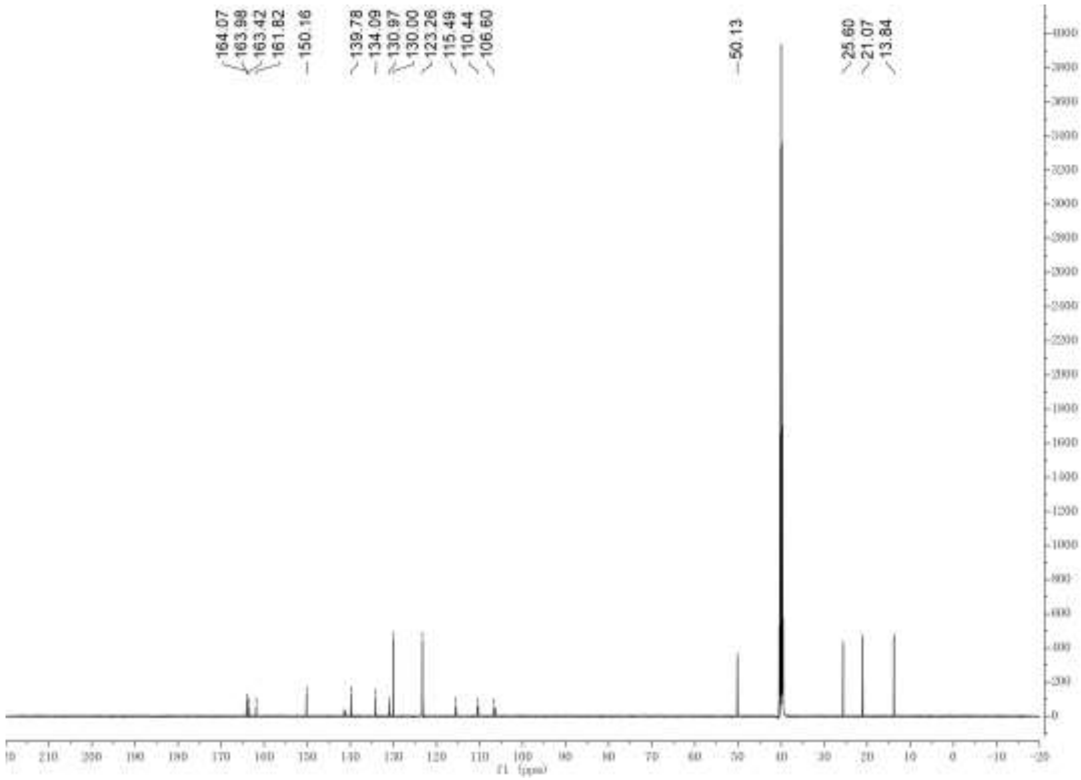
164.07 163.98 163.42 161.82 150.16 139.78 134.09 130.97 130.00 123.26 115.49 110.44 106.60
50.13
25.60 21.07 13.84
f1 (ppm)

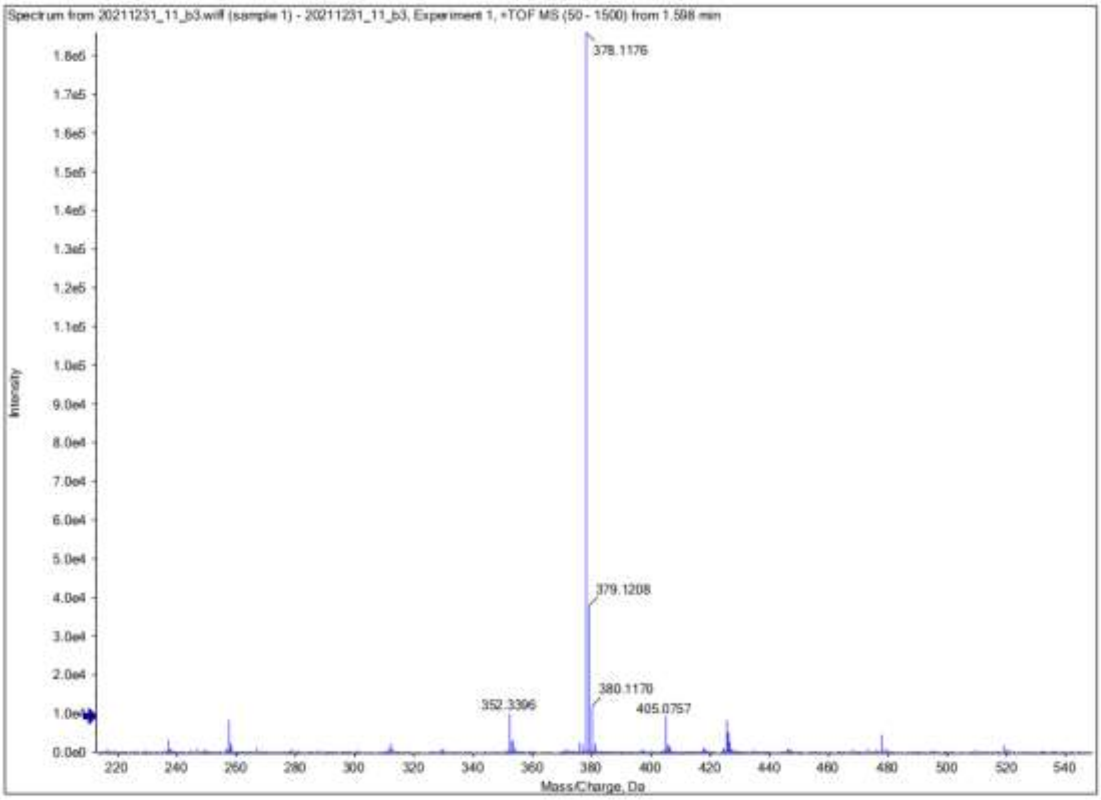

Spartinin C10

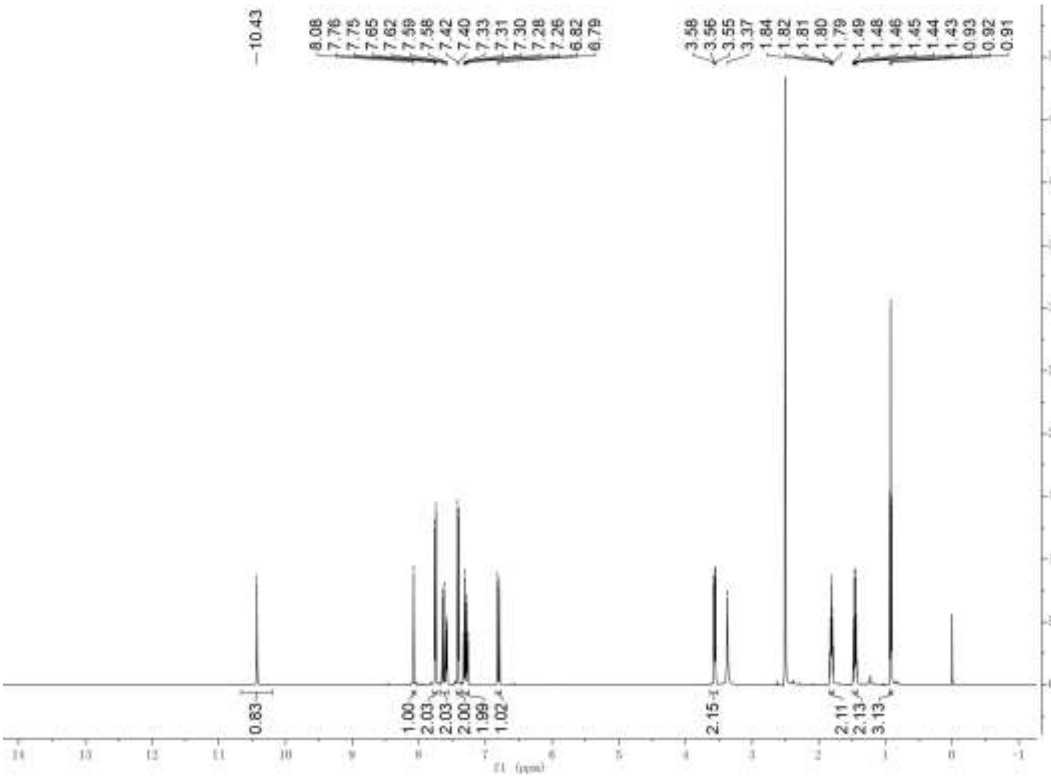

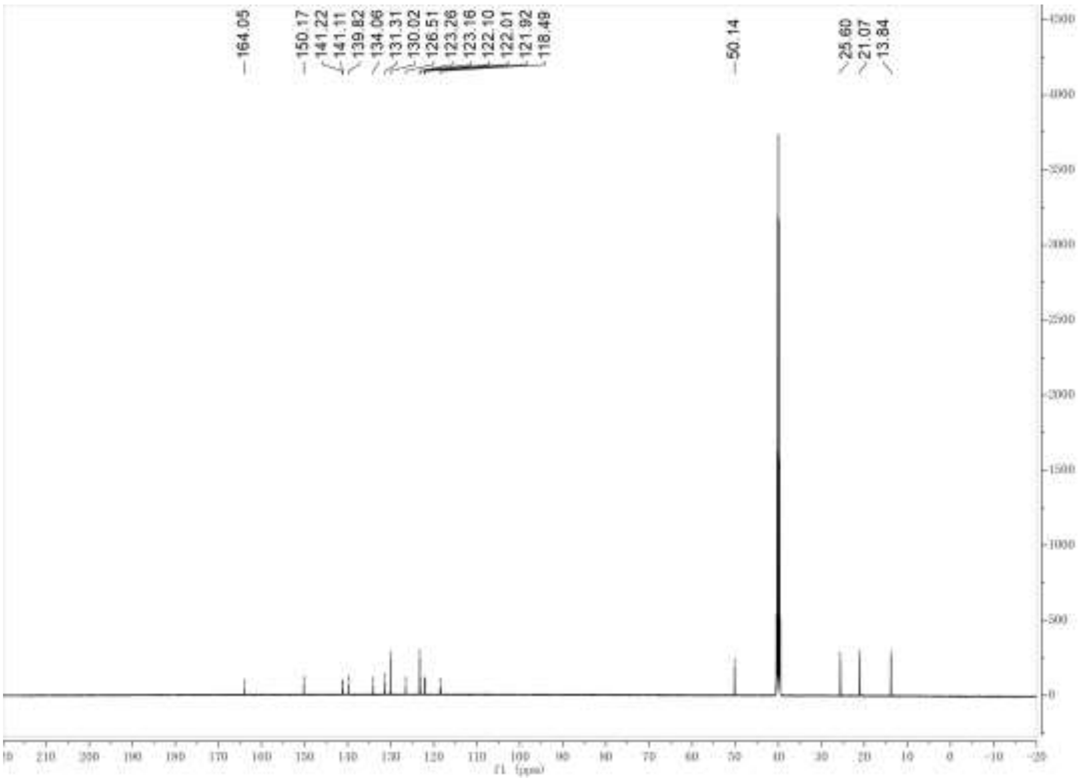

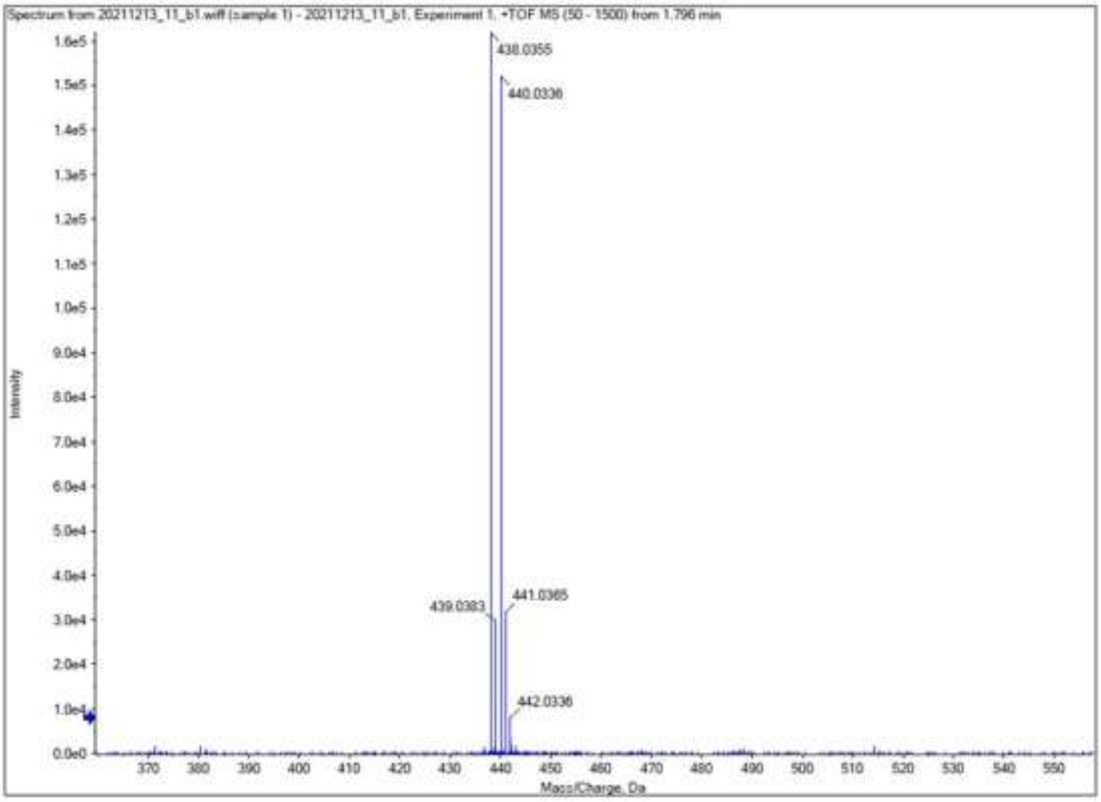

Spartinin C11

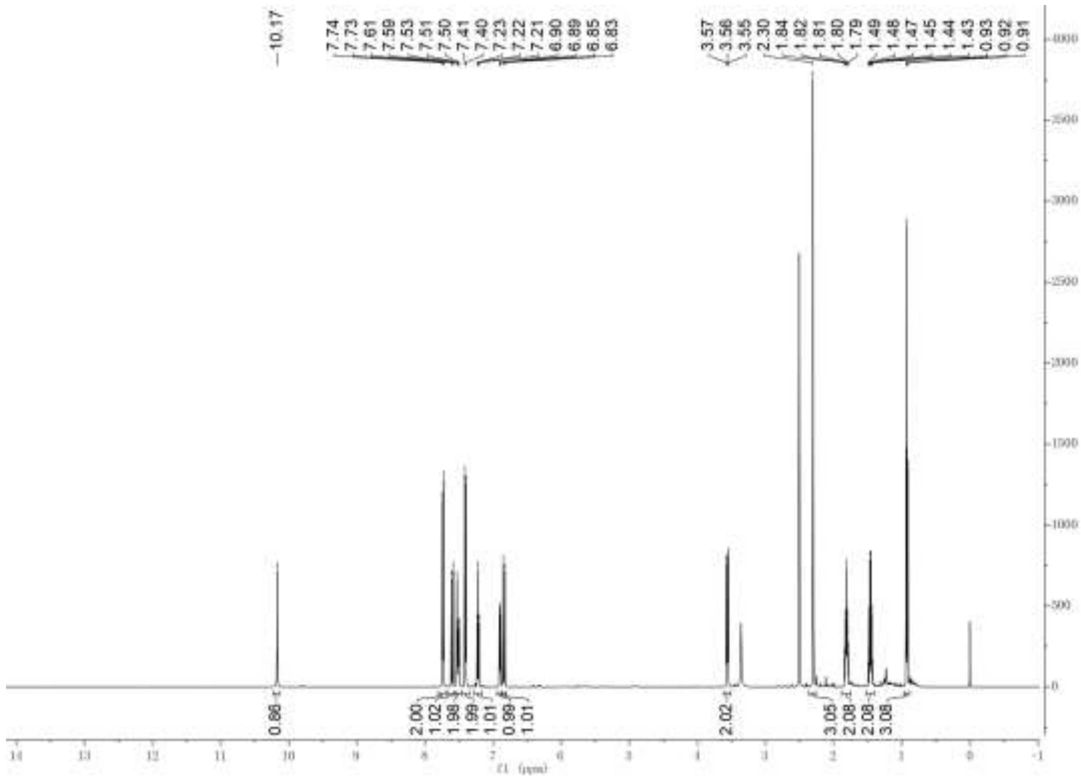
10.17
7.74
7.73
7.61
7.59
7.53
7.51
7.50
7.41
7.40
7.23
7.22
7.21
6.90
6.89
6.85
6.83
3.57
3.56
3.55
2.30
1.84
1.82
1.81
1.80
1.79
1.49
1.48
1.47
1.45
1.44
1.43
0.93
0.92
0.91
0.86
2.00
1.02
1.98
1.99
1.01
0.99
1.01
2.02
3.05
2.08
2.08
3.08
f1 (ppm)

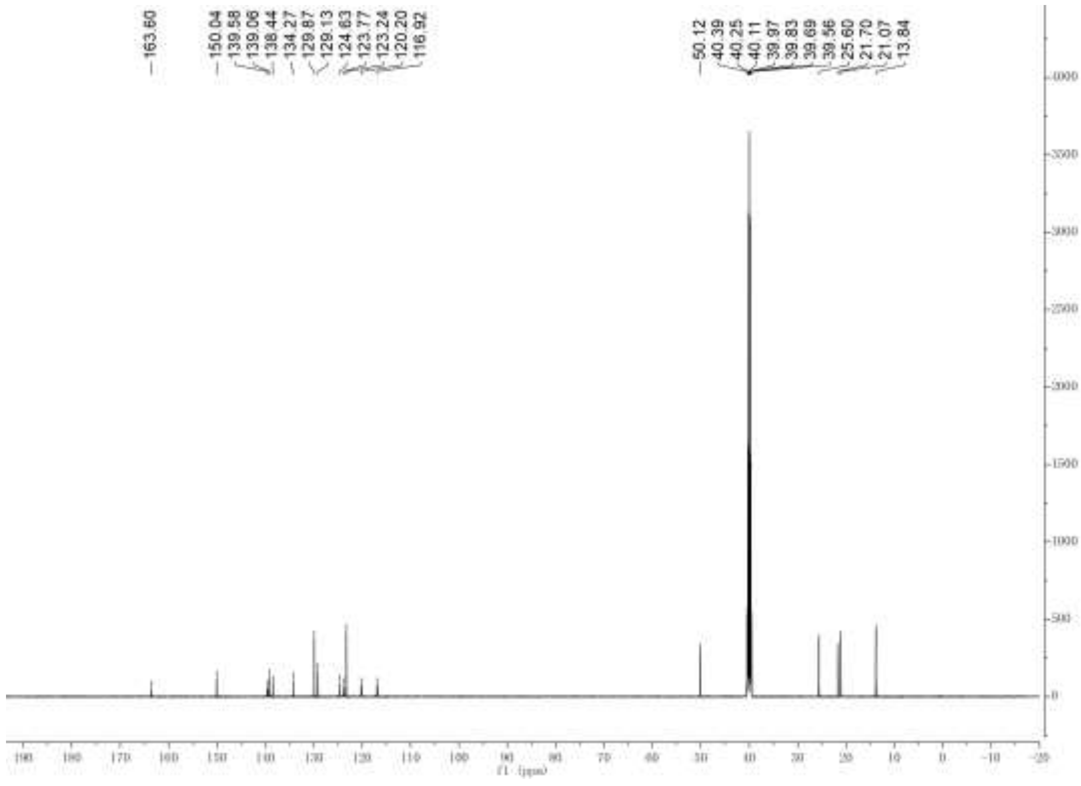
163.60
150.04
139.58
139.06
138.44
134.27
129.87
129.13
124.63
123.77
123.24
120.20
116.92
50.12
40.39
40.25
40.11
39.97
39.83
39.69
39.56
25.60
21.70
21.07
13.84
f1 (ppm)

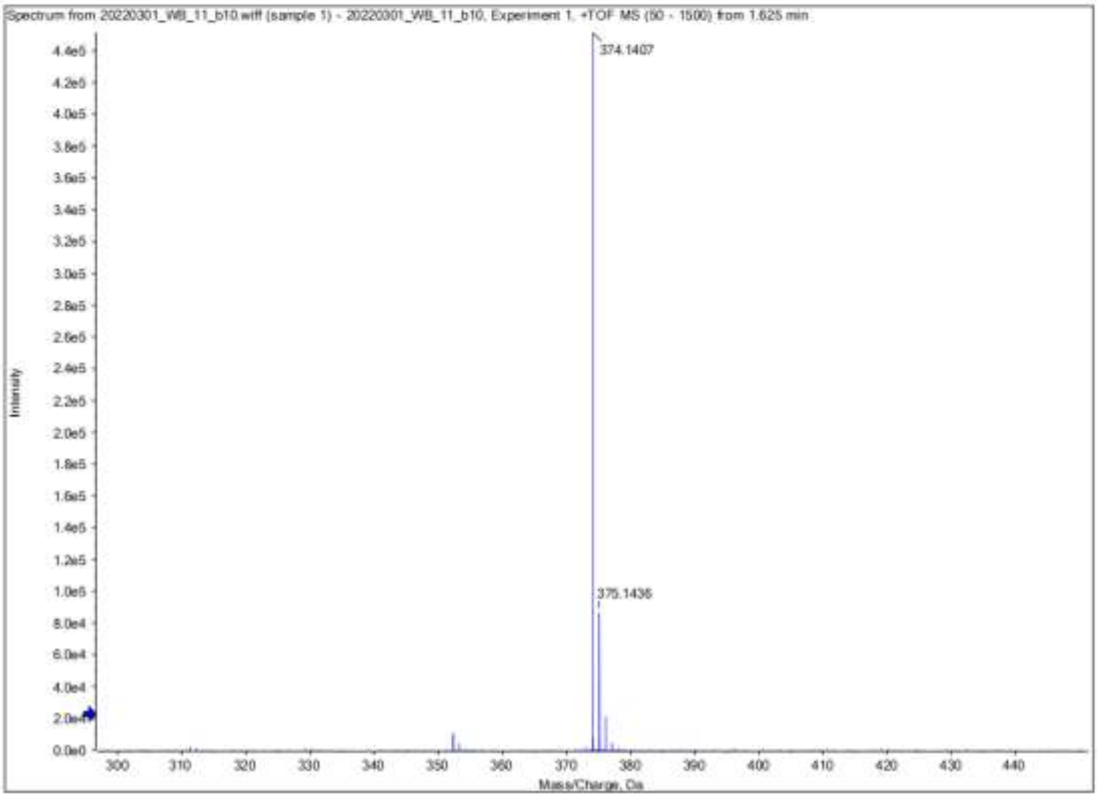

Spartinin C12

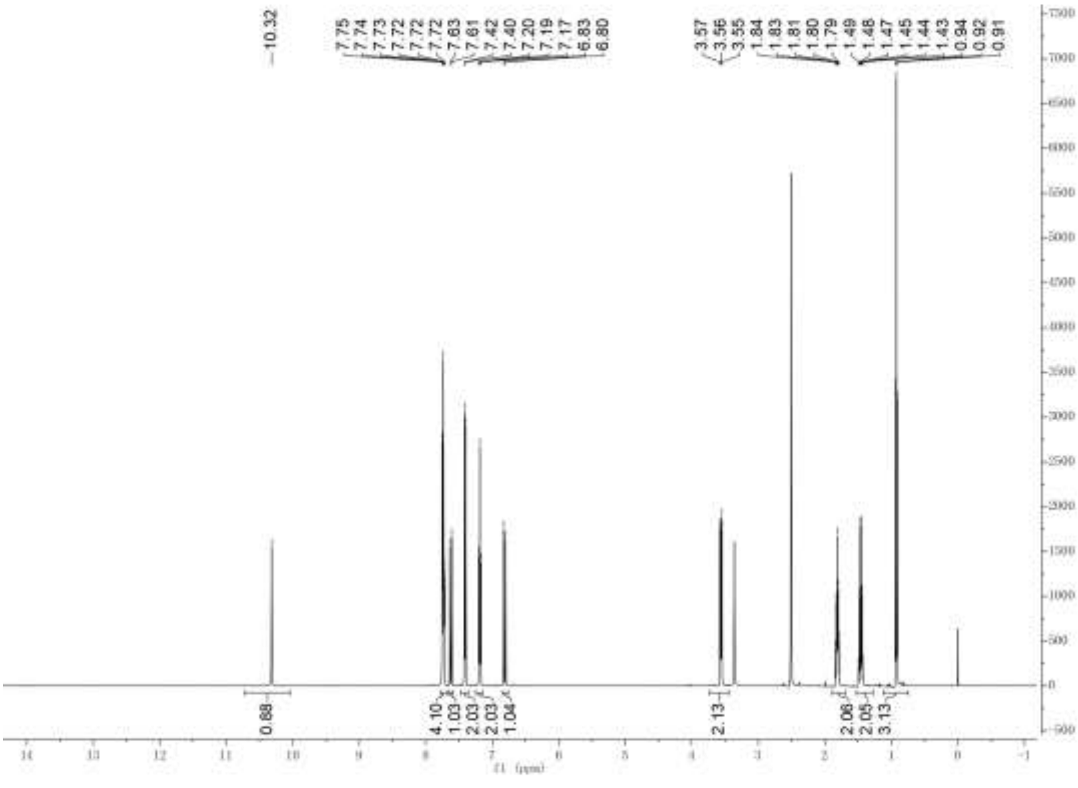

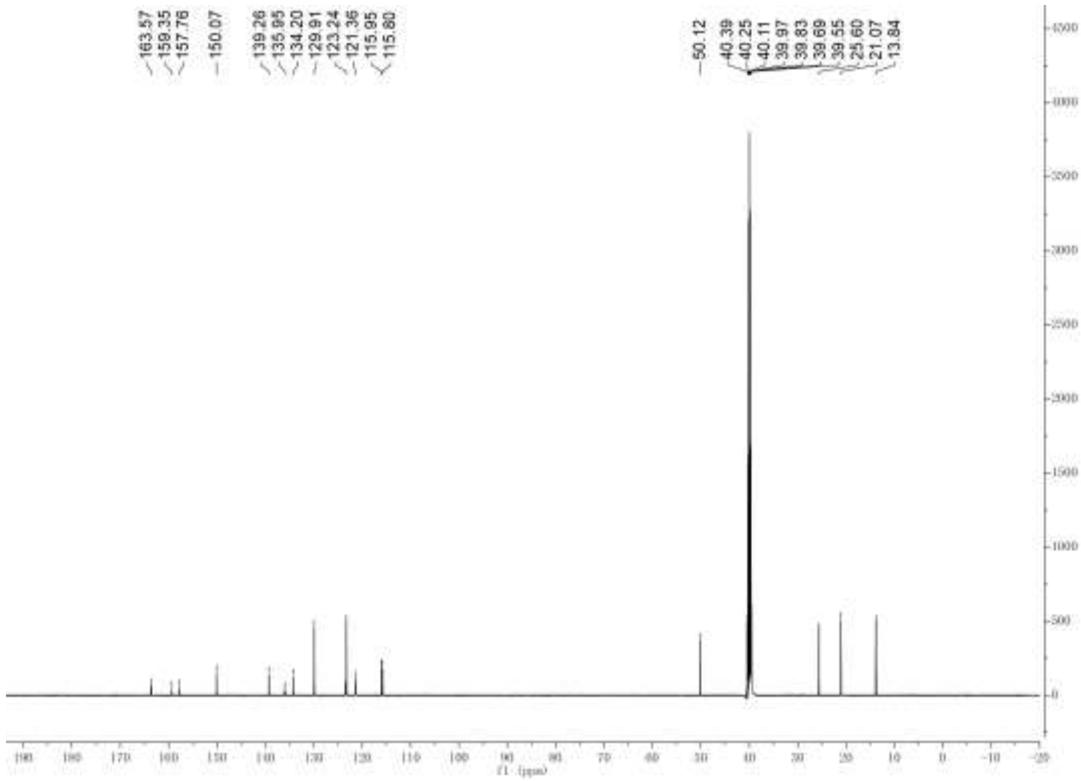

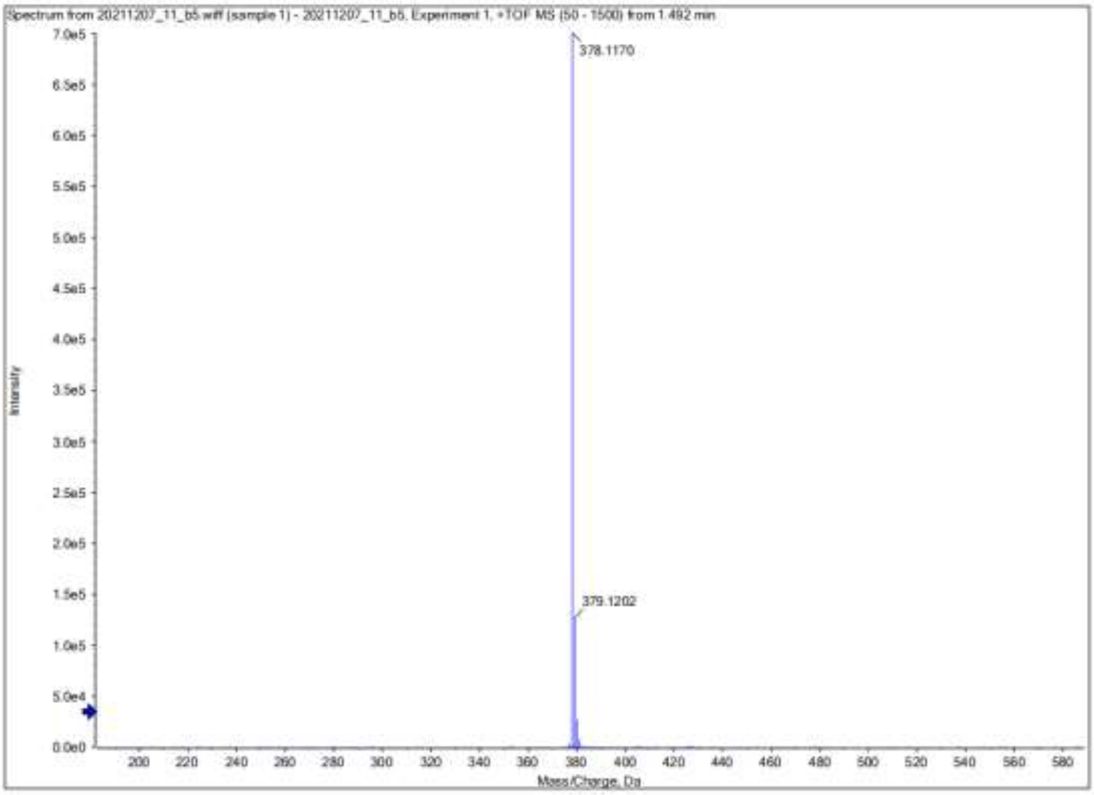

Spartinin C13

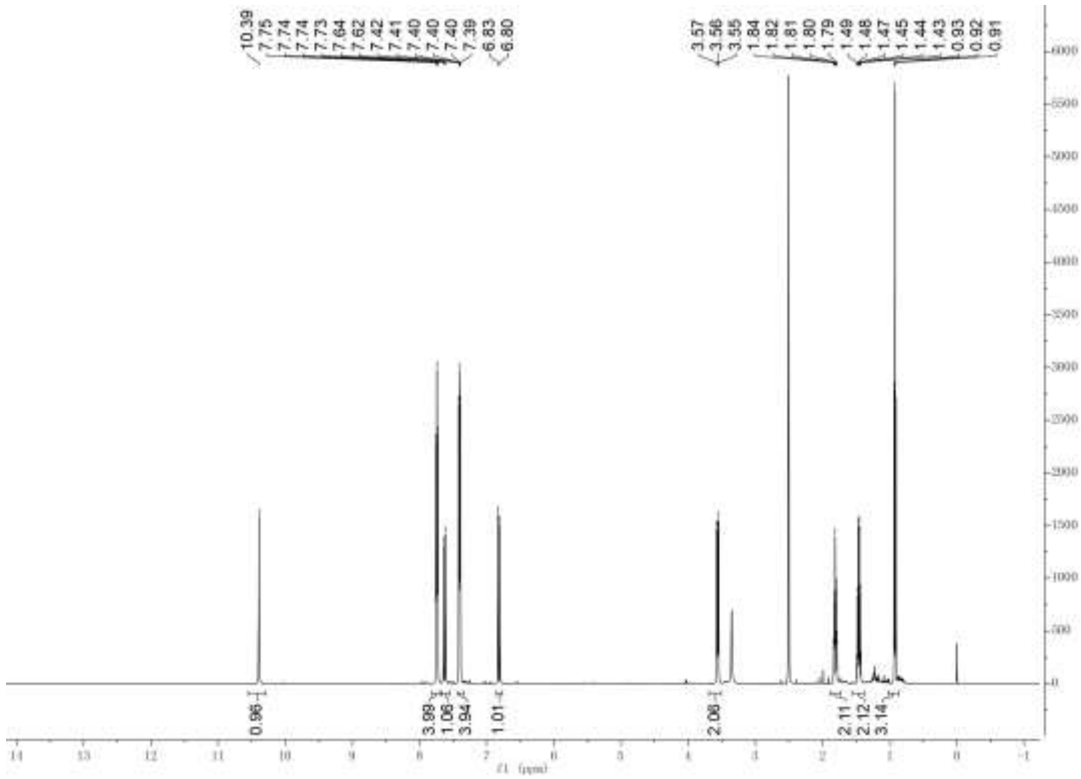
10.39
7.75
7.74
7.74
7.73
7.64
7.62
7.42
7.41
7.40
7.40
7.40
7.39
6.83
6.80
3.57
3.56
3.55
1.84
1.82
1.81
1.80
1.79
1.49
1.48
1.47
1.45
1.44
1.43
0.93
0.92
0.91
0.96
3.99
1.06
3.94
1.01
2.08
2.11
2.12
3.14
f1 (ppm)

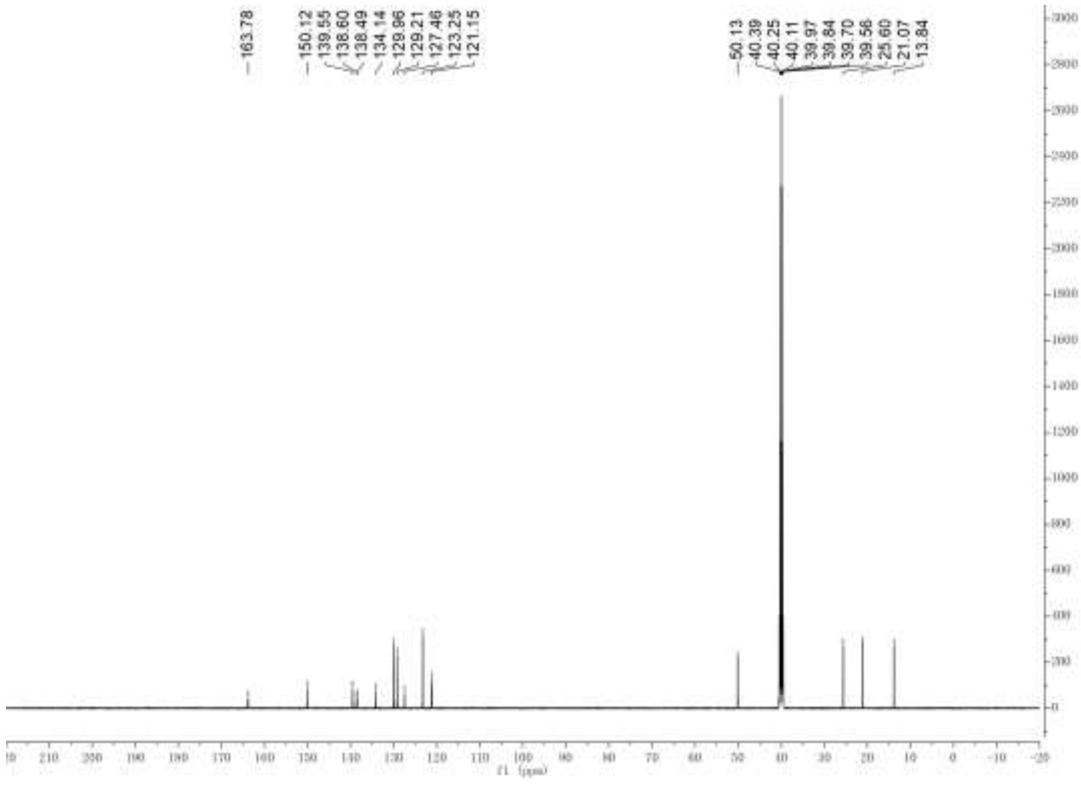
163.78
150.12
139.55
138.60
138.49
134.14
129.96
129.21
127.46
123.25
121.15
50.13
40.39
40.25
40.11
39.97
39.84
39.70
39.56
25.60
21.07
13.84
f1 (ppm)

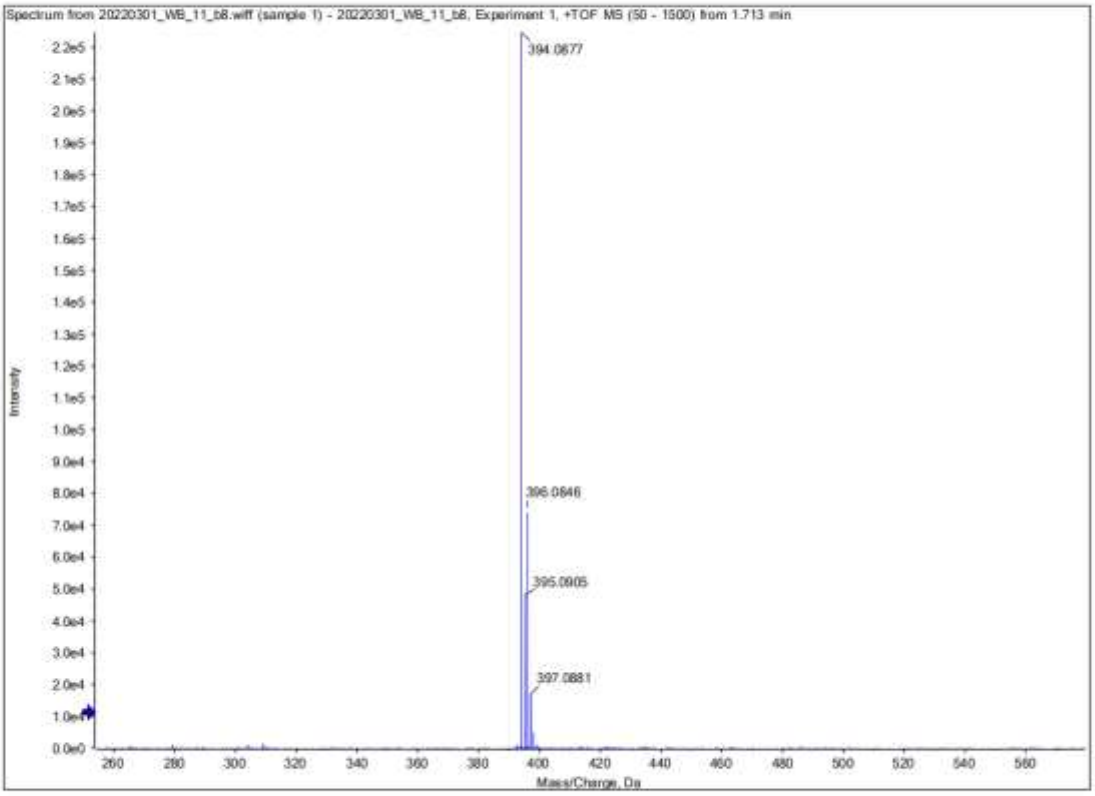

Spartinin C14

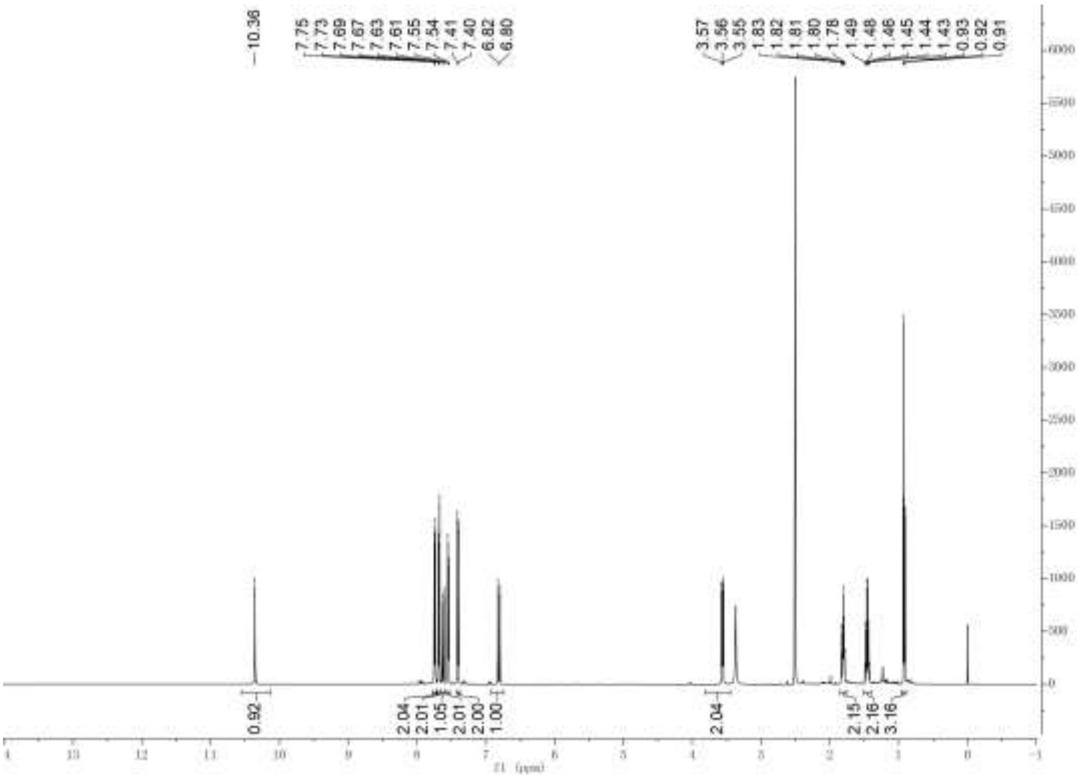

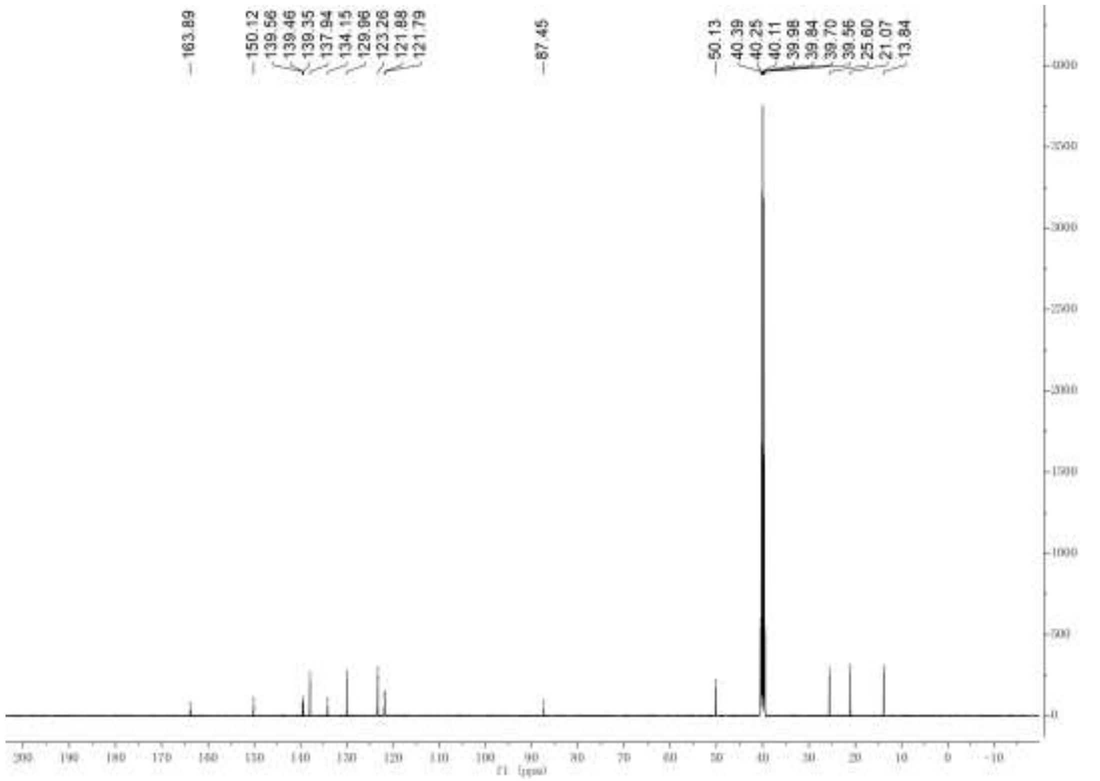

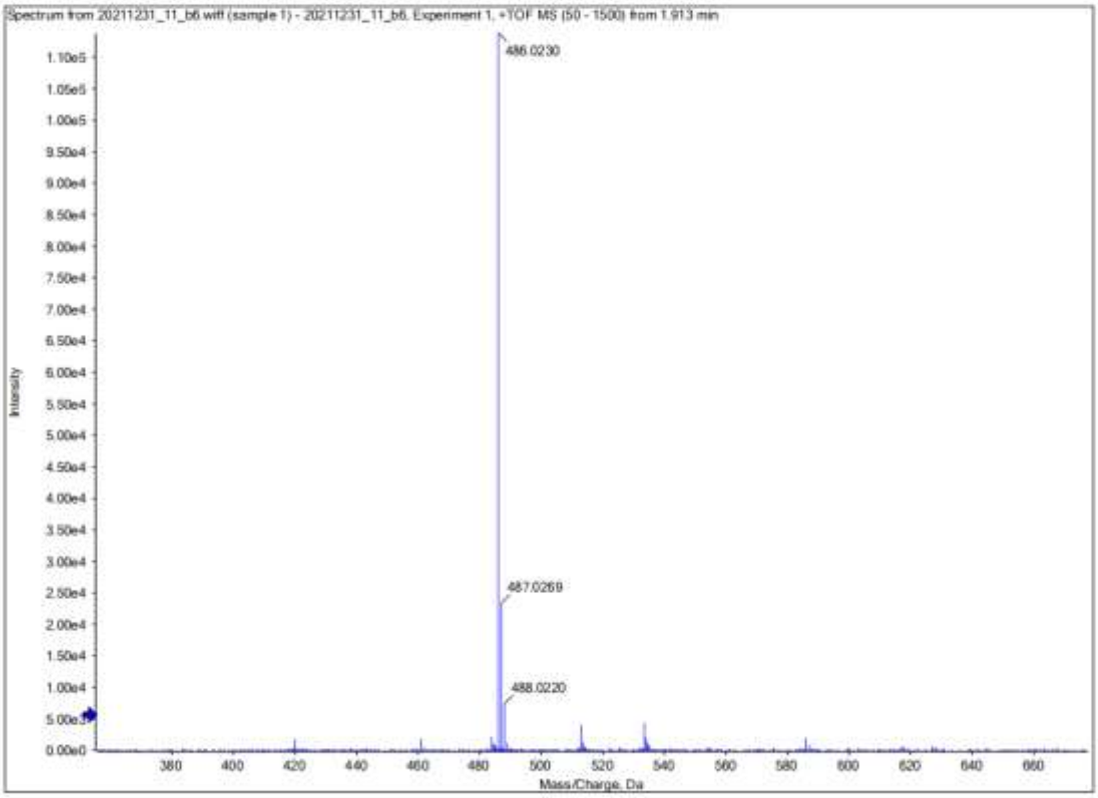

Spartinin C15

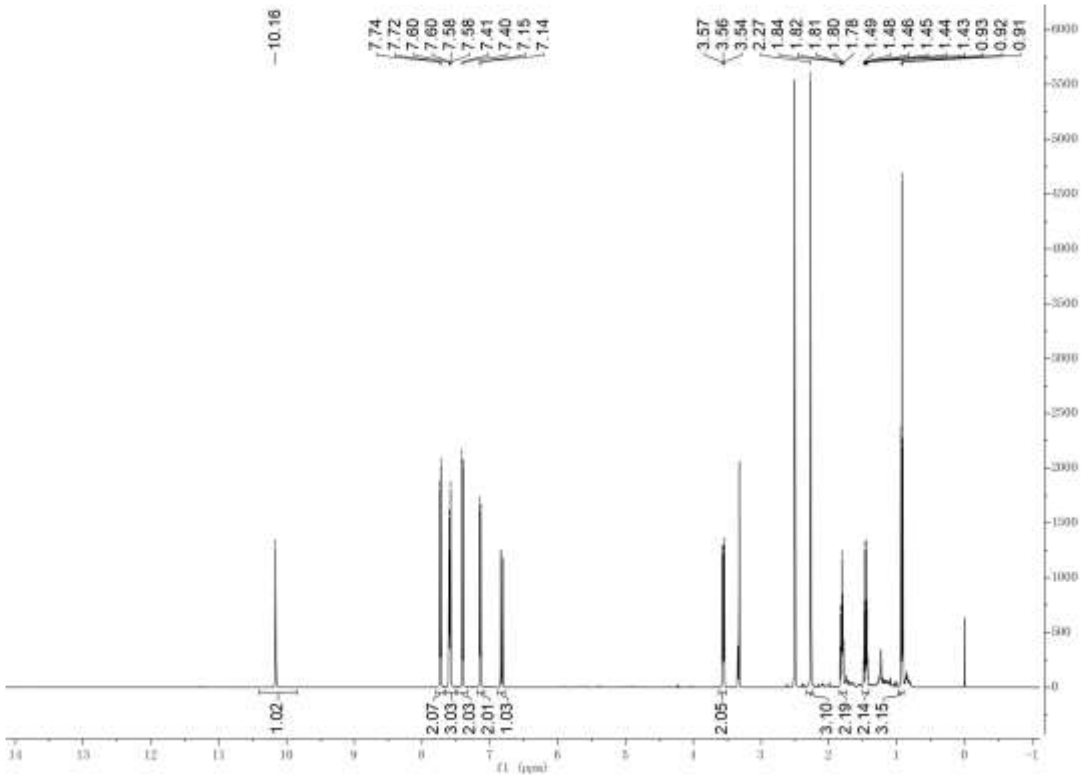
10.16
7.74 7.72 7.60 7.60 7.58 7.58 7.41 7.40 7.15 7.14
3.57 3.56 3.54 2.27 1.84 1.82 1.81 1.80 1.78 1.49 1.48 1.46 1.45 1.44 1.43 0.93 0.92 0.91
1.02
2.07 3.03 2.03 2.01 1.03
2.05
3.10 2.19 2.14 3.15
f1 (ppm)

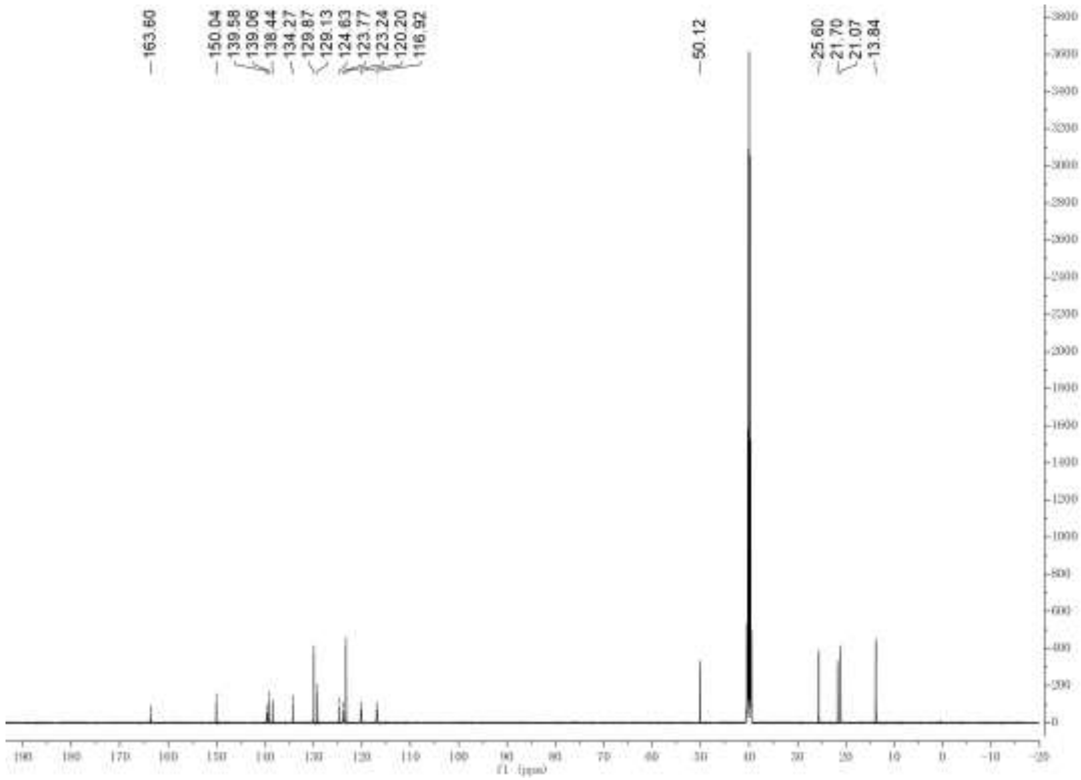
163.60
150.04 139.58 139.06 138.44 134.27 129.87 129.13 124.63 123.77 123.24 120.20 116.92
50.12
25.60 21.70 21.07 13.84
f1 (ppm)

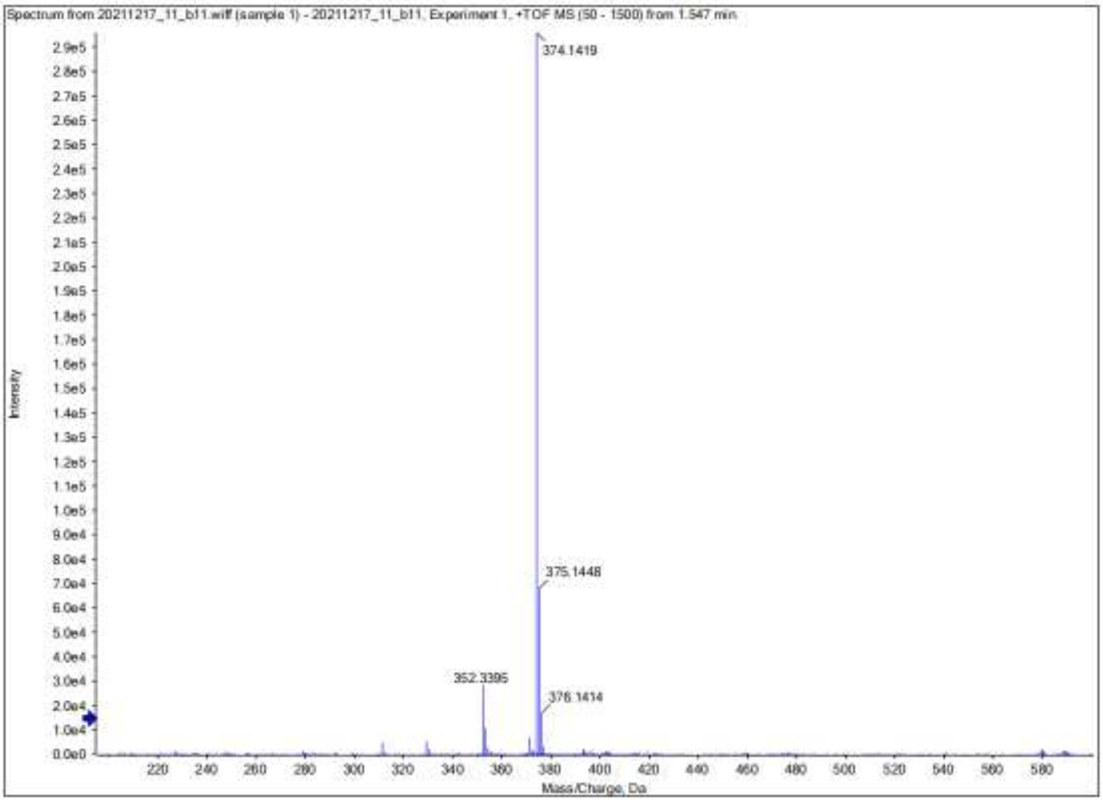

Spartinin C16

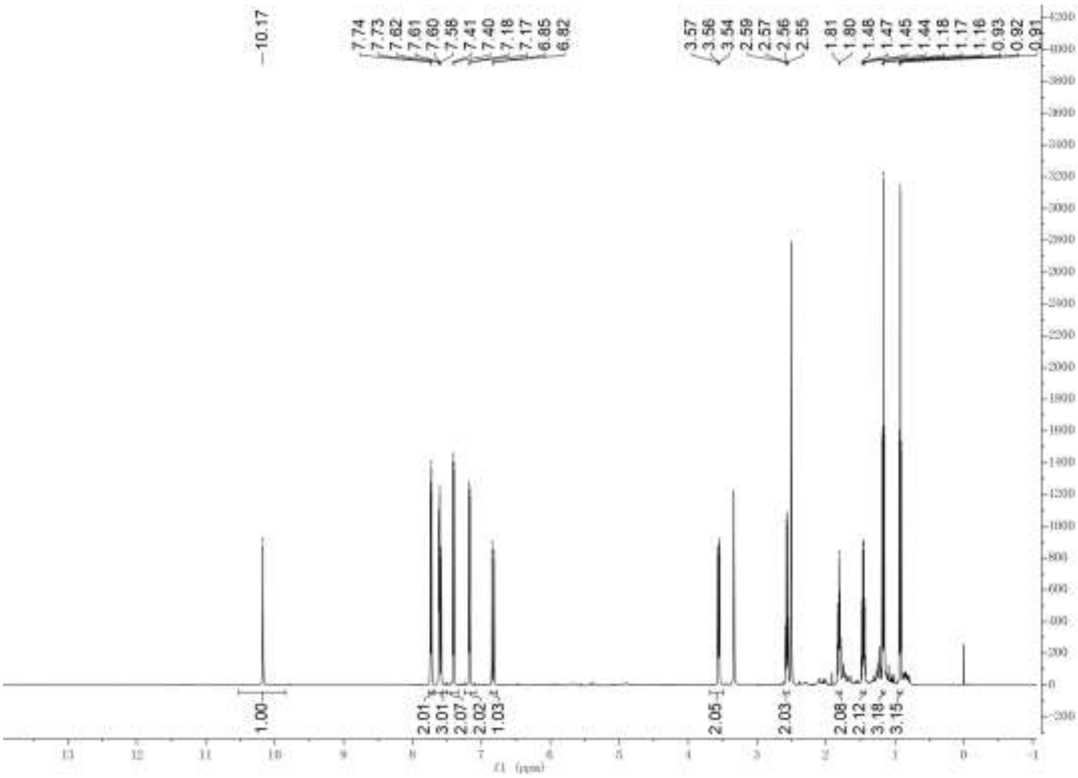

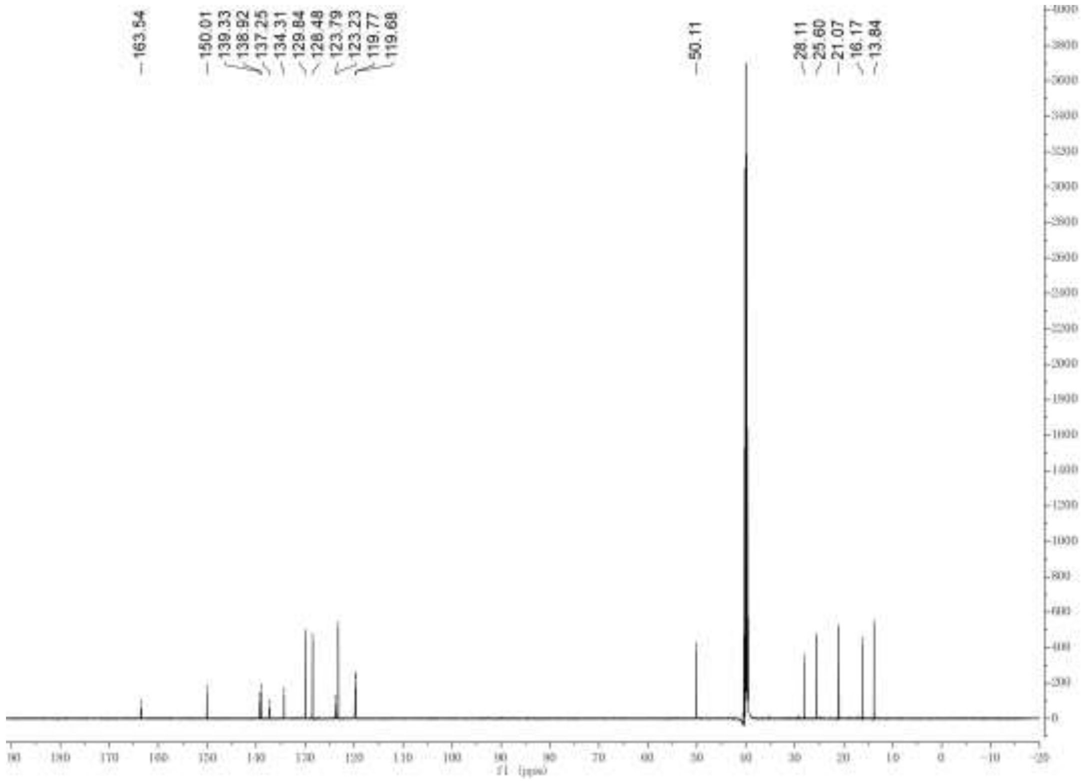

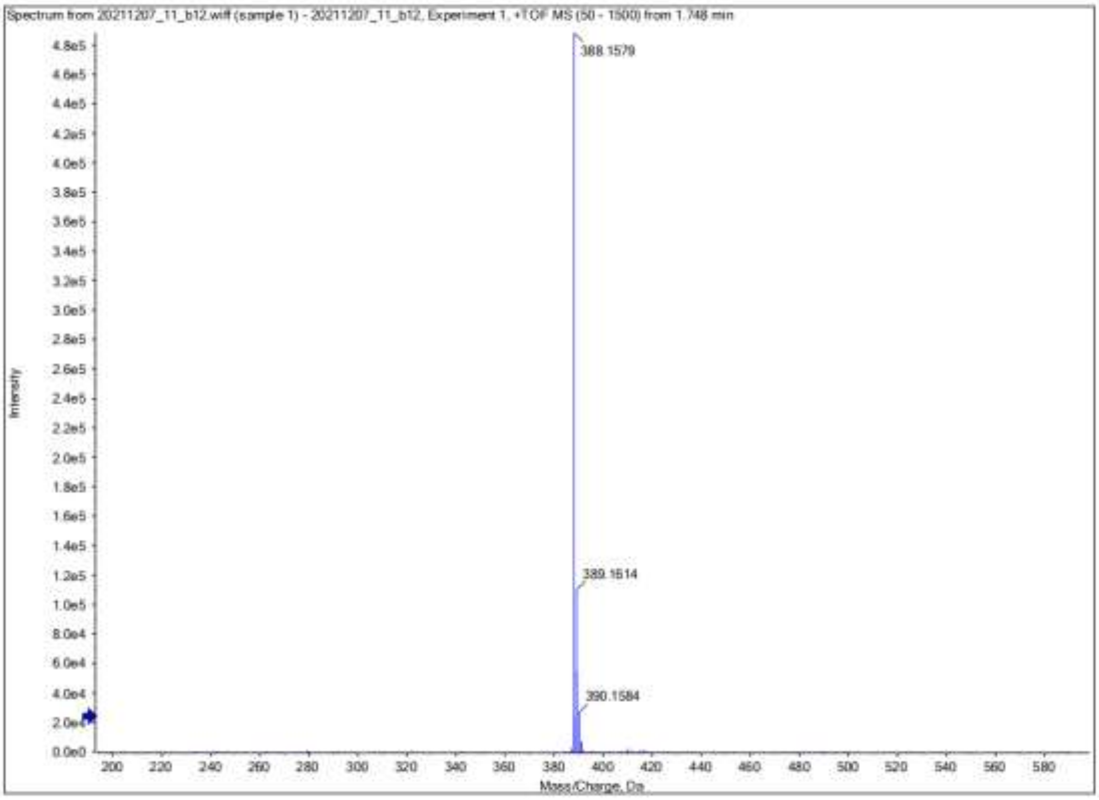

Spartinin C17

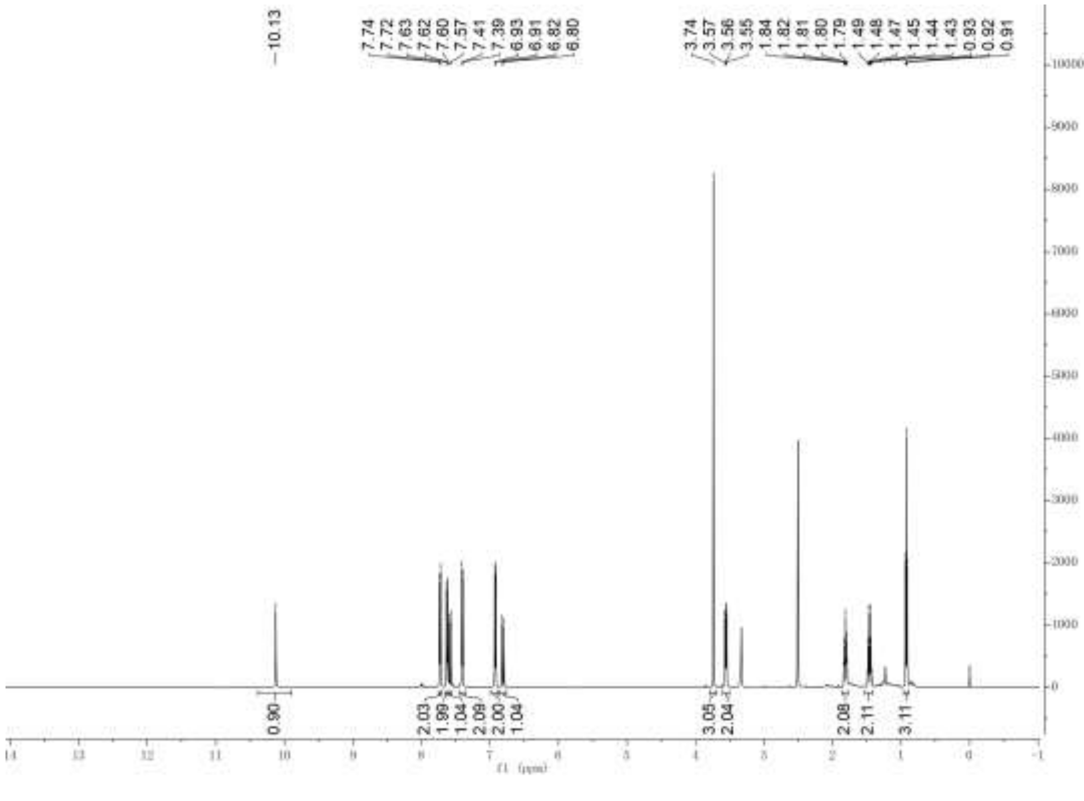
10.13
7.74
7.72
7.63
7.62
7.60
7.57
7.41
7.39
6.93
6.91
6.82
6.80
3.74
3.57
3.56
3.55
1.84
1.82
1.81
1.80
1.79
1.49
1.48
1.47
1.45
1.44
1.43
0.93
0.92
0.91
0.90
2.03
1.99
1.04
2.09
2.00
1.04
3.05
2.04
2.08
2.11
3.11
f1 (ppm)

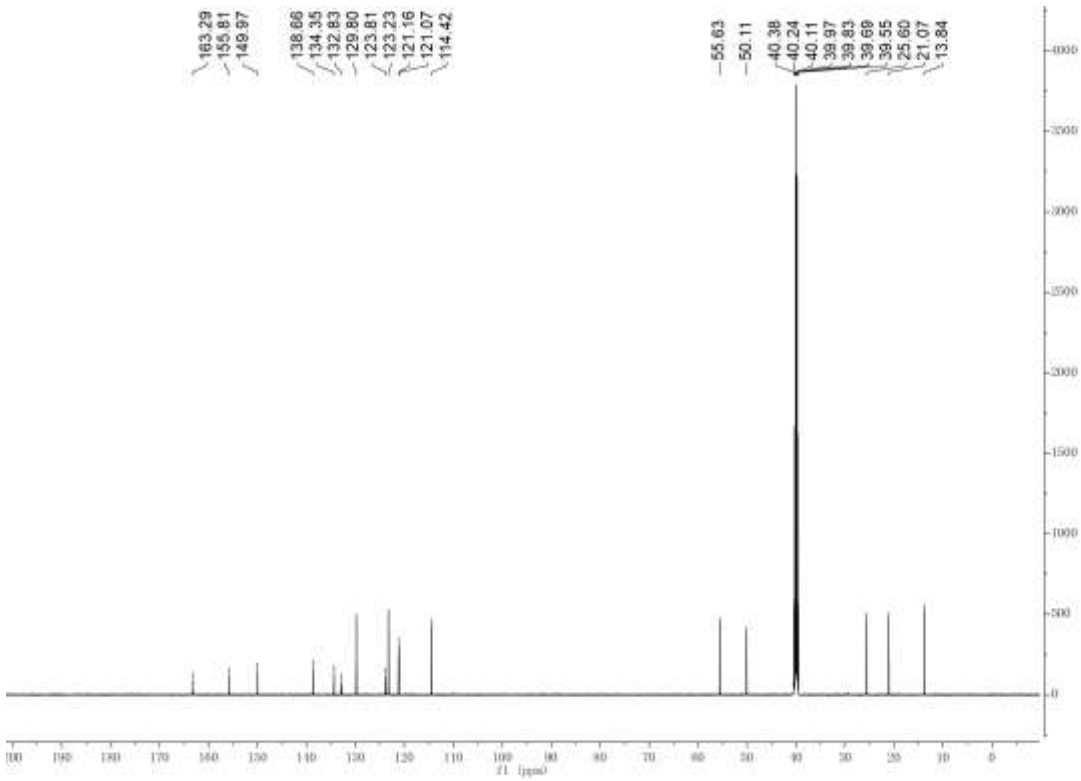
163.29
155.81
149.97
138.66
134.35
132.83
129.80
123.81
123.23
121.16
121.07
114.42
55.63
50.11
40.38
40.24
40.11
39.97
39.83
39.69
39.55
25.60
21.07
13.84
f1 (ppm)

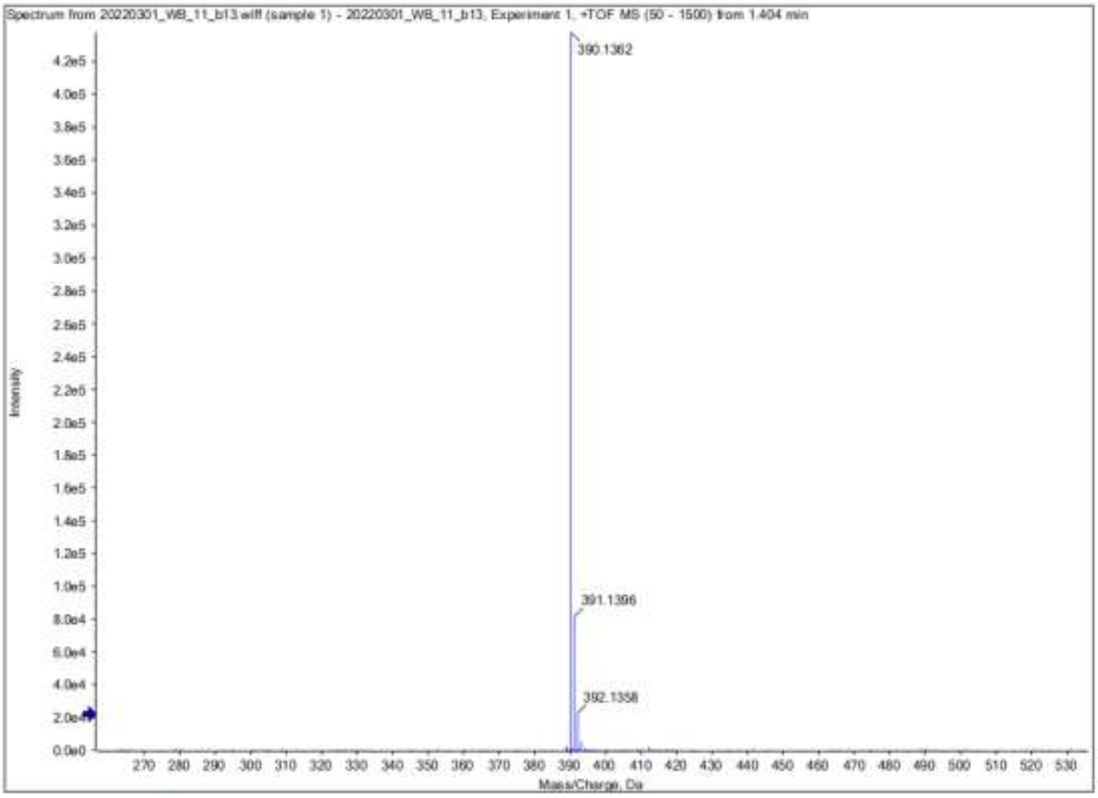

Spartinin C18

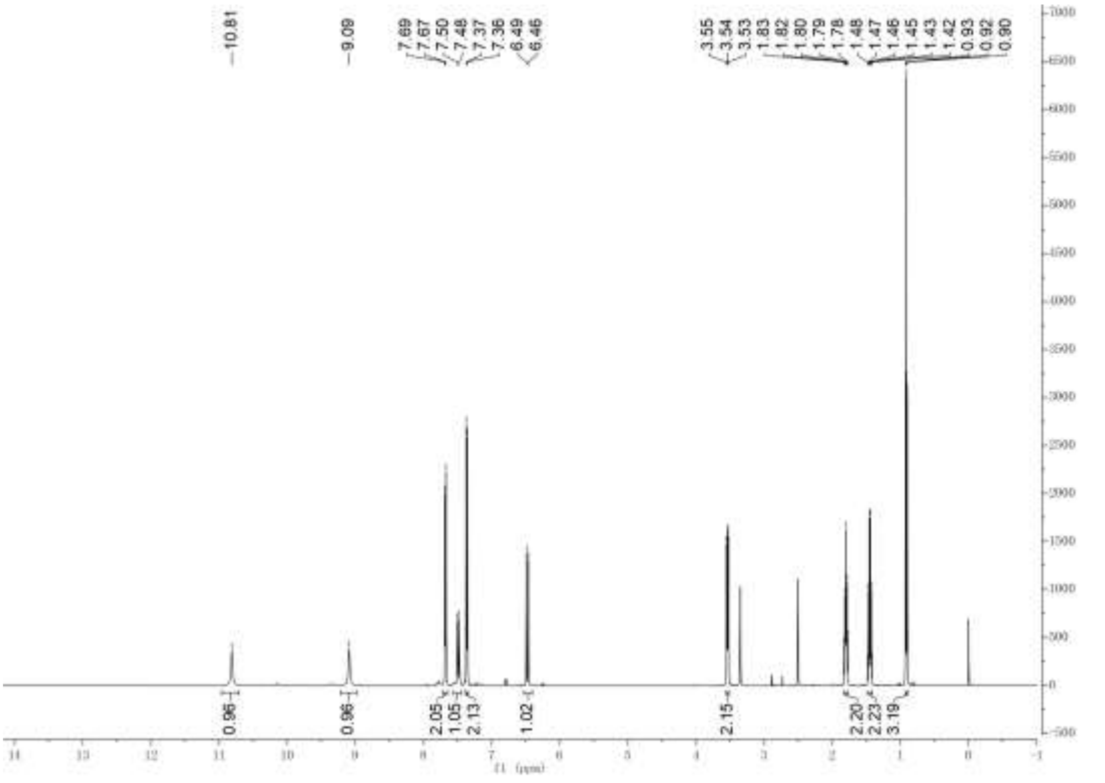

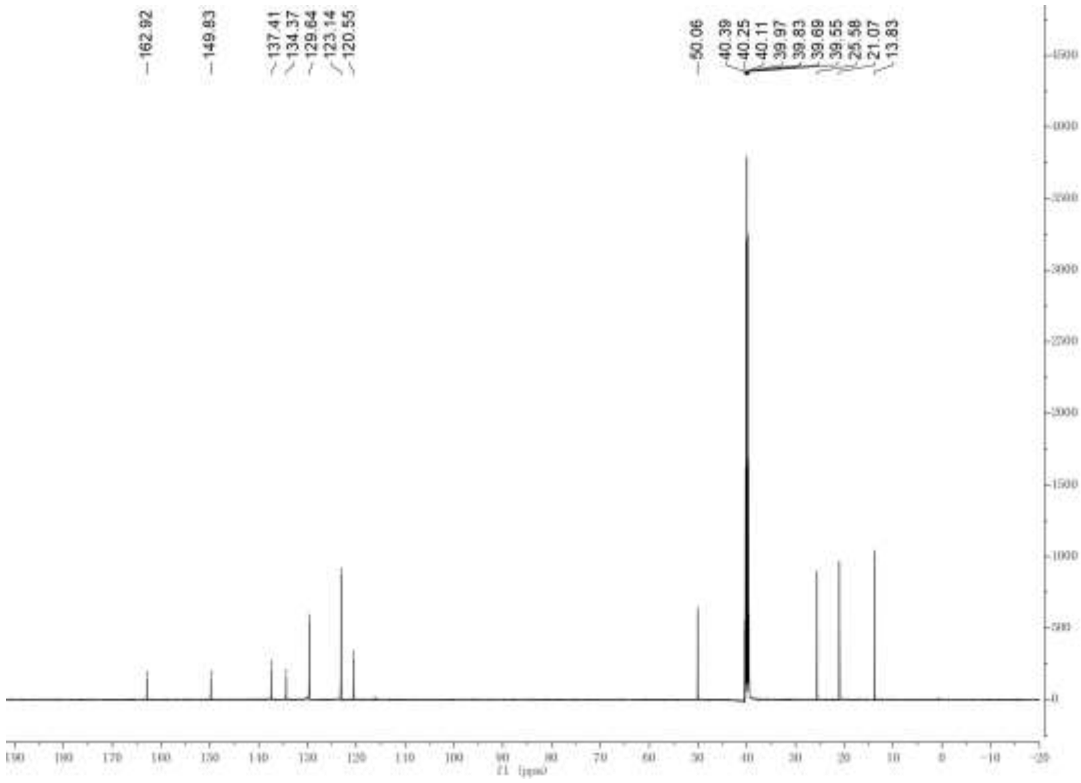

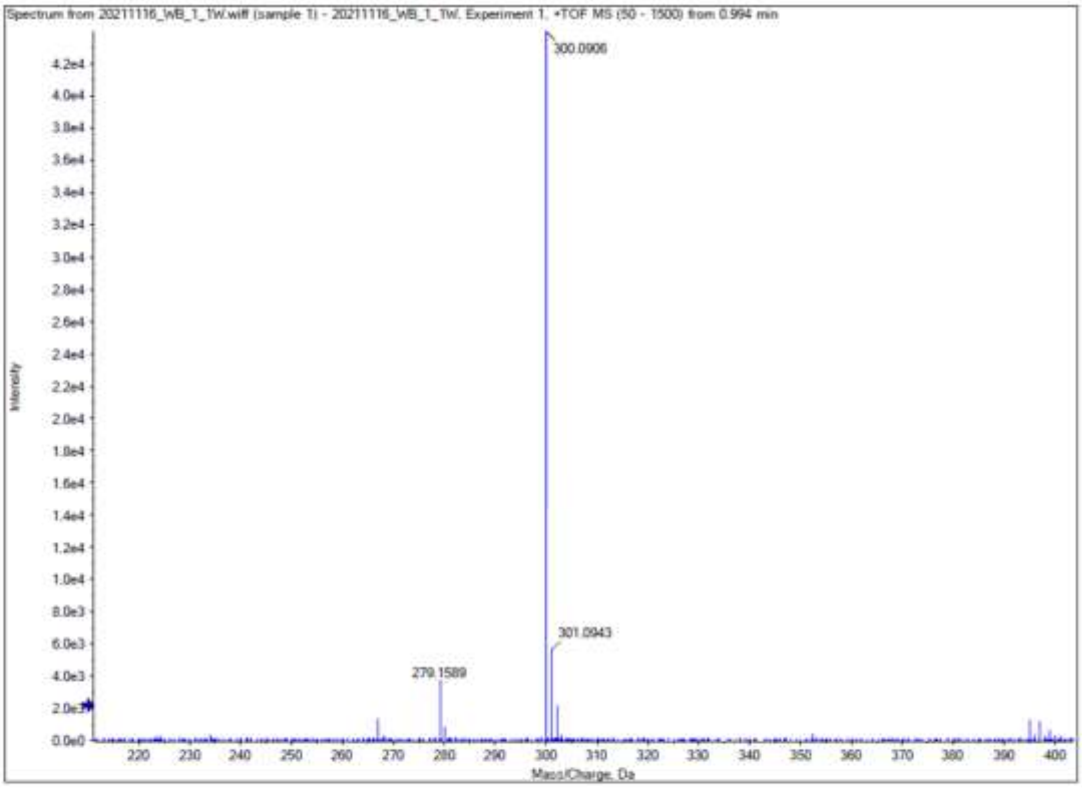

Spartinin C19

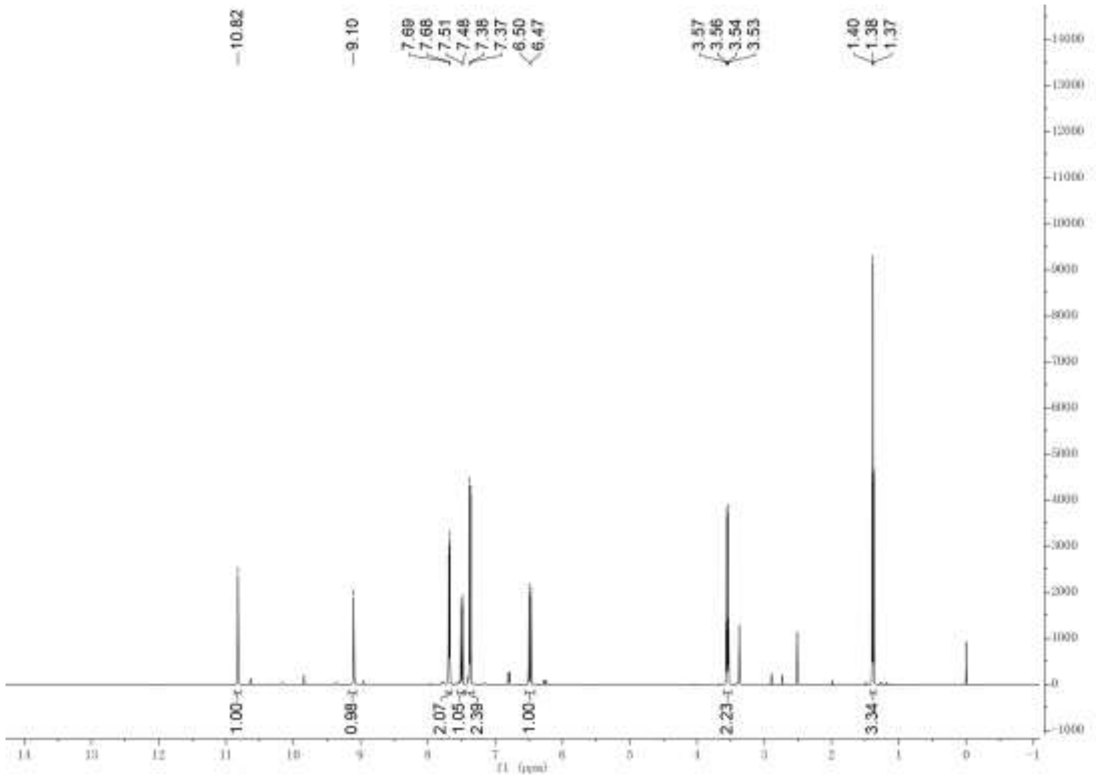
10.82
9.10
7.69
7.68
7.51
7.48
7.38
7.37
6.50
6.47
3.57
3.56
3.54
3.53
1.40
1.38
1.37
1.00
0.98
2.07
1.05
2.39
1.00
2.23
3.34
f1 (ppm)

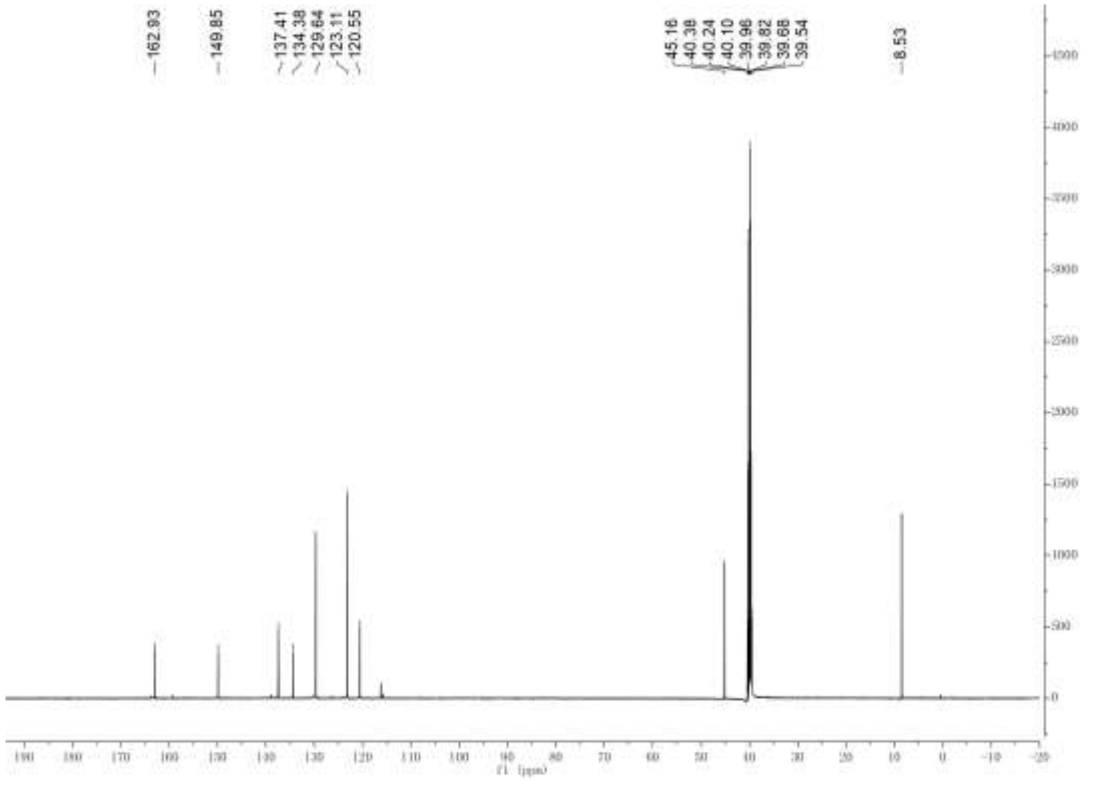
162.93
149.85
137.41
134.38
129.64
123.11
120.55
45.16
40.38
40.24
40.10
39.96
39.82
39.68
39.54
8.53
f1 (ppm)

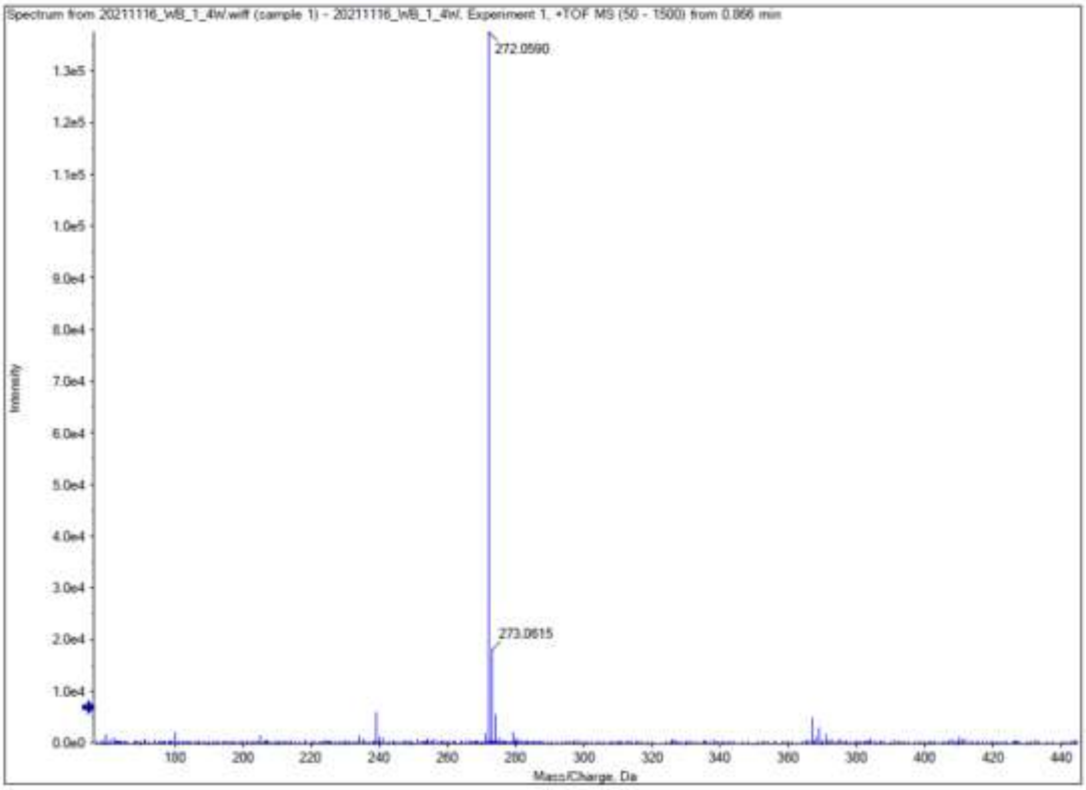

Spartinin C20

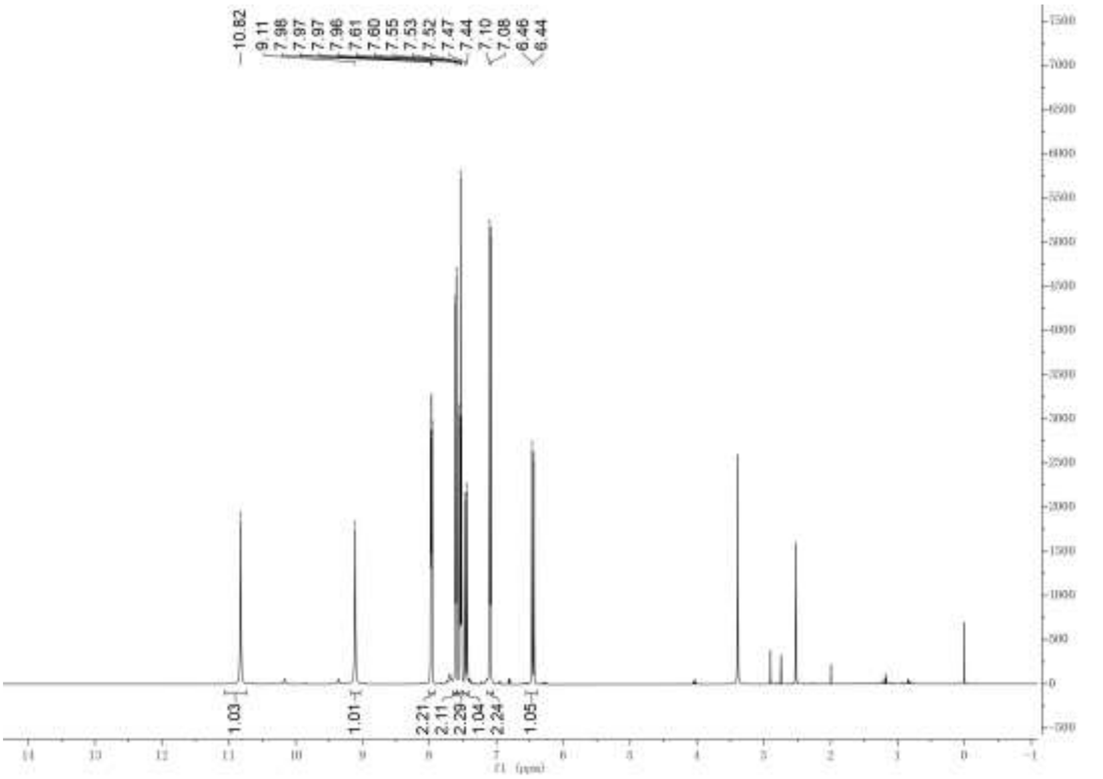

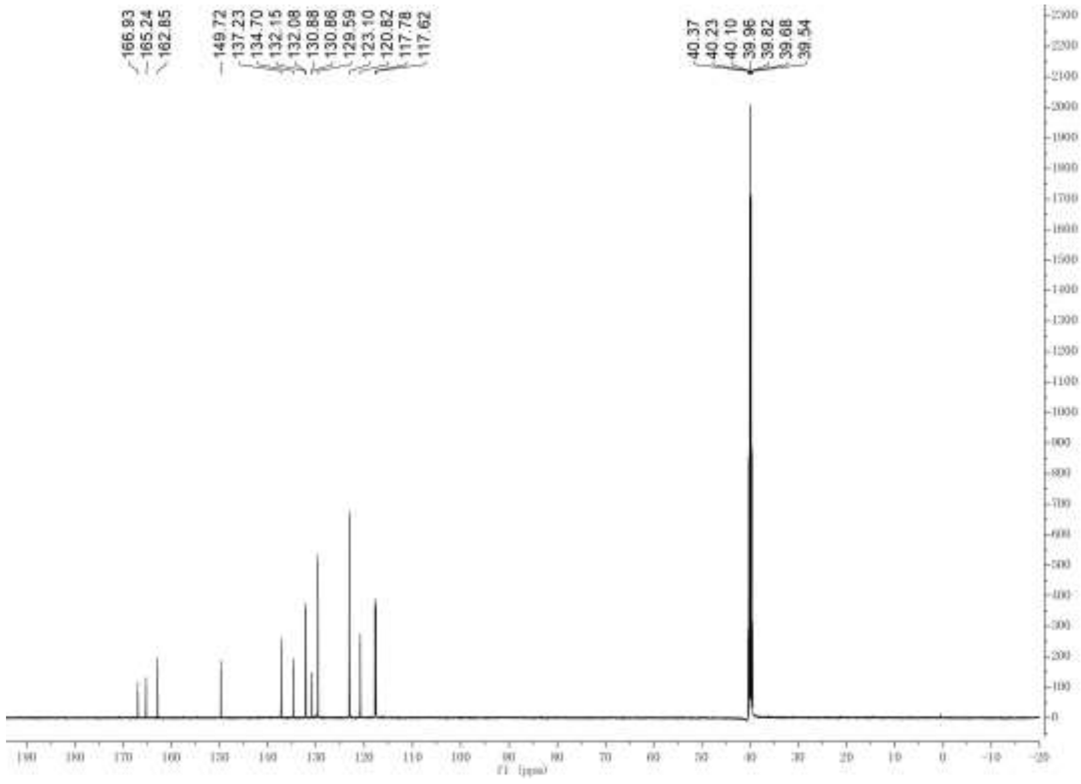
166.93
165.24
162.85
149.72
137.23
134.70
132.15
132.08
130.88
130.86
129.59
123.10
120.82
117.78
117.62
40.37
40.23
40.10
39.96
39.82
39.68
39.54
f1 (ppm)

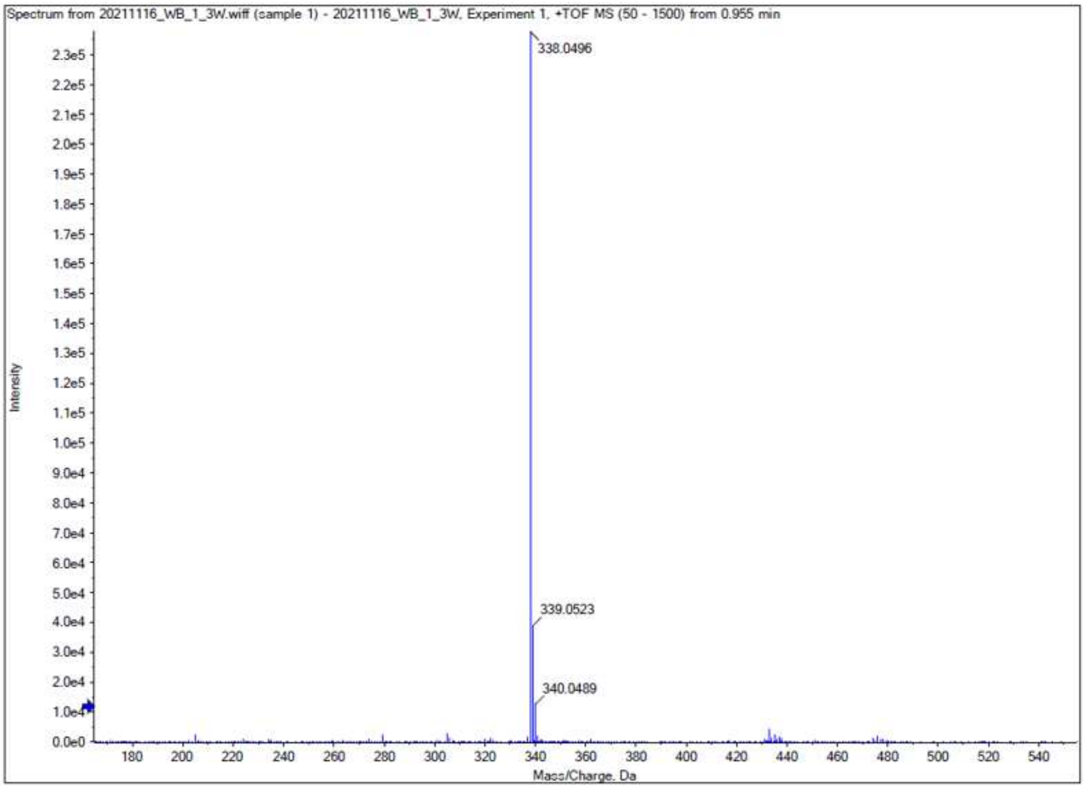
Spectrum from 20211116_WB_1_3W.wiff (sample 1) - 20211116_WB_1_3W, Experiment 1, +TOF MS (50 - 1500) from 0.955 min
338.0496
339.0523
340.0489
Intensity
Mass/Charge, Da

Spartinin C21

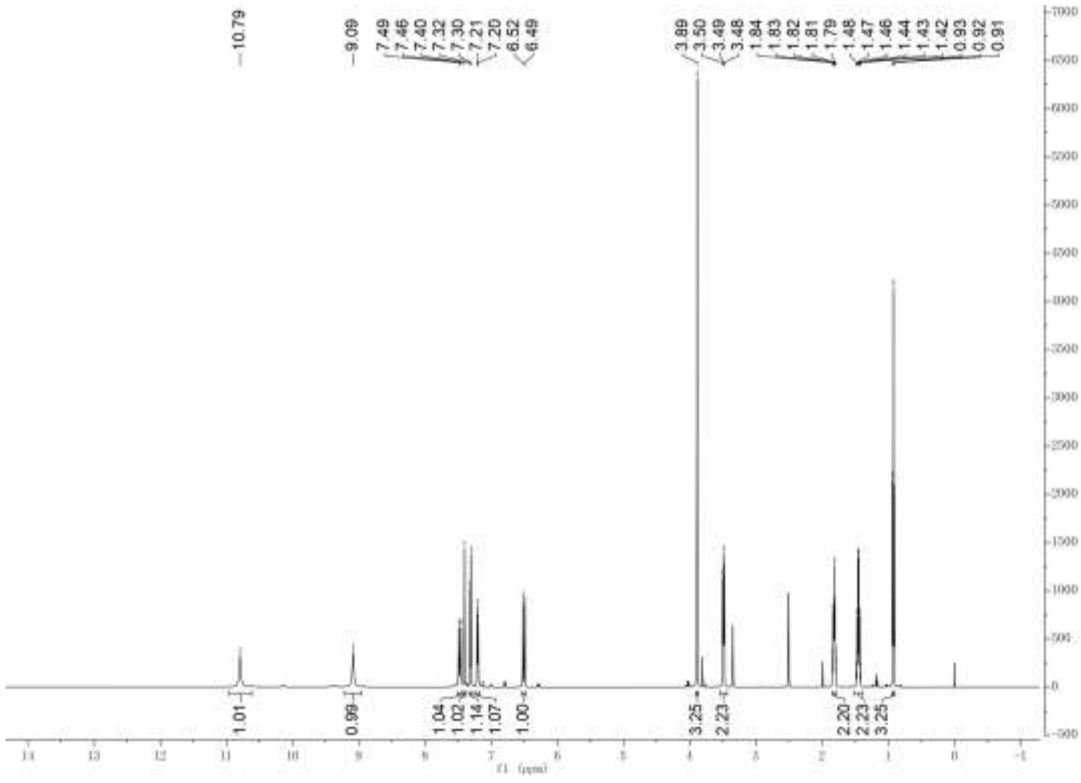

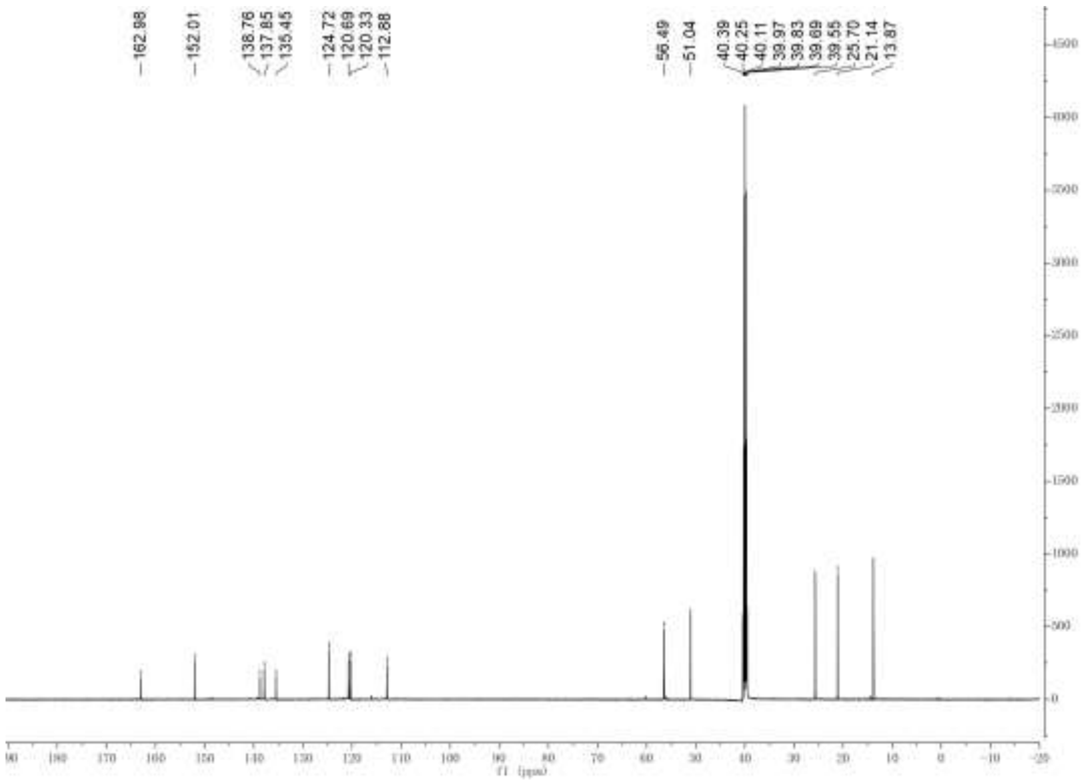

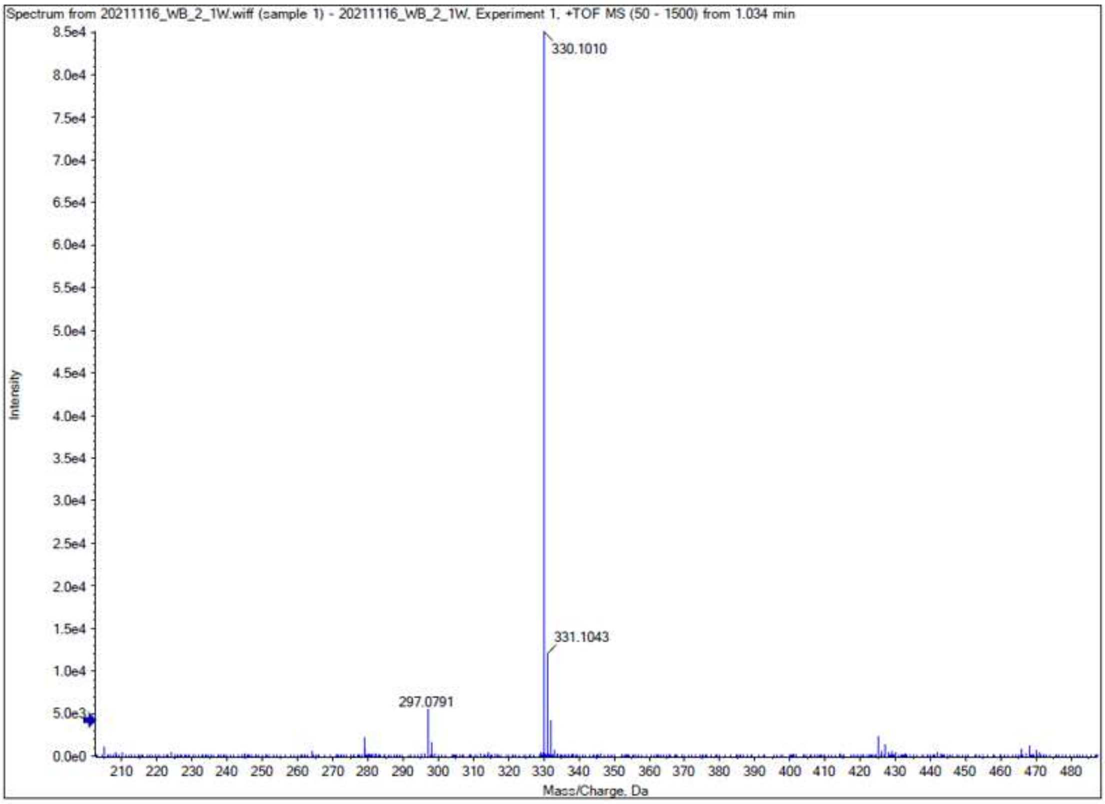

Spartinin C22

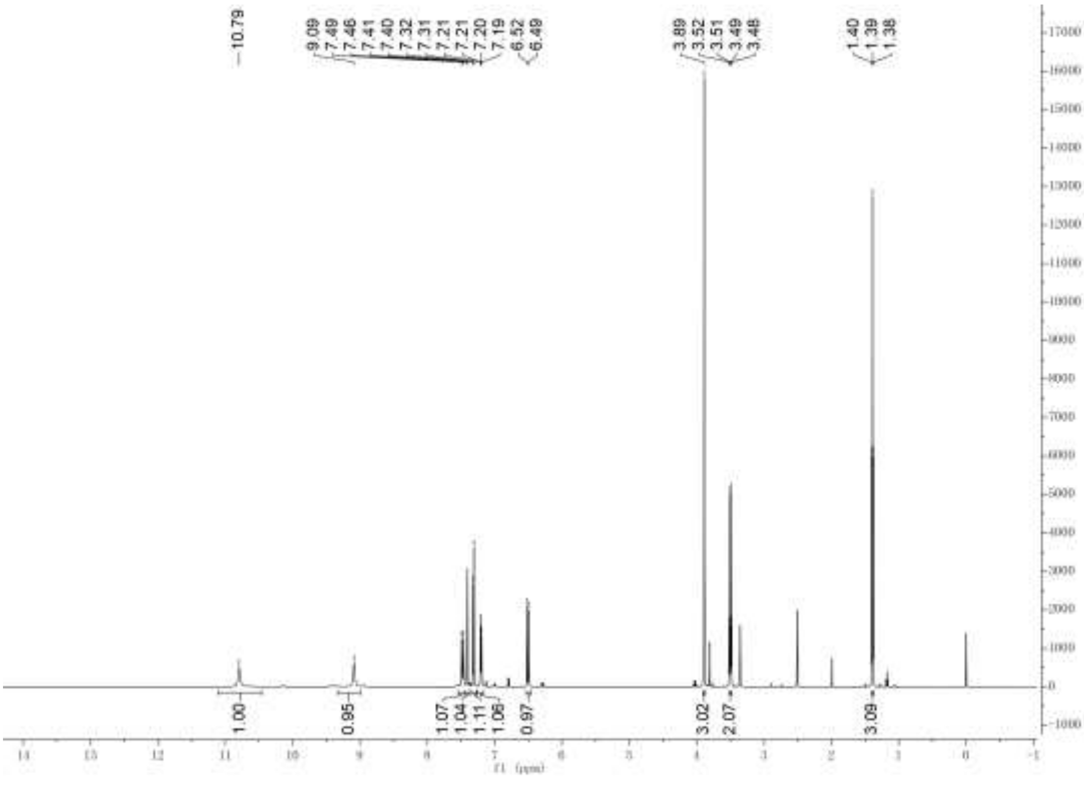

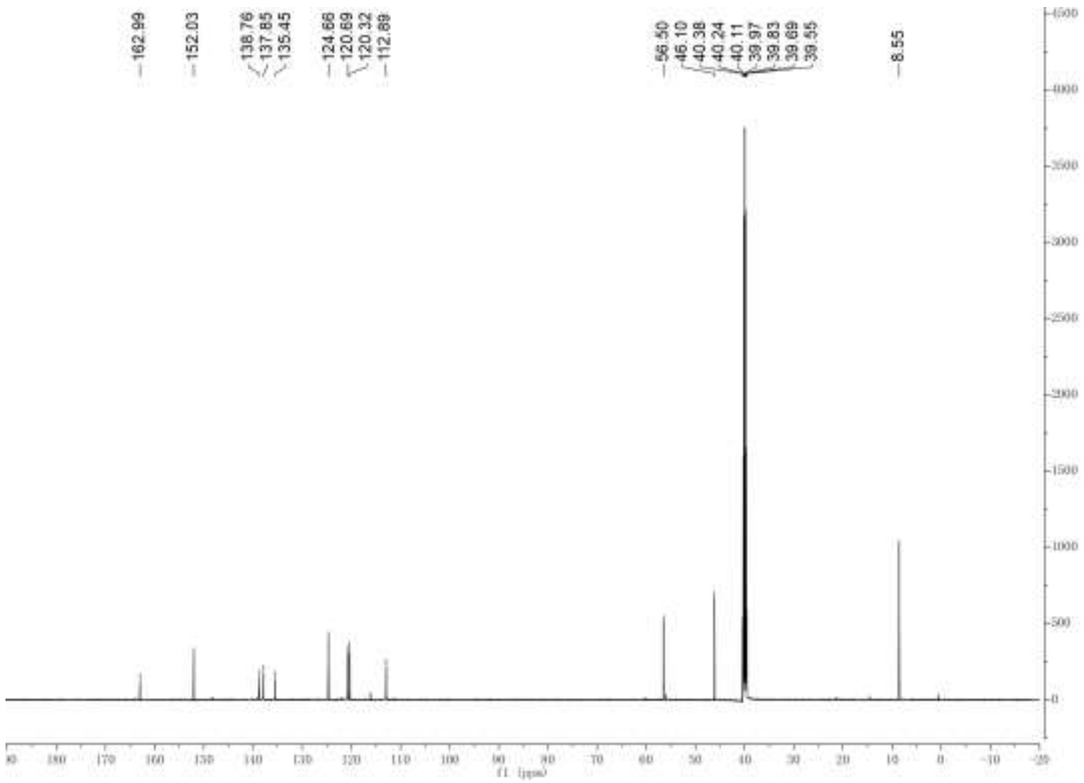
162.99
152.03
138.76
137.85
135.45
124.66
120.69
120.32
112.89
56.50
46.10
40.38
40.24
40.11
39.97
39.83
39.69
39.55
8.55

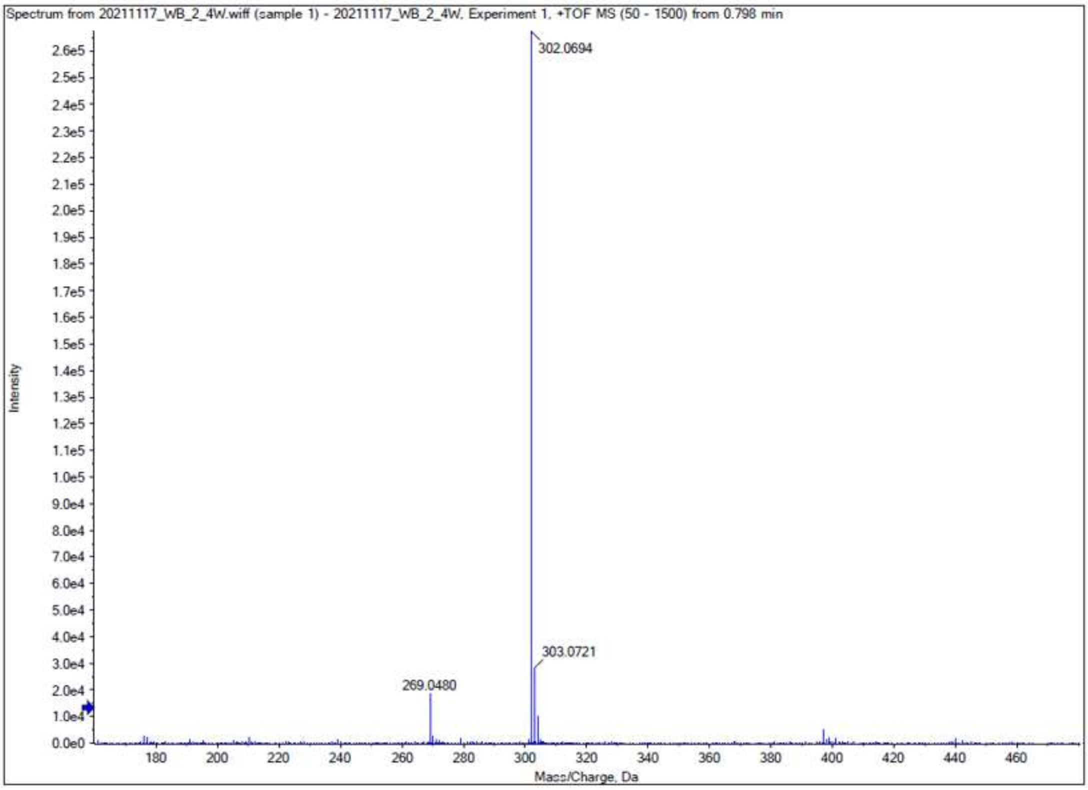
Spectrum from 20211117_WB_2_4W.wiff (sample 1) - 20211117_WB_2_4W, Experiment 1, +TOF MS (50 - 1500) from 0.798 min
302.0694
303.0721
269.0480
Intensity
Mass/Charge, Da

Spartinin C23

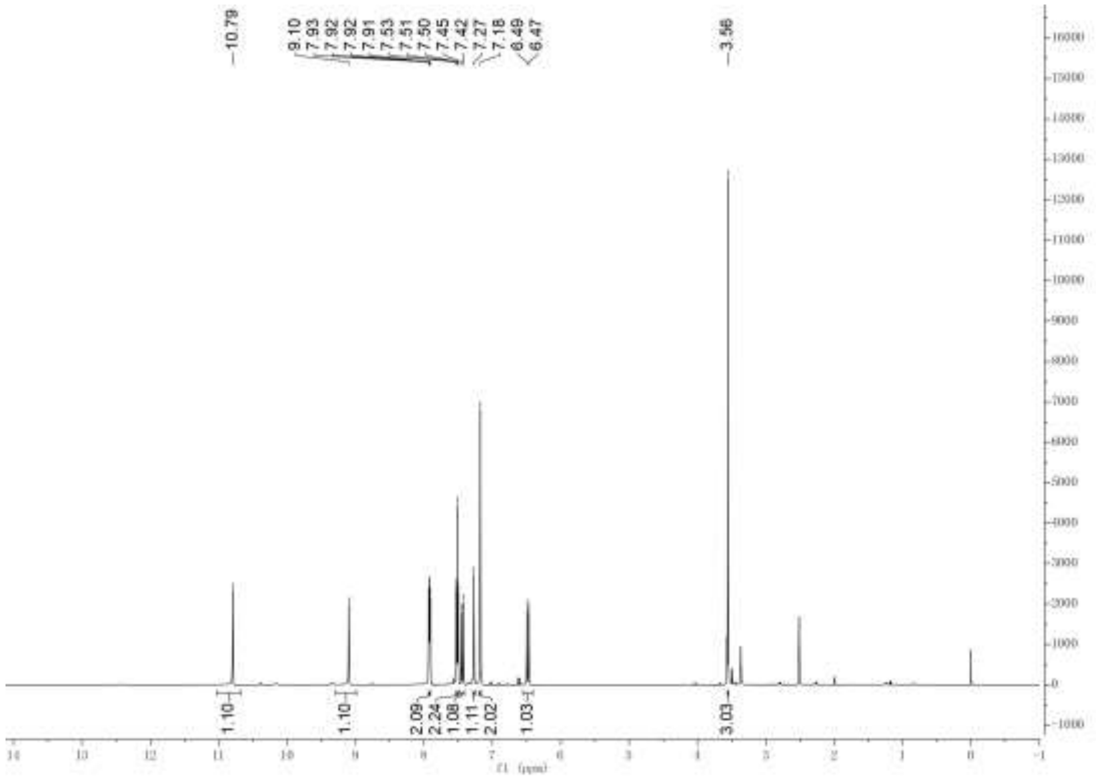

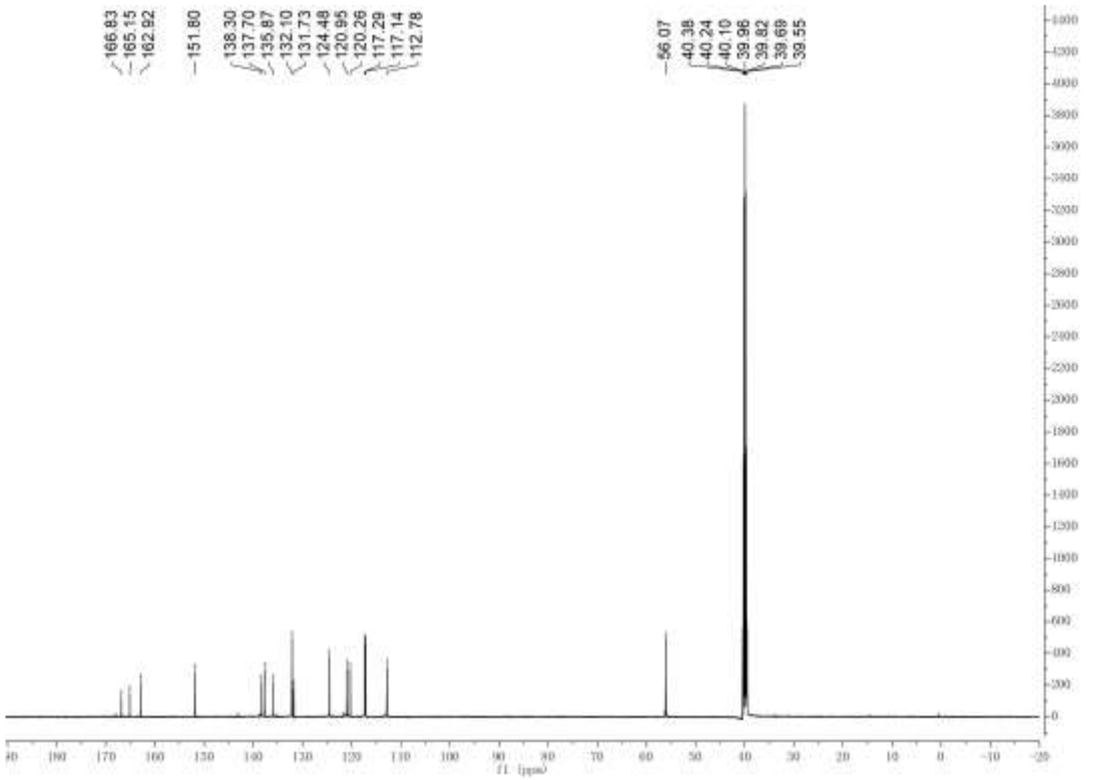

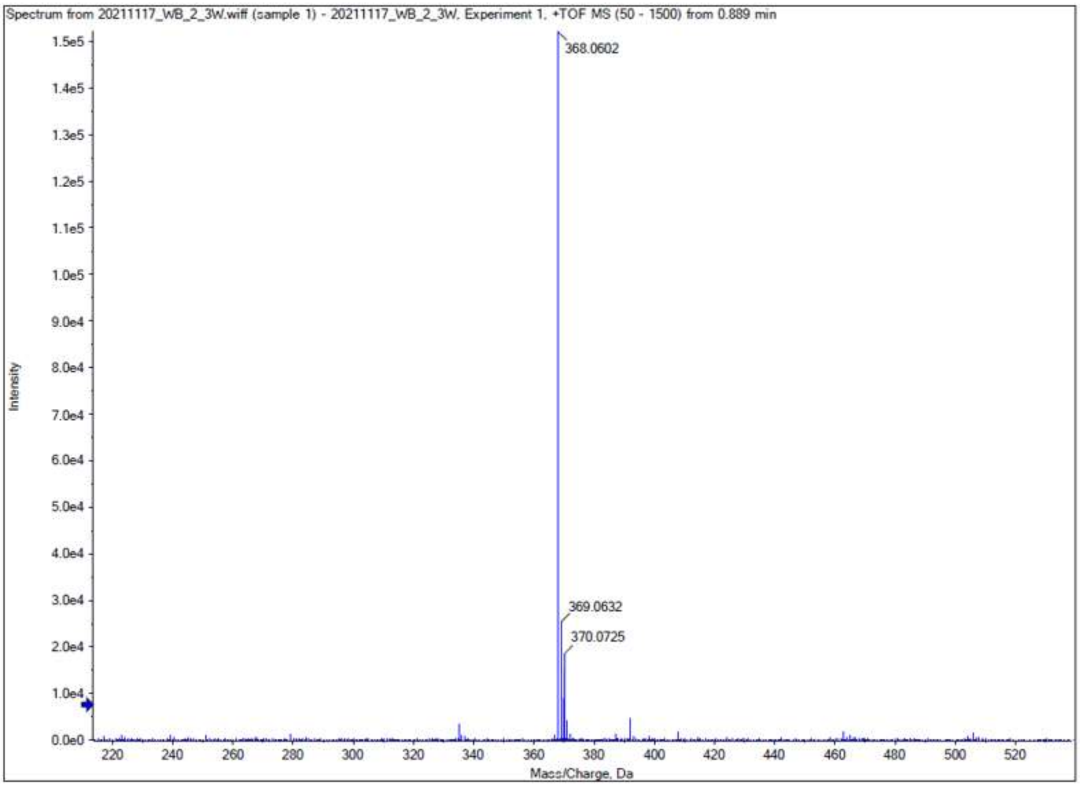

Spartinin C24

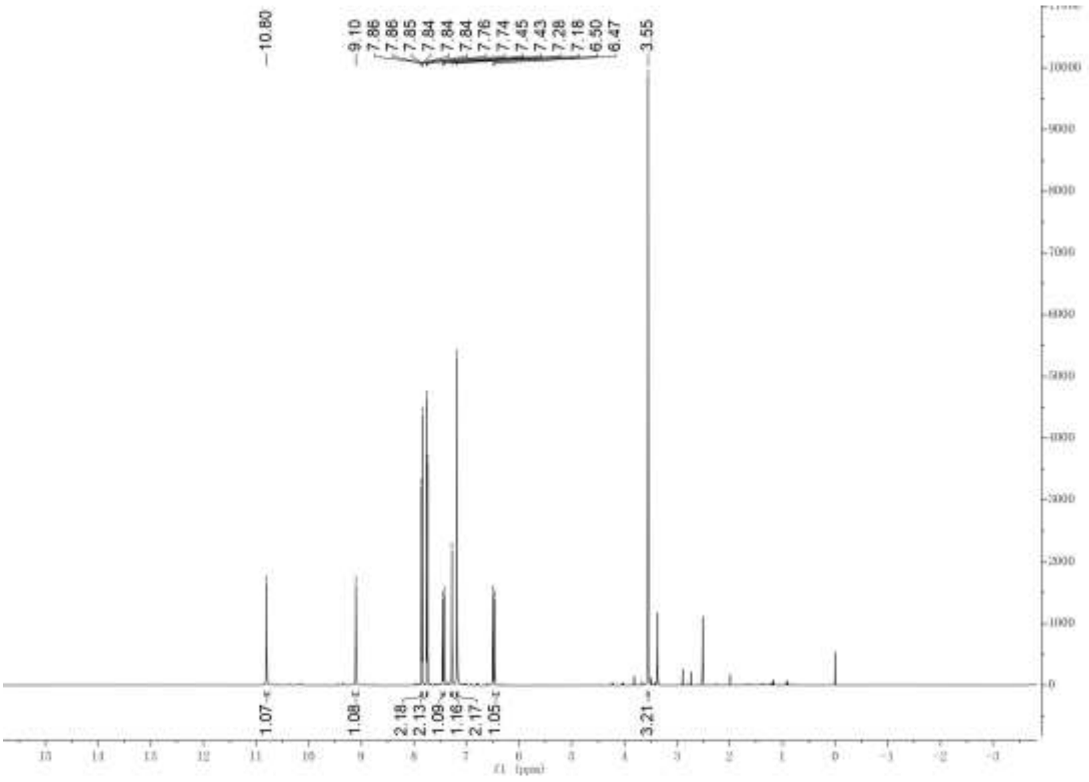

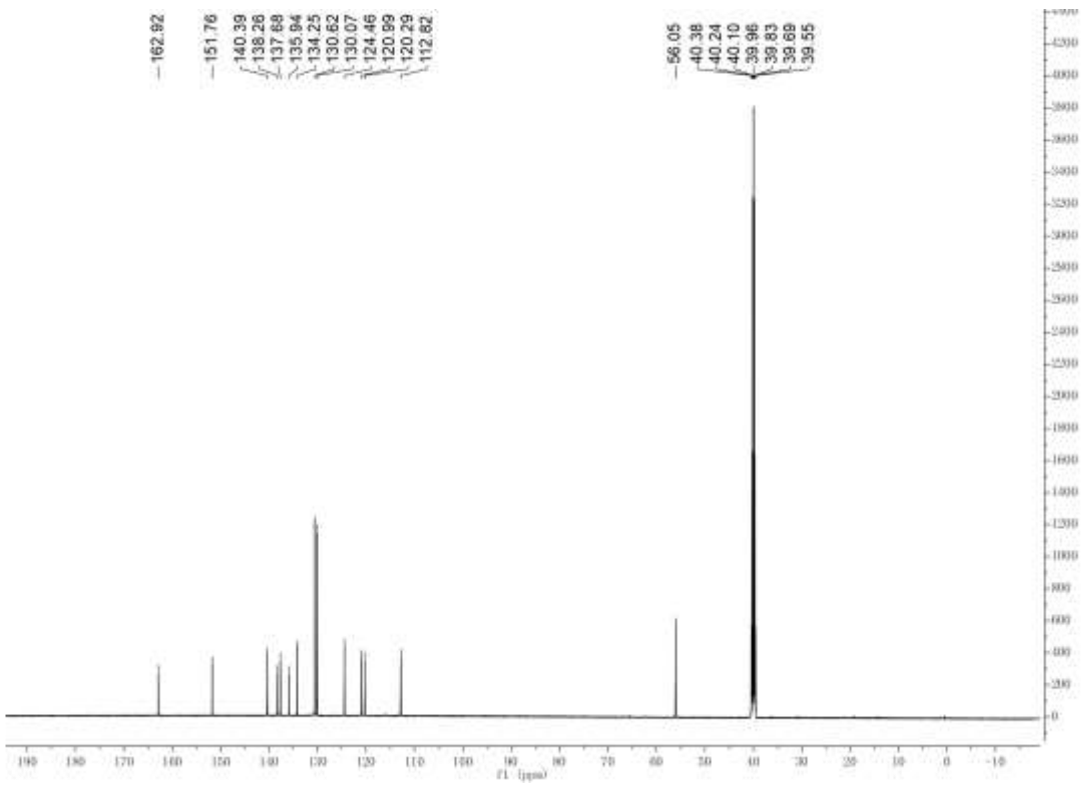
162.92
151.76
140.39
138.26
137.68
135.94
134.25
130.62
130.07
124.46
120.99
120.29
112.82
56.05
40.38
40.24
40.10
39.96
39.83
39.69
39.55
f1 (ppm)

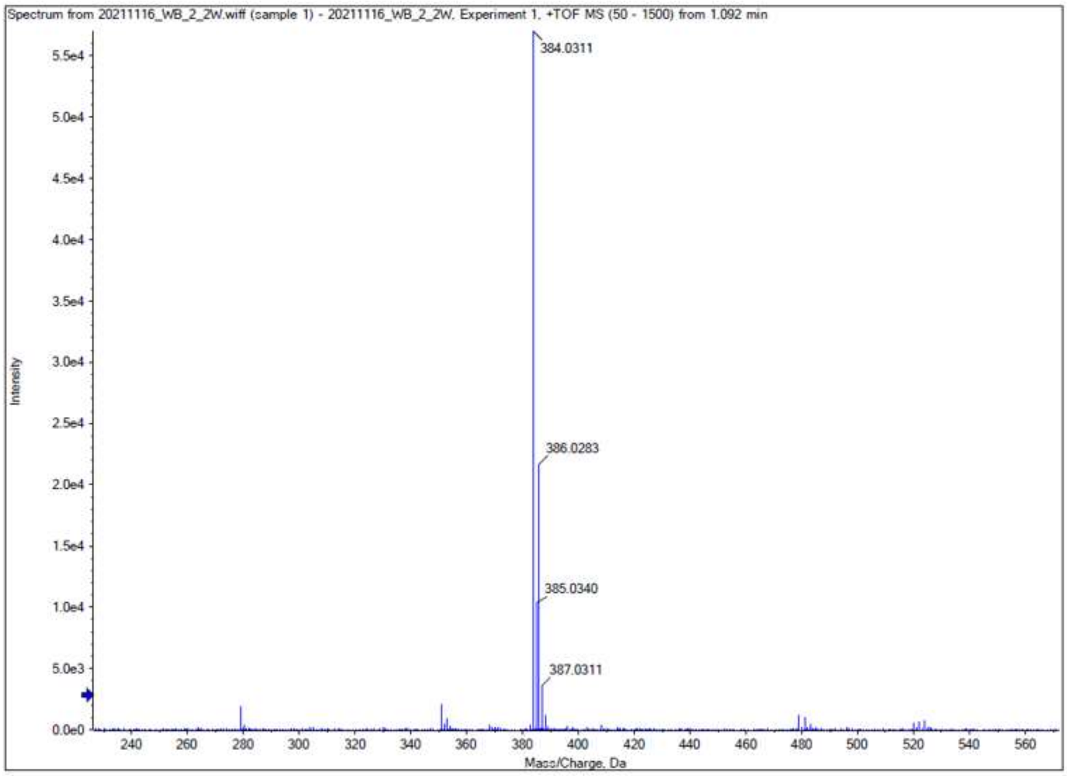
Spectrum from 20211116_WB_2_2W.wiff (sample 1) - 20211116_WB_2_2W, Experiment 1, +TOF MS (50 - 1500) from 1.092 min
384.0311
386.0283
385.0340
387.0311
Intensity
Mass/Charge, Da
